# Supplementary material for: Dynamic Phenomena and Complexation Effects in the α-Lithiation and Asymmetric Functionalization of Azetidines
Source: Molecules. 2022 Apr 29;27(9):2847. doi: 10.3390/molecules27092847 (PMC9103493; doi:10.3390/molecules27092847)
Supplement: Supplementary file 1 [file molecules-27-02847-s001.zip › molecules-1670259-supplementary.pdf]

molecules

SUPPLEMENTARY MATERIALS

Dynamic Phenomena and Complexation Effects in the α-Lithiation and Asymmetric Functionalization of Azetidines

Pantaleo Musci [1], Marco Colella [1], Angela Altomare [2], Giuseppe Romanazzi [3], Nadeem S. Sheikh [4,*], Leonardo Degennaro [1,*] and Renzo Luisi [1,*]

[1] Department of Pharmacy—Drug Sciences, University of Bari "A. Moro", Via E. Orabona 4, 70125 Bari, Italy; pantaleo.musci@uniba.it (P.M.); marco.colella@uniba.it (M.C.)
[2] CNR, Institute of Christallography IC-CNR, via Amendola 127/A, 70125 Bari, Italy; angela.altomare@ic.cnr.it
[3] DICATECh, Politecnico di Bari, Via Orabona 4, 70125 Bari, Italy; giuseppe.romanazzi@poliba.it
[4] Chemical Sciences, Faculty of Science, Universiti Brunei Darussalam, Jalan Tungku Link, Gadong BE1410, Brunei Darussalam
* Correspondence: nadeem.sheikh@ubd.edu.bn (N.S.S.); leonardo.degennaro@uniba.it (L.D.); renzo.luisi@uniba.it (R.L.)

Table of contents

1. General procedures S2–3
2. Characterization of compounds S3–13
3. ^{1}H, ^{13}C, NOESY NMR spectra for Isolated Compounds S14–31
4. X-ray structure and CIF of *minor*-2i S32–36
5. DFT calculations S37–85

1. GENERAL PROCEDURES

GENERAL INFORMATIONS

The chemicals were purchased from Sigma-Aldrich, Fluorochem, TCI Europe, and Alfa Aesar unless otherwise specified. THF and toluene were distilled prior to use. TLC was carried out on a 0.25 mm pre-coated silica gel thick plate (Merck) with a fluorescence indicator F-254; compounds were detected under UV light (at 254 nm). Infrared spectra of the compounds were recorded in reciprocal centimeters (cm^{-1}) by using a PerkinElmer 283 spectrometer. Melting points were measured with Büchi melting point B-545. ^{1}H, ^{13}C, and ^{19}F NMR spectra were recorded with an Agilent 500 spectrometer (500 MHz for ^{1}H, 126 MHz for ^{13}C). The center of the (residual) solvent signal was used as an internal standard (δ 7.26 ppm for ^{1}H in $CDCl_3$ and δ 77.00 ppm for ^{13}C in $CDCl_3$). Spin–spin coupling constants (*J*) are given in Hz. As much as possible, the unambiguous assignment of all resonances was performed by the combined application of 2D NMR techniques, *i.e.*, HSQC and COSY experiments. Data are reported as follows: chemical shift [multiplicity (s = singlet, d = doublet, t = triplet, q = quartet, p = pentet, m = multiplet and bs = broad singlet), coupling constant (in Hz), integration, and assignment]. High resolution mass spectrometry (HRMS) spectra were performed on an Agilent 6530 accurate mass Q-TOF instrument and Excalibur data system. Enantiomeric excess was assessed by HPLC (Chiralcel ADH, Lux_1-Cellulose column). Diastereomeric ratio was assessed by ^{1}H NMR analysis on the reaction crude. Silica (70–230 mesh and 230–400 mesh) and Alumina (standard grade, 58 Å pore size) were used for flash chromatography on glass columns.

GENERAL PROCEDURE FOR α-LITHIATION/ ELECTROPHILE TRAPPING SEQUENCE OF 1 (GP1)

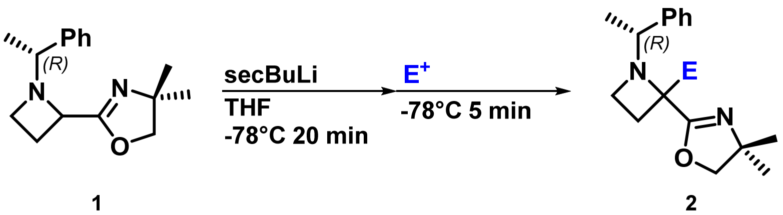

To a stirred solution of **1** (50 mg, 0.19 mmol) in dry THF (3 mL) at −78°C, *sec*-BuLi (1.3M in cyclohexane, 0.292 mL) was added dropwise. After 20 min, electrophile (0.42 mmol, neat) was added and the solution was stirred for an additional hour. The reaction was quenched with NH_4Cl 1M (1mL). The mixture was poured into water (5 mL) and extracted with AcOEt (3 × 5 mL). The combined organic layers were dried over Na_2SO_4, filtered, and concentrated under vacuum. Column chromatography on alumina afforded the desired product.

GENERAL PROCEDURE FOR NUCLEOPHILIC ATTACK (GP2)

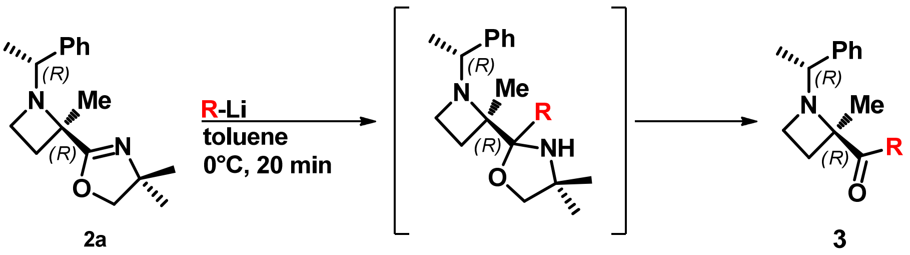

A solution of 4,4-dimethyl-2-((*R*)-2-methyl-1-((*R*)-1-phenylethyl)azetidin-2-yl)-4,5-dihydrooxazole (40 mg, 0.15 mmol) in dry toluene (3.5 mL) was cooled at 0°C and

organolithium (R-Li, 0.6 mmol) was added dropwise. The reaction was stirred for 20 min and quenched with NH_4Cl 1M (1mL). The mixture was poured into water (5 mL) and extracted with AcOEt (3 × 5 mL). The collected organic phases were dried over Na_2SO_4. Column chromatography on silica gel afforded the desired product 3.

IN-SITU FT-IR EXPERIMENT

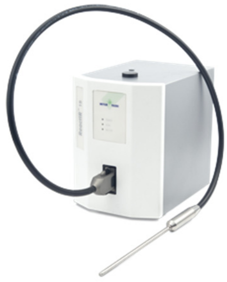

Figure S1. Mettler-Toledo ReactIR 15 equipped with diamond probe.

In-situ FT-IR spectroscopic monitoring was performed with Mettler-Toledo ReactIR 15 equipped with an AgX Fiber Conduit (9.5 mm AgX DiComp) diamond probe.

The experiment was performed in a two-necked flask equipped with the React-IR probe. A stirred solution of **1** (50 mg, 0.19 mmol) in dry THF (3 mL) at −78°C, *sec*-BuLi (1.3M in cyclohexane, 0.292 mL) was added dropwise. After 20 min, the electrophile (0.42 mmol) was added, and the solution was stirred for one hour.

The IR spectra were collected every 15 s and elaborated with iC IR 4.3® software. Signals in the range of 1750-1500 cm^{-1} were observed.

2. CHARACTERIZATION OF COMPOUNDS

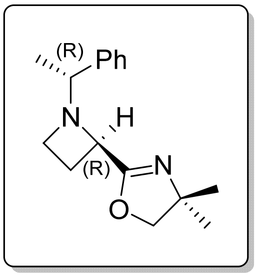

4,4-dimethyl-2-{(R)-1-[(R)-1-phenylethyl]azetidin-2-yl}-4,5-dihydrooxazole (2R,1′R)-1

Substrate **(2R,1′R)-1** was obtained according to the reported procedure as light brown oil [25]. $[\alpha]^{20}_D$ = + 81.20° (c = 1, $CHCl_3$). **^{1}H NMR** (500 MHz, $CDCl_3$) δ 7.35 – 7.26 (m, 4H, Ar-H), 7.24 – 7.20 (m, 1H, Ar-H), 4.04 – 3.98 (m, 2H, OCH_2), 3.87 (t, *J* = 8.1Hz, 1H, NCH), 3.49 – 3.40 (m, 1H, C*H*CH_3), 3.12 – 3.04 (m, 1H, CH_2), 2.82–2.73 (m, 1H, CH_2), 2.38 – 2.29 (m, 1H, CH_2), 2.14 – 2.06 (m, 1H, CH_2), 1.29 (s, 3H, oxazoline-CH_3), 1.28 (s, 3H, oxazoline-CH_3), 1.23 (d, *J* = 6.4 Hz, 3H, CHC*H*$_3$). The data are consistent with literature [25].

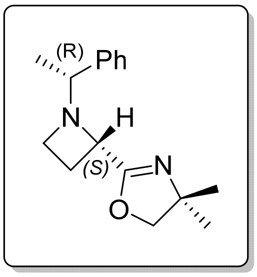

4,4-dimethyl-2-{(S)-1-[(R)-1-phenylethyl]azetidin-2-yl}-4,5-dihydrooxazole (2S,1′R)-1

Substrate **(2S,1′R)-1** was obtained according to the reported procedure as light yellow oil [25]. $[\alpha]^{20}_D$ = −56.30° (c = 1, $CHCl_3$). **^{1}H NMR** (500 MHz, $CDCl_3$) δ 7.34–7.16 (m, 5H, Ar-H overlapping $CHCl_3$), 3.76–3.68 (m, 2H, 1H OCH_2 and 1H NCH), 3.58 – 3.50 (m, 2H, 1H OCH_2 and 1H NCH_2), 3.34 (q, *J* = 6.6 Hz, 1H, C*H*CH_3), 2.99 (q, *J* = 7.7Hz, 1H, NCH_2), 2.38–2.27 (m, 1H, NCH_2C*H*$_2$), 2.13–2.04 (m, 1H, NCH_2C*H*$_2$), 1.26 (d, *J* = 6.6Hz, 3H, C*H*$_3$CH), 1.03 (s, 1H, oxazoline-CH_3), 0.81 (s, 1H, oxazoline-CH_3). The data are consistent with literature [25].

4,4-dimethyl-2-((R)-1-((R)-1-phenylethyl)azetidin-2D-yl)-4,5-dihydrooxazole 2a

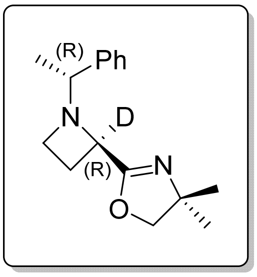

Following GP1 with CD_3OD as an electrophile, compound **2a** was obtained as colourless oil (47 mg, 95%). dr = 90:10. $[\alpha]^{20}_D$ = + 84.09° (c = 1, $CHCl_3$). R_f = 0.4 (AcOEt/hexane 60:40). **FT-IR** (film, cm^{-1}) 2967, 2928, 2849, 1659, 1520, 1454, 1364, 1311, 1281, 1179, 1071, 1035, 994, 863, 813, 762, 701. **^{1}H NMR** (500 MHz, $CDCl_3$) δ 7.32 – 7.23 (m, 4H, Ar-H overlapping $CHCl_3$), 7.21 – 7.17 (m, 1H, Ar-H), 4.02 – 3.95 (AB system, *J* = 8.1 Hz, 2H, OCH_2), 3.41 (q, *J* = 6.5 Hz, 1H, C*H*CH_3), 3.04 (ddd, *J* = 8.4, 7.2, 2.4 Hz, 1H, NCH_2), 2.74 (dt, *J* = 9.3, 7.8 Hz, 1H, NCH_2), 2.30 (dd, *J* = 18.7, 9.4 Hz, 1H, NCH_2CH_2), 2.09 – 2.03 (m, 1H, NCH_2CH_2), 1.26 (s, 3H, oxazoline-CH_3), 1.25 (s, 3H, oxazoline-CH_3), 1.20 (d, *J* = 6.5 Hz, 3H, $CHCH_3$). **^{13}C NMR** (126 MHz, $CDCl_3$) δ 166.3 (C=N), 143.0 (Ar-C_q), 128.3 (2 x Ar-C), 127.6 (2 x Ar-C), 127.2 (Ar-C), 79.4 (OCH_2), 68.1 (CHPh), 67.0 (C_q), 60.8 (t, C-D, J_{C-D} = 21.4), 50.2 (NCH_2), 28.23 (oxazoline-CH_3), 28.17 (oxazoline-CH_3), 21.1 (CH_3CH), 21.0 (NCH_2CH_2). **HRMS** calculated for $C_{16}H_{24}DN_2O_2$ $[M+H_3O]^+$ 278.1973; found 278.1974.

4,4-dimethyl-2-((R)-2-methyl-1-((R)-1-phenylethyl)azetidin-2-yl)-4,5-dihydrooxazole 2b

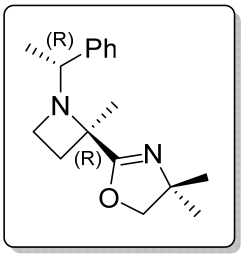

Following GP1 with iodomethane as an electrophile, compound **2b** was obtained as pale-yellow oil (48 mg, 92%). dr = 90:10, er = 95:5 (ADH, 99.5:0.5 Hex:iPrOH + 0.2% DEA, 0.5mL/min). $[\alpha]^{20}_D$ = + 86.35° (c = 1, $CHCl_3$). R_f = 0.5 (AcOEt/hexane 50:50). **FT-IR** (film, cm^{-1}) 2968, 2928, 1650, 1453, 1366, 1317, 1239, 1189, 1111, 993, 974, 765, 701, 623. **^{1}H NMR** (300 MHz, $CDCl_3$) δ 7.37 – 7.18 (m, 5H, Ar-H), 4.07 – 3.98 (AB system, *J* = 8.0 Hz, 2H, OCH_2), 3.69 (q, *J* = 6.2 Hz, 1H, CHPh), 3.16 – 3.08 (m, 2H, NCH_2), 2.51 – 2.46 (m, 1H, NCH_2CH_2), 1.88 – 1.83 (m, 1H, NCH_2CH_2), 1.42 (s, 3H, azetidine-CH_3), 1.33 (s, 3H, oxazoline-CH_3), 1.30 (s, 3H, oxazoline-CH_3), 1.19 (d, *J* = 6.2 Hz, 3H, C*H*$_3$CH). **^{13}C NMR** (126 MHz, $CDCl_3$) δ 168.4 (C=N), 144.0 (Ar-C_q), 128.1 (2 x Ar-C), 128.0 (2 x Ar-C), 127.2 (Ar-C), 79.1 (OCH_2), 67.1 (C_q), 63.4 (C_q), 60.8 (CHPh), 48.3 (NCH_2), 28.9 (NCH_2CH_2), 28.6 (oxazoline-CH_3), 28.2 (oxazoline-CH_3), 22.0 ($CHCH_3$), 20.5 (azetidine-CH_3). **HRMS** calculated for $C_{17}H_{25}N_2O$ $[M+H]^+$ 273.1967; found 273.1965.

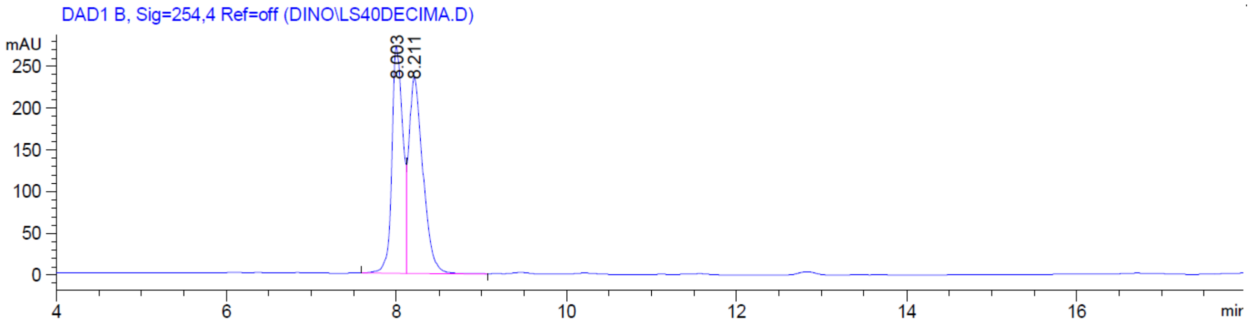

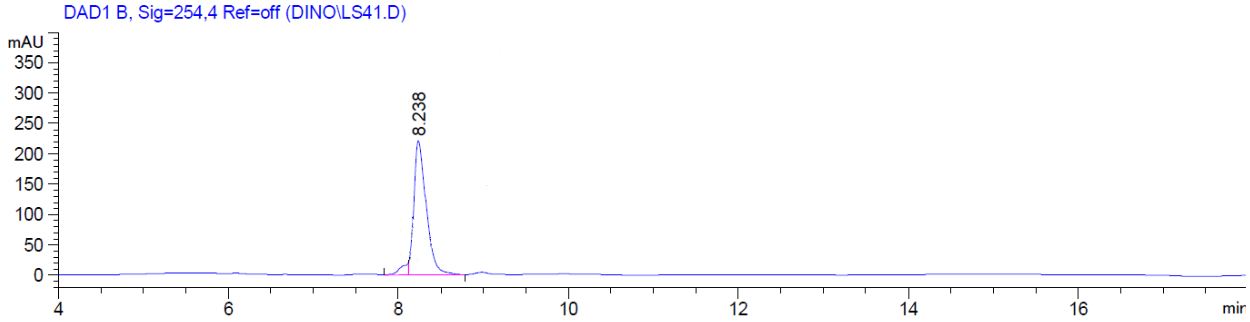

Signal 2: DAD1 B, Sig=254,4 Ref=off

Peak #	RetTime [min]	Type	Width [min]	Area [mAU*s]	Height [mAU]	Area %
1	8.003	BV	0.1317	2418.59253	272.04041	47.2376
2	8.211	VB	0.1703	2701.46655	234.74211	52.7624

Signal 2: DAD1 B, Sig=254,4 Ref=off

Peak #	RetTime [min]	Type	Width [min]	Area [mAU*s]	Height [mAU]	Area %
1	8.109	MF	0.1224	124.95995	17.00868	5.2775
2	8.238	FM	0.1691	2242.81006	221.07571	94.7225

2-((R)-2-ethyl-1-((R)-1-phenylethyl)azetidin-2-yl)-4,4-dimethyl-4,5-dihydrooxazole 2c

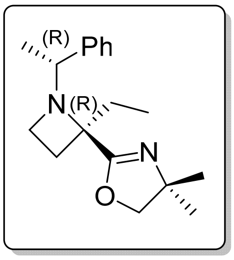

Following GP1 with iodoethane as an electrophile, compound **2c** was obtained as pale-yellow oil (52 mg, 95%). dr = 90:10, er > 99:1 (LUX-1, 99:1 Hex:iPrOH + 0.5% DEA, 0.5mL/min). $[\alpha]^{20}_{D}$ = + 72.50° (c = 1, $CHCl_3$). R_f = 0.5 (hexane/AcOEt 60:40). **FT-IR** (film, cm^{-1}) 2970, 2910, 1643, 1440, 1370, 1305, 1247, 1170, 1002, 987, 965, 780, 704, 624. **^{1}H NMR** (500 MHz, $CDCl_3$) δ 7.31 – 7.28 (m, 2H, Ar-H), 7.26 – 7.21 (m, 2H, Ar-H), 7.21 – 7.16 (m, 1H, Ar-H), 3.94 (s, 2H, OCH_2), 3.57 (q, *J* = 6.5 Hz, 1H, C*H*CH_3), 3.28 – 3.21 (m, 2H, NCH_2), 2.44 – 2.37 (m, 1H, NCH_2CH_2), 2.03 – 1.96 (m, 1H, NCH_2CH_2), 1.68 – 1.60 (m, 1H, CH_2CH_3), 1.59 – 1.51 (m, 1H, CH_2CH_3), 1.35 (s, 3H, oxazoline-CH_3), 1.29 (s, 3H, oxazoline-CH_3), 1.16 (d, *J* = 6.5 Hz, 3H, $CHCH_3$), 0.72 (t, *J* = 7.4 Hz, 3H, CH_2CH_3). **^{13}C NMR** (126 MHz, $CDCl_3$) δ 166.0 (C=N), 145.5 (Ar-C_q), 127.9 (2 x Ar-C), 127.6 (2 x Ar-C), 127.0 (Ar-C), 78.6 (OCH_2), 68.9 (C_q), 67.3 (C_q), 61.1 (CHPh), 48.5 (NCH_2), 29.5 (CH_2CH_3), 29.0 (oxazoline-CH_3), 28.5 (oxazoline-CH_3), 25.8 (NCH_2CH_2), 22.5 ($CHCH_3$), 8.2 (CH_2CH_3). **HRMS** calculated for $C_{18}H_{27}N_2O$ $[M+H]^+$ 287.2123; found: 287.2120.

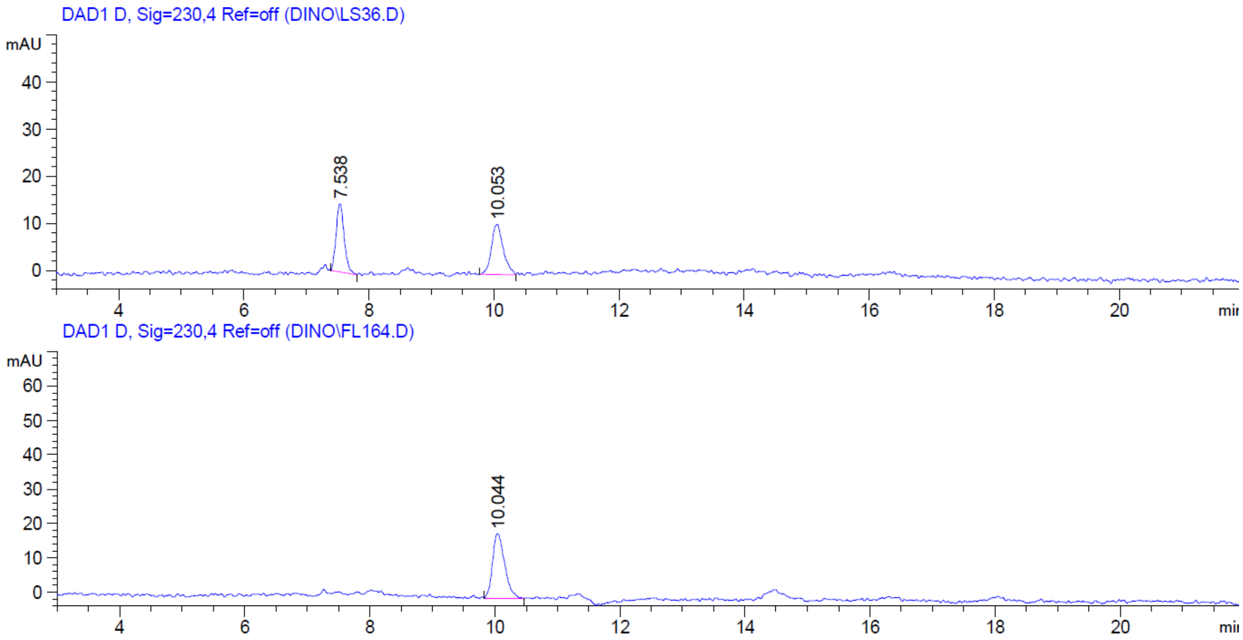

Signal 3: DAD1 D, Sig=230,4 Ref=off

Peak #	RetTime [min]	Type	Width [min]	Area [mAU*s]	Height [mAU]	Area %
1	7.538	BV	0.1344	129.54169	14.47675	47.8231
2	10.053	BV	0.1681	141.33537	10.69755	52.1769

Signal 3: DAD1 D, Sig=230,4 Ref=off

Peak #	RetTime [min]	Type	Width [min]	Area [mAU*s]	Height [mAU]	Area %
1	10.044	VB	0.1995	255.21564	18.90306	100.0000

2-((R)-2-propyl-1-((R)-1-phenylethyl)azetidin-2-yl)-4,4-dimethyl-4,5-dihydrooxazole 2d

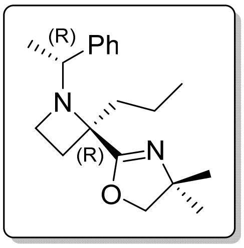

Following GP1 with iodopropane as an electrophile, compound **2d** was obtained as pale-yellow oil (29 mg, 50%). dr = 90:10, er > 99:1 (LUX-1, 99.5:0.5 Hex:iPrOH + 0.2% DEA, 0.5mL/min). $[\alpha]^{20}_{D}$ = + 35.88° (c = 1, $CHCl_3$). R_f = 0.6 (hexane/AcOEt 60:40). **FT-IR** (film, cm^{-1}) 3026, 2962, 2928, 2870, 1648, 1452, 1364, 1350, 1326, 1218, 1117, 761, 700. **^{1}H NMR** (500 MHz, $CDCl_3$) δ 7.32 – 7.28 (m, 2H, Ar-H), 7.27 – 7.22 (m, 2H, Ar-H overlapping $CHCl_3$), 7.21 – 7.17 (m, 1H, Ar-H), 3.93 (s, 2H, OCH_2), 3.57 (q, *J* = 6.5 Hz, 1H, C*H*CH_3), 3.28 – 3.19 (m, 2H, NCH_2), 2.45 – 2.38 (m, 1H, NCH_2CH_2), 2.07 – 1.99 (m, 1H, NCH_2CH_2), 1.62 (td, *J* = 12.0, 5.1 Hz, 1H, $CH_2CH_2CH_3$), 1.45 (td, *J* = 12.6, 4.2 Hz, 1H, $CH_2CH_2CH_3$), 1.36 (s, 3H, oxazoline-CH_3), 1.29 (s, 3H, oxazoline-CH_3), 1.27 – 1.18 (m, 1H, $CH_2CH_2CH_3$), 1.15 (d, *J* = 6.5 Hz, 3H, $CHCH_3$), 1.13 – 1.04 (m, 1H, $CH_2CH_2CH_3$), 0.82 (t, *J* = 7.4 Hz, 3H, $CH_2CH_2CH_3$). **^{13}C NMR** (126 MHz, $CDCl_3$) δ 166.3 (C=N), 145.5 (Ar-C_q), 127.9 (2 x Ar-C), 127.6 (2 x Ar-C), 126.9 (Ar-C), 78.6 (OCH_2), 68.3 (C_q), 67.2 (C_q), 61.1 (CHPh), 48.7 (NCH_2), 38.6 ($CH_2CH_2CH_3$), 29.1 (oxazoline-CH_3), 28.6 (oxazoline-CH_3), 26.3 (NCH_2CH_2), 22.6 ($CHCH_3$), 17.2 ($CH_2CH_2CH_3$), 14.5 ($CH_2CH_2CH_3$). **HRMS** calculated for $C_{19}H_{28}N_2NaO$ $[M+Na]^+$ 323.2099; found 323.2097.

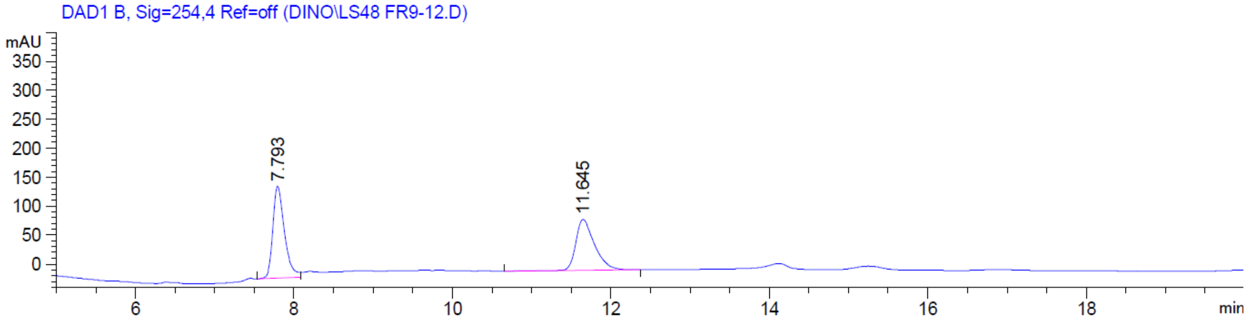

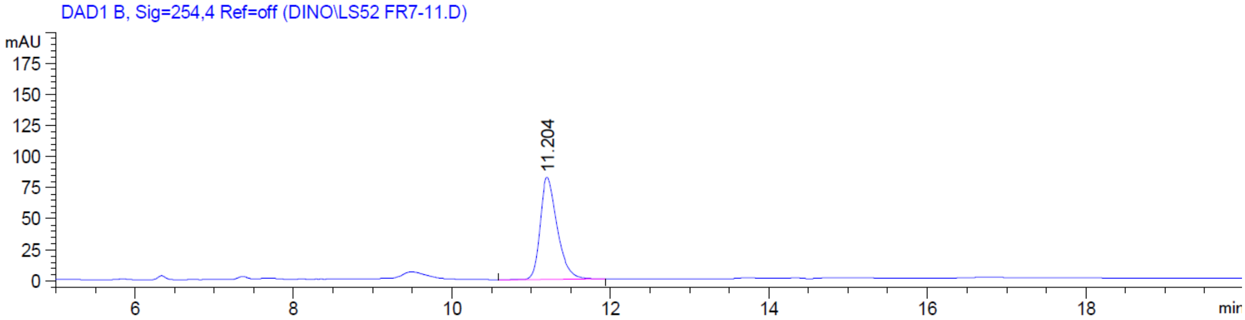

Signal 2: DAD1 B, Sig=254,4 Ref=off

Peak #	RetTime [min]	Type	Width [min]	Area [mAU*s]	Height [mAU]	Area %
1	7.793	BV	0.1541	1603.99707	158.46017	52.7206
2	11.645	BB	0.2471	1438.45020	87.83121	47.2794

Signal 2: DAD1 B, Sig=254,4 Ref=off

Peak #	RetTime [min]	Type	Width [min]	Area [mAU*s]	Height [mAU]	Area %
1	11.204	BB	0.2259	1224.39868	82.13548	100.0000

4,4-dimethyl-2-((R)-2-pentyl-1-((R)-1-phenylethyl)azetidin-2-yl)-4,5-dihydrooxazole 2e

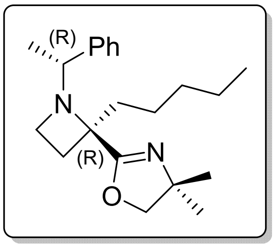

Following GP1 with iodopentane as an electrophile, compound **2e** was obtained as pale-yellow oil (56 mg, 90%). dr = 90:10, er > 99:1 (LUX-1, 99:1 Hex:iPrOH + 0.5 % DEA, 0.5mL/min). $[\alpha]^{20}_{D}$ = + 28.66° (c = 1, $CHCl_3$). R_f = 0.6 (hexane/AcOEt 60:40). **FT-IR** (film, cm^{-1}) 2961, 2927, 2857, 1697, 1648, 1491, 1453, 1364, 1276, 1190, 1118, 973, 761. **^{1}H NMR** (500 MHz, $CDCl_3$) δ 7.30 (d, *J* = 7.1 Hz, 2H, Ar-H), 7.27 – 7.22 (m, 2H, Ar-H), 7.21 – 7.16 (m, 1H, Ar-H), 3.92 (s, 2H, OCH_2), 3.57 (q, *J* = 6.5 Hz, 1H, C*H*CH_3), 3.28 – 3.19 (m, 2H, NCH_2), 2.42 (ddd, *J* = 10.6, 7.9, 4.3 Hz, 1H, NCH_2CH_2), 2.02 (dt, *J* = 10.4, 8.0 Hz, 1H, NCH_2CH_2), 1.65 – 1.55 (m, 1H, pentyl-CH_2), 1.51 – 1.43 (m, 1H, pentyl-CH_2), 1.36 (s, 3H, oxazoline-CH_3), 1.29 (s, 3H, oxazoline-CH_3), 1.26 – 1.11 (m, 5H, pentyl-CH_2), 1.16 (d, *J* = 6.5 Hz, 3H, CHC*H*$_3$) 1.10 – 1.01 (m, 1H, pentyl-CH_2), 0.83 (t, *J* = 7.1 Hz, 3H, pentyl-CH_3). **^{13}C NMR** (126 MHz, $CDCl_3$) δ 166.3 (C=N), 145.6 (Ar-C_q), 127.9 (2 x Ar-C), 127.6 (2 x Ar-C), 126.9 (Ar-C_q), 78.6 (OCH_2), 68.3 (C_q), 67.2 (C_q), 61.1 ($CHCH_3$), 48.6 (NCH_2), 36.4 (pentyl-CH_2), 32.2 (pentyl-CH_2), 29.1 (oxazoline-CH_3), 28.6 (oxazoline-CH_3), 26.3 (NCH_2CH_2), 23.6 (pentyl-CH_2), 22.8 (pentyl-CH_2), 22.6 ($CHCH_3$), 14.1 (pentyl-CH_3). **HRMS** calculated for $C_{21}H_{33}N_2O$ $[M+H]^+$ 329.2593; found 329.2583.

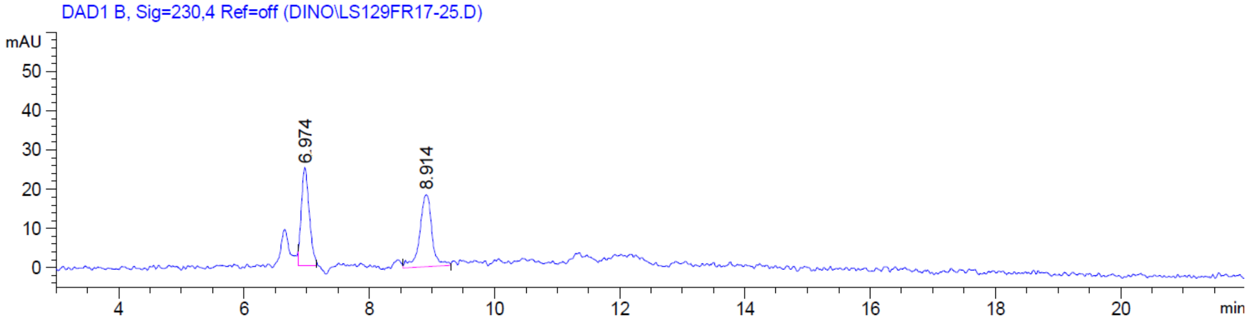

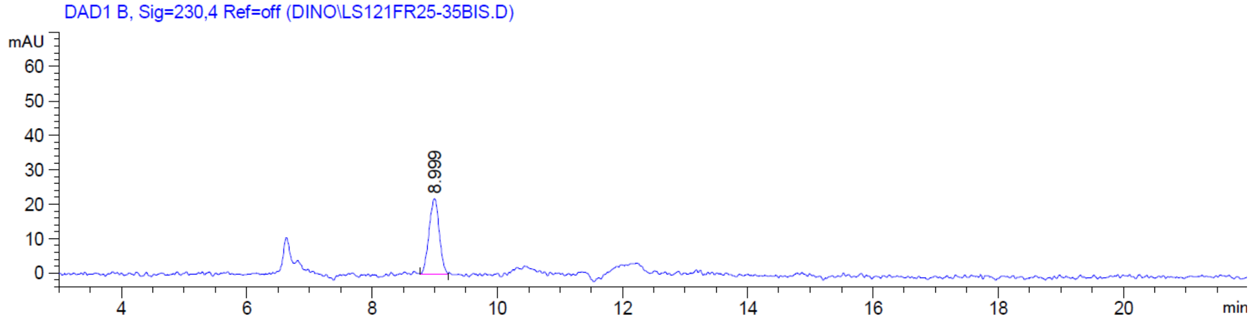

Signal 2: DAD1 B, Sig=230,4 Ref=off

Peak #	RetTime [min]	Type	Width [min]	Area [mAU*s]	Height [mAU]	Area %
1	6.974	MM	0.1457	218.01645	24.93319	46.4994
2	8.914	VV	0.2074	250.84247	18.36023	53.5006

Signal 2: DAD1 B, Sig=230,4 Ref=off

Peak #	RetTime [min]	Type	Width [min]	Area [mAU*s]	Height [mAU]	Area %
1	8.999	VV	0.1778	253.92418	22.14774	100.0000

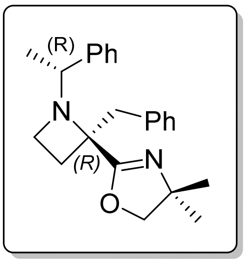

2-((R)-2-benzyl-1-((R)-1-phenylethyl)azetidin-2-yl)-4,4-dimethyl-4,5-dihydrooxazole 2f

Following GP1 with benzylbromide as an electrophile, compound **2f** was obtained as pale-yellow oil (60 mg, 90%). dr = 90:10, er > 99:1 (LUX-1, 99.5:0.5 Hex:iPrOH + 0.2% DEA, 0.8mL/min). $[\alpha]^{20}_{D}$ = + 78.41° (c = 1, $CHCl_3$). R_f = 0.4 (hexane/AcOEt 70:30). **FT-IR** (film, cm^{-1}) 3294, 2965, 2927, 2853, 1731, 1649, 1494, 1452, 1188, 1084, 974, 762, 700. **^{1}H NMR** (500 MHz, $CDCl_3$) δ 7.39 (d, *J* = 7.3 Hz, 2H, Ar-H), 7.30 (t, *J* = 7.5 Hz, 2H, Ar-H), 7.24 – 7.21 (m, 1H, Ar-H), 7.19 – 7.08 (m, 5H, Ar-H), 3.95 (d, *J* = 8.1 Hz, 1H, OCH_2), 3.89 (d, *J* = 8.1 Hz, 1H, OCH_2), 3.61 (q, *J* = 6.5 Hz, 1H, C*H*CH_3), 3.37 – 3.32 (m, 1H, NCH_2), 3.30 – 3.24 (m, 1H, NCH_2), 2.99 (d, *J* = 13.5 Hz, 1H, CH_2Ph), 2.86 (d, *J* = 13.5 Hz, 1H, CH_2Ph), 2.37 – 2.31 (m, 1H, NCH_2CH_2), 2.21 (dt, *J* = 10.5, 8.2 Hz, 1H, NCH_2CH_2), 1.37 (s, 3H, oxazoline-CH_3), 1.21 (d, *J* = 6.5 Hz, 3H, CHCH_3), 1.14 (s, 3H, oxazoline-CH_3). **^{13}C NMR** (126 MHz, $CDCl_3$) δ 165.1 (C=N), 145.8 (Ar-C_q), 137.3 (Ar-C_q), 130.0 (2 x Ar-C), 128.0 (2 x Ar-C), 128.0 (2 x Ar-C), 127.6 (2 x Ar-C), 127.0 (Ar-C), 126.2 (Ar-C), 78.6 (OCH_2), 69.0 (C_q), 67.4 (C_q) 61.2 ($CHCH_3$), 48.6 (NCH_2), 43.5 (CH_2Ph), 29.3 (oxazoline-CH_3), 28.5 (oxazoline-CH_3), 26.6 (NCH_2CH_2), 22.5 ($CHCH_3$). **HRMS** calculated for $C_{23}H_{28}N_2NaO$ $[M+Na]^+$ 371.2099; found 371.2074.

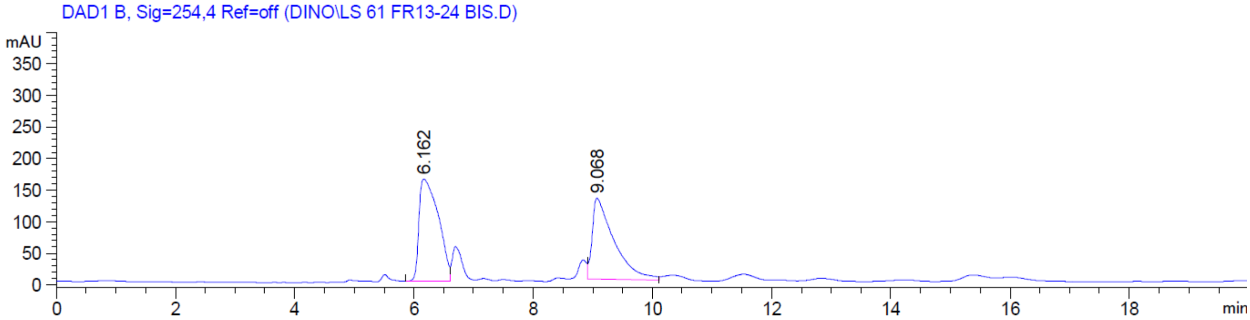

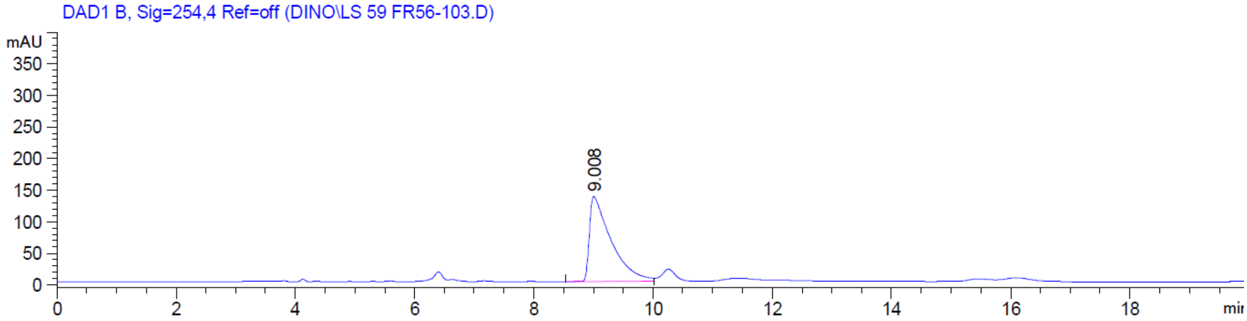

Signal 2: DAD1 B, Sig=254,4 Ref=off

Peak #	RetTime [min]	Type	Width [min]	Area [mAU*s]	Height [mAU]	Area %
1	6.162	BV	0.3884	3642.67114	161.97476	52.5127
2	9.068	VV	0.3576	3294.07397	128.26949	47.4873

Signal 2: DAD1 B, Sig=254,4 Ref=off

Peak #	RetTime [min]	Type	Width [min]	Area [mAU*s]	Height [mAU]	Area %
1	9.008	BV	0.3524	3415.50342	134.41577	100.0000

2-((R)-2-(3-fluoropropyl)-1-((R)-1-phenylethyl)azetidin-2-yl)-4,4-dimethyl-4,5-dihydrooxazole 2g

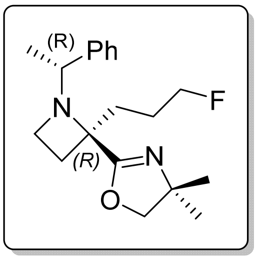

Following GP1 with 1-fluoro-3-iodopropane as an electrophile, compound **2g** was obtained as pale-yellow oil (54 mg, 90%). dr = 90:10, er > 99:1 (LUX-1, 99.5:0.5 Hex:iPrOH + 0.2% DEA, 0.5mL/min). $[\alpha]^{20}_{D}$ = + 76.20° (c = 1, $CHCl_3$). R_f = 0.4 (hexane/AcOEt 70:30). **FT-IR** (film, cm^{-1}) 3583, 2966, 2927, 2851, 1648, 1451, 1365, 1266, 1189, 993, 763, 741, 702. **^{1}H NMR** (500 MHz, $CDCl_3$) δ 7.33 – 7.20 (m, 5H, Ar-H), 4.44 – 4.18 (m, 2H, CH_2F), 3.96 (s, 2H, OCH_2), 3.59 (q, *J* = 6.5 Hz, 1H, $CHCH_3$), 3.35 – 3.26 (m, 2H, NCH_2), 2.37 (ddd, *J* = 10.6, 7.1, 4.9 Hz, 1H, NCH_2CH_2), 2.09 – 1.99 (m, 1H, NCH_2CH_2), 1.77 – 1.46 (m, 4H, $CH_2CH_2CH_2F$), 1.33 (s, 3H, oxazoline-CH_3), 1.30 (s, 3H, oxazoline-CH_3), 1.15 (d, *J* = 6.5 Hz, 3H, $CHCH_3$). **^{13}C NMR** (126 MHz, $CDCl_3$) δ 166.1 (C=N), 145.3 (Ar-C_q), 128.0 (2 x Ar-C), 127.6 (2 x Ar-C), 127.1 (Ar-C), 84.3 (d, $^1J_{C\text{-}F}$ = 164.9 Hz, CH_2F), 78.7 (OCH_2), 67.7 (C_q), 67.3 (C_q), 61.0 ($CHCH_3$), 48.5 (NCH_2), 31.9 (d, $^3J_{C\text{-}F}$ = 5.9 Hz, $CH_2CH_2CH_2F$), 29.0 (oxazoline-CH_3), 28.5 (oxazoline-CH_3), 25.9 (NCH_2CH_2), 25.2 (d, $^2J_{C\text{-}F}$ = 19.8 Hz, $CH_2CH_2CH_2F$), 22.3 ($CHCH_3$). **^{19}F NMR** (470 MHz, $CDCl_3$) δ −216.52–−216.81 (m, 1F). **HRMS** calculated for $C_{19}H_{27}FN_2NaO$ $[M+Na]^+$ 341.2005; found 341.2006.

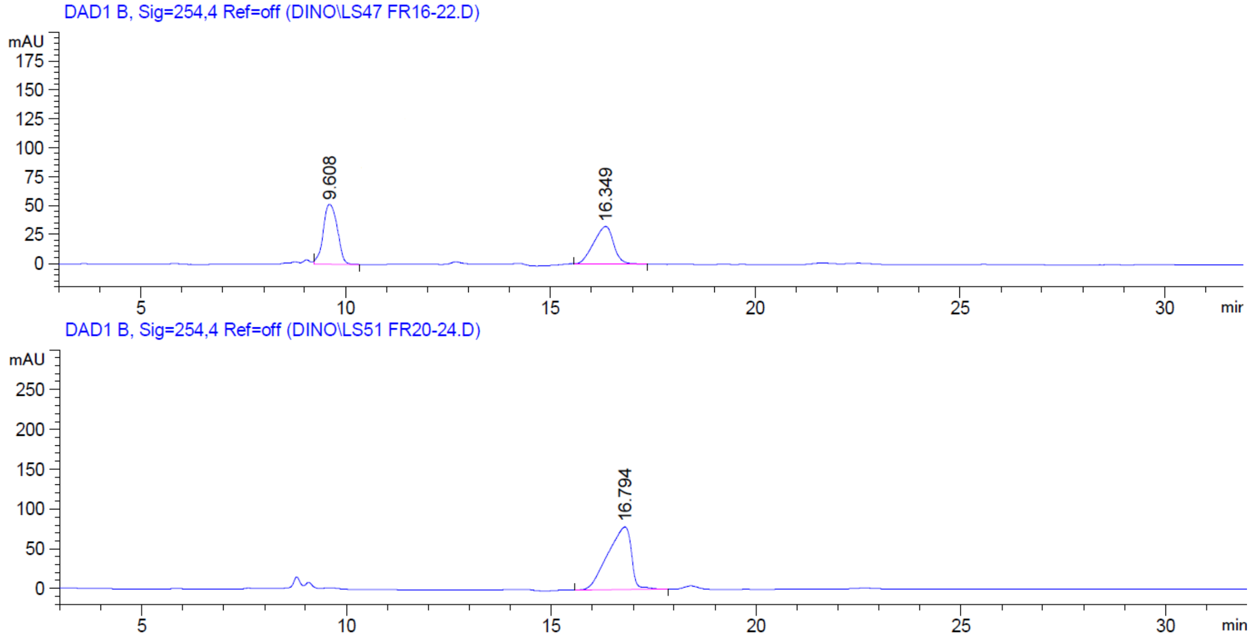

Signal 2: DAD1 B, Sig=254,4 Ref=off

Peak #	RetTime [min]	Type	Width [min]	Area [mAU*s]	Height [mAU]	Area %
1	9.608	FM	0.3972	1233.76831	51.77532	52.9300
2	16.349	BB	0.5245	1097.17700	32.44685	47.0700

Signal 2: DAD1 B, Sig=254,4 Ref=off

Peak #	RetTime [min]	Type	Width [min]	Area [mAU*s]	Height [mAU]	Area %
1	16.794	BB	0.5652	3152.02612	78.70773	100.0000

(R)-tert-butyl 2-(4,4-dimethyl-4,5-dihydrooxazol-2-yl)-1-((R)-1-phenylethyl)azetidine-2-carboxylate 2h

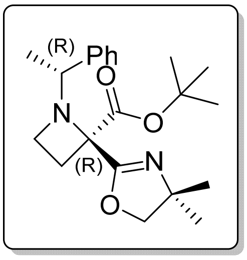

Following GP1 with di-*tert*-butyl dicarbonate as an electrophile, compound **2h** was obtained as colourless oil (64 mg, 94%). dr = 90:10, er > 99:1 (LUX-1, 99:1 Hex:iPrOH + 0.5% DEA, 0.3mL/min). $[\alpha]^{20}_{D}$ = + 31.80° (c = 1, $CHCl_3$). R_f = 0.3 (hexane/AcOEt 70:30). **FT-IR** (film, cm^{-1}) 2973, 2930, 2874, 1726, 1659, 1445, 1367, 1269, 1163, 1103, 976, 844, 763, 701. **^{1}H NMR** (500 MHz, $CDCl_3$) δ 7.37 – 7.34 (m, 2H, Ar-H), 7.29 – 7.25 (m, 2H, Ar-H), 7.22 – 7.18 (m, 1H, Ar-H), 4.01 – 3.97 (m, 2H, OCH_2), 3.94 (q, *J* = 6.6 Hz, 1H, C*H*CH_3), 3.20 – 3.14 (m, 1H, NCH_2), 3.07 – 3.00 (m, 1H, NCH_2), 2.69 – 2.61 (m, 1H, NCH_2CH_2), 2.45 (ddd, *J* = 10.9, 8.5, 4.0 Hz, 1H, NCH_2CH_2), 1.50 (s, 9H, $C(CH_3)_3$), 1.36 (s, 3H, oxazoline-CH_3), 1.30 (s, 3H, oxazoline-CH_3), 1.28 (d, *J* = 6.6 Hz, 3H, CHC*H*$_3$). **^{13}C NMR** (126 MHz, $CDCl_3$) δ 169.3 (C=O), 165.1 (C=N), 143.9 (Ar-C_q), 128.2 (2 x Ar-C), 127.8 (2 x Ar-C), 127.0 (Ar-C), 82.2 (C_q), 79.7 (OCH_2), 69.7 (C_q), 67.1 (C_q), 61.8 ($CHCH_3$), 49.1 (NCH_2), 28.22 (oxazoline-CH_3), 28.20 (oxazoline-CH_3), 28.16 ($C(CH_3)_3$), 26.9 (NCH_2CH_2), 22.3 ($CHCH_3$). **HRMS** calculated for $C_{21}H_{31}N_2O_3$ $[M+H]^+$ 359.2335; found 359.2328.

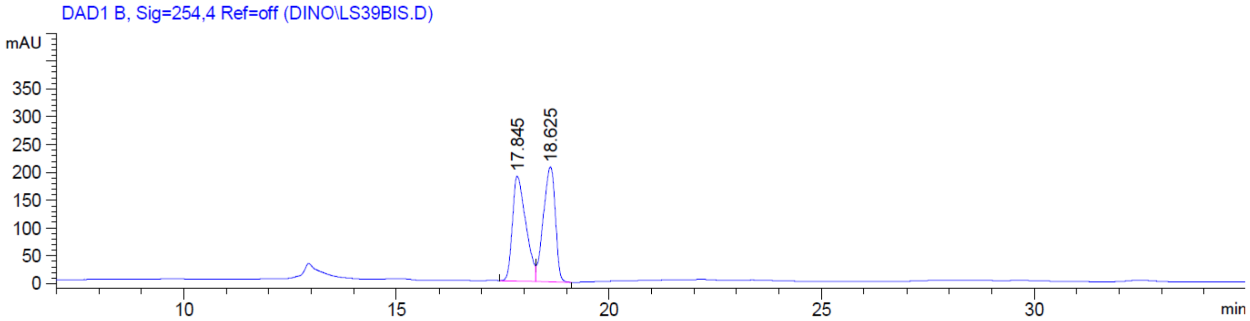

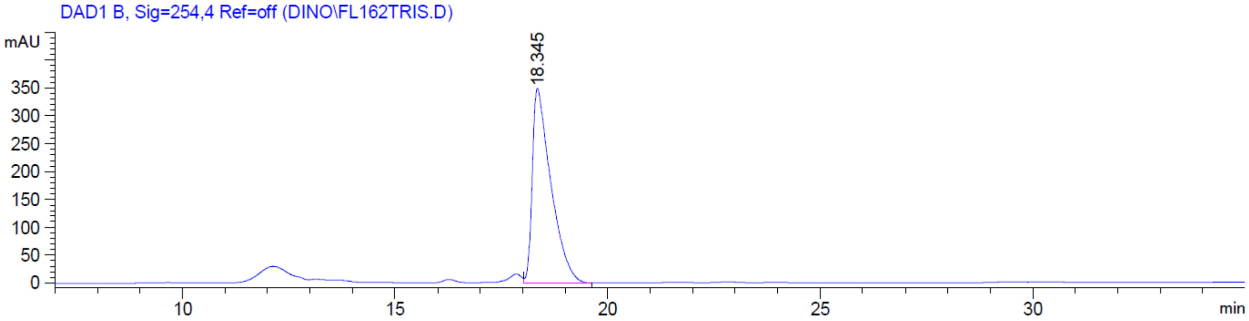

Signal 2: DAD1 B, Sig=254,4 Ref=off

Peak #	RetTime [min]	Type	Width [min]	Area [mAU*s]	Height [mAU]	Area %
1	17.845	BV	0.3260	4132.50684	188.35155	50.5898
2	18.625	VB	0.3264	4036.14746	206.19432	49.4102

Signal 2: DAD1 B, Sig=254,4 Ref=off

Peak #	RetTime [min]	Type	Width [min]	Area [mAU*s]	Height [mAU]	Area %
1	18.345	VB	0.4358	1.05819e4	348.94339	100.0000

2-((R)-2-(4,4-dimethyl-4,5-dihydrooxazol-2-yl)-1-((R)-1-phenylethyl)azetidin-2-yl)propan-2-ol major-2i

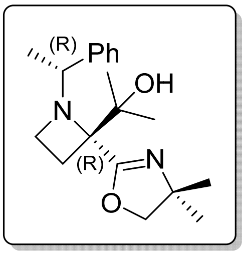

Following GP1 with acetone as an electrophile, compound **major-2i** was obtained as a white solid (36 mg, 60%). mp 128-130 °C, dr = 65:35, er > 99:1 (AD-H, 99.5:0.5 Hex:iPrOH + 0.2% DEA, 0.5mL/min). $[\alpha]^{20}_{D}$ = + 73.35° (c = 0.67, $CHCl_3$). R_f = 0.6 (hexane/AcOEt 70:30). **FT-IR** (KBr, cm^{-1}) 3370, 2971, 2969, 2956, 1638, 1450, 1373, 1236, 1175, 1074, 970, 770, 702. **^{1}H NMR** (500 MHz, $CDCl_3$) δ 7.32 – 7.26 (m, 4H, Ar-H), 7.23 – 7.18 (m, 1H, Ar-H), 5.75 (bs, OH), 4.08 (d, *J* = 8.1 Hz, 1H, OCH_2), 4.01 (d, *J* = 8.1 Hz, 1H, OCH_2), 3.53 (q, *J* = 6.5 Hz, 1H, $CHCH_3$), 2.90 – 2.79 (m, 2H, NCH_2), 2.21 (ddd, *J* = 11.2, 8.7, 3.1 Hz, 1H, NCH_2CH_2), 2.09 – 1.99 (m, 1H, NCH_2CH_2), 1.49 (s, 3H, $C(CH_3)_2OH$), 1.38 (s, 3H, oxazoline-CH_3), 1.32 (s, 3H, oxazoline-CH_3), 1.29 (d, *J* = 6.5 Hz, 3H, $CHCH_3$), 1.22 (s, 3H, $C(CH_3)_2OH$). **^{13}C NMR** (126 MHz, $CDCl_3$) δ 165.7 (C=N), 144.9 (Ar-C_q), 128.3 (2 x Ar-C), 127.7 (2 x Ar-C), 126.9 (Ar-C), 77.7 (OCH_2), 73.5 (C_q), 72.5 (C_q), 67.6 (C_q), 62.6 ($CHCH_3$), 48.0 (NCH_2), 28.9 (oxazoline-CH_3), 28.5 (oxazoline-CH_3), 26.0 ($C(CH_3)_2OH$), 24.4 ($CHCH_3$), 24.3 ($C(CH_3)_2OH$), 23.8 (NCH_2CH_2). **HRMS** calculated for $C_{19}H_{29}N_2O_2$ $[M+H]^+$ 317.2229; found 317.2238.

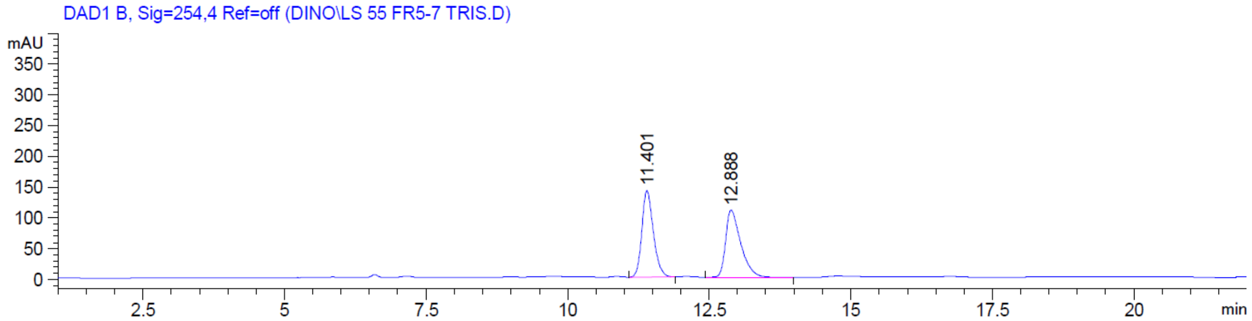

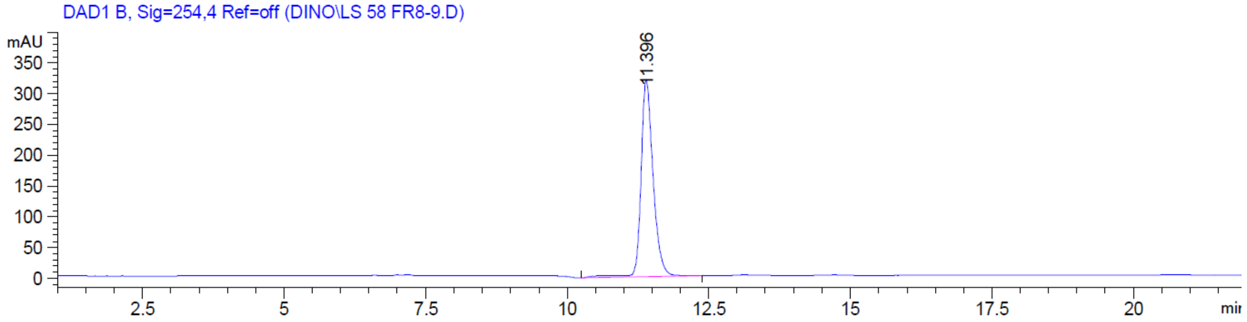

Signal 2: DAD1 B, Sig=254,4 Ref=off

Peak #	RetTime [min]	Type	Width [min]	Area [mAU*s]	Height [mAU]	Area %
1	11.401	BB	0.2145	1984.28027	140.69478	49.5694
2	12.888	BB	0.2747	2018.75195	109.68359	50.4306

Signal 2: DAD1 B, Sig=254,4 Ref=off

Peak #	RetTime [min]	Type	Width [min]	Area [mAU*s]	Height [mAU]	Area %
1	11.396	BB	0.2215	4767.08984	320.47934	100.0000

2-((S)-2-(4,4-dimethyl-4,5-dihydrooxazol-2-yl)-1-((R)-1-phenylethyl)azetidin-2-yl)propan-2-ol minor-2i

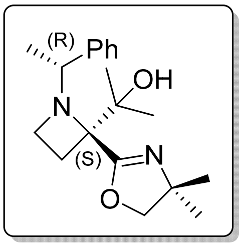

Following GP1 with acetone as an electrophile, compound **minor-2i** was obtained as a white solid (18 mg, 30%). mp 145-147 °C, dr = 35:65, er > 99:1 (AD-H, 99:1 Hex:iPrOH, 0.5mL/min). $[\alpha]^{20}_{D}$ = + 42.25° (c = 0.47, $CHCl_3$). R_f = 0.4 (hexane/AcOEt 70:30). **FT-IR** (KBr, cm^{-1}) 3328, 2977, 2970, 2958, 1680, 1452, 1380, 1235, 1180, 1070, 980, 765, 700. **^{1}H NMR** (500 MHz, $CDCl_3$) δ 7.31 – 7.25 (m, 4H, Ar-H), 7.21 – 7.16 (m, 1H, Ar-H), 4.07 – 4.00 (q, *J* = 6.8 Hz, 1H *C*HCH$_3$ and s, 2H, OCH$_2$), 3.55 – 3.48 (m, 1H, NCH$_2$), 3.28 – 3.23 (m, 1H, NCH$_2$), 2.39 – 2.27 (m, 2H, NCH$_2$*C*H$_2$), 1.35 (s, 3H, oxazoline-CH$_3$), 1.34 (s, 3H, oxazoline-CH$_3$), 1.26 (d, *J* = 6.8 Hz, 3H, CHC*H*$_3$), 1.12 (s, 3H,C(C*H*$_3$)$_2$OH), 0.84 (s, 3H, C(C*H*$_3$)$_2$OH). **^{13}C NMR** (126 MHz, $CDCl_3$) δ 165.2 (C=N), 146.5 (Ar-C$_q$), 128.2 (2 x Ar-C), 127.3 (2 x Ar-C), 126.7 (Ar-C), 78.4 (OCH$_2$), 74.8 (C$_q$), 72.3 (C$_q$), 67.3 (C$_q$), 58.5 (CHCH$_3$), 45.3 (NCH$_2$), 28.6 (oxazoline-CH$_3$), 28.5 (oxazoline-CH$_3$), 25.8 (C(CH$_3$)$_2$OH), 23.9 (C(CH$_3$)$_2$OH), 23.87 (NCH$_2$CH$_2$), 19.8 (CHCH$_3$). **HRMS** calculated for $C_{19}H_{29}N_2O_2$ $[M+H]^+$ 317.2229; found 317.2240.

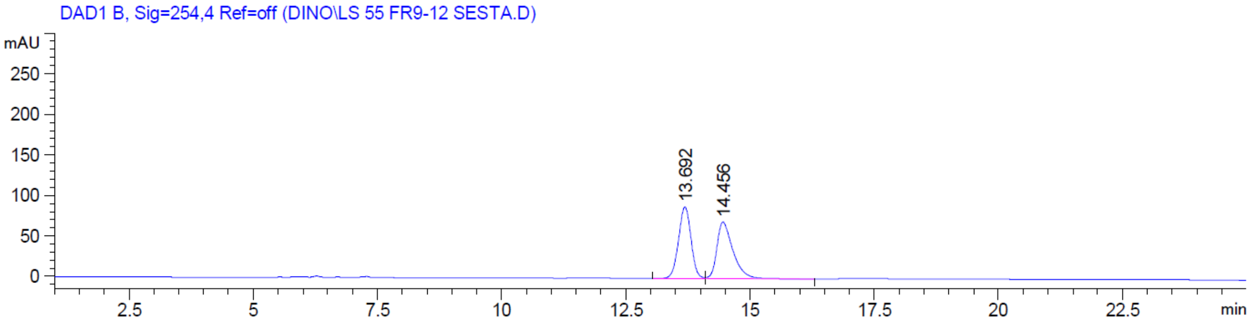

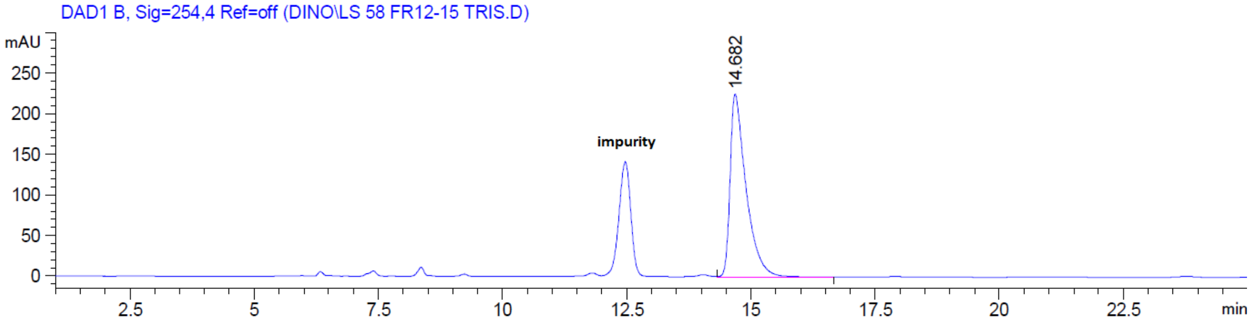

```
Signal 2: DAD1 B, Sig=254,4 Ref=off

Peak RetTime Type  Width     Area      Height     Area
  #   [min]        [min]   [mAU*s]     [mAU]        %
----|-------|----|-------|----------|----------|--------|
   1  13.692 BV    0.2731 1556.32837   88.54645  49.6251
   2  14.456 VB    0.3371 1579.84204   70.05061  50.3749

Signal 2: DAD1 B, Sig=254,4 Ref=off

Peak RetTime Type  Width     Area      Height     Area
  #   [min]        [min]   [mAU*s]     [mAU]        %
----|-------|----|-------|----------|----------|--------|
   1  14.682 VB    0.3242 5019.31494  225.07886 100.0000
```

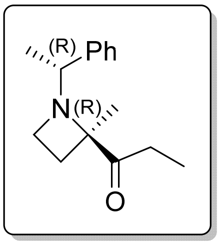

1-((R)-2-methyl-1-((R)-1-phenylethyl)azetidin-2-yl)propan-1-one 3a

Following GP2 with ethyllithium (0.5 M in benzene/cyclohexane), compound **3a** was obtained as a yellow oil (16 mg, 50%). dr 99:1, $[\alpha]^{20}_{D}$ = + 104.96° (c = 0.5, $CHCl_3$). **FT-IR** (film, cm^{-1}) 2971, 2931, 2849 1704, 1644, 149, 1453, 1368, 1221, 1094, 1028, 765, 700. **^{1}H NMR** (500 MHz, $CDCl_3$) δ 7.35 (d, *J* = 7.4 Hz, 2H, Ar-H), 7.31 (t, *J* = 7.5 Hz, 2H, Ar-H), 7.24 (t, *J* = 7.2 Hz, 1H, Ar-H), 3.63 (q, *J* = 6.3 Hz, 1H, C*H*CH_3), 3.02 (td, *J* = 8.1, 2.8 Hz, 1H, NCH_2), 2.99 – 2.87 (m, 3H, 2H C*H*$_2CH_3$ and 1H NCH_2), 2.23 (dd, *J* = 18.9, 8.6 Hz, 1H, NCH_2C*H*$_2$), 1.63 (ddd, *J* = 10.8, 8.2, 2.8 Hz, 1H, NCH_2C*H*$_2$), 1.47 (s, 3H, C_qCH_3), 1.09 (t, *J* = 7.3 Hz, 3H, CH_2C*H*$_3$), 1.06 (d, *J* = 6.3 Hz, 3H, CHC*H*$_3$). **^{13}C NMR** (126 MHz, $CDCl_3$) δ 215.9 (C=O), 143.5 (Ar-C_q), 128.4 (2 x Ar-C), 127.8 (2 x Ar-C), 127.3 (Ar-C), 71.2 (C_q), 60.8 (CH), 48.3 (NCH_2), 29.8 (CH_2CH_3), 28.5 (NCH_2CH_2), 22.7 ($CHCH_3$), 14.3 (C_qCH_3), 8.2 (CH_2CH_3). **HRMS** calculated for $C_{15}H_{21}NNaO$ $[M+Na]^+$ 254.1521; found 254.1440.

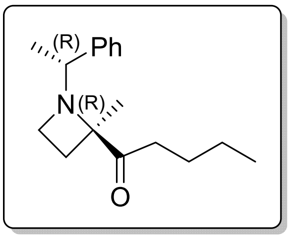

1-((R)-2-methyl-1-((R)-1-phenylethyl)azetidin-2-yl)pentan-1-one 3b

Following GP2 with n-butyllithium (2.5 M in hexane), compound **3b** was obtained as a colourless yellow oil (36 mg; 93%). dr 99:1, $[\alpha]^{20}_{D}$ = + 31.45° (c = 0.4, $CHCl_3$). **FT-IR** (film, cm^{-1}) 2959, 2930, 2871, 1702, 1493, 1454, 1368, 1282, 1222, 1120, 1040, 765, 700. **^{1}H NMR** (500 MHz, $CDCl_3$) δ 7.35 (d, *J* = 7.2 Hz, 2H, Ar-H), 7.31 (t, *J* = 7.5 Hz, 2H, Ar-H), 7.24 (t, *J* = 7.1 Hz, 1H, Ar-H overlapping $CHCl_3$), 3.63 (q, *J* = 6.3 Hz, 1H, C*H*CH_3), 3.05 – 2.99 (m, 1H, NCH_2), 2.99 – 2.82 (m, 3H, 2H $O{=}CCH_2$ and 1H NCH_2), 2.23 (dd, *J* = 18.8, 8.6 Hz, 1H, NCH_2C*H*$_2$), 1.65 – 1.55 (m, 3H, 1H NCH_2C*H*$_2$, 2H $O{=}CCH_2CH_2$ overlapping H_2O signal), 1.46 (s, 3H, C_qCH_3), 1.42 – 1.33 (m, 2H, C*H*$_2CH_3$), 1.06 (d, *J* = 6.3 Hz, 3H, CHC*H*$_3$), 0.95 (t, *J* = 7.3 Hz, 3H, CH_2C*H*$_3$). **^{13}C NMR** (126 MHz, $CDCl_3$) δ 215.3 (C=O), 143.5 (Ar-C_q), 128.4 (2 x Ar-C), 127.8 (2 x Ar-C), 127.3 (Ar-C), 71.3 (C_q), 60.8 (CH), 48.3 (NCH_2), 36.2 ($O{=}CCH_2$), 28.3 (NCH_2CH_2), 26.1 ($O{=}CCH_2CH_2$), 22.73 (CH_2CH_3), 22.70 ($CHCH_3$), 14.2 (CH_2CH_3 and C_qCH_3). **HRMS** calculated for $C_{17}H_{26}NO$ $[M+H]^+$ 260.2014; found 260.2018.

3. 1H, 13C, NOESY NMR SPECTRA FOR ISOLATED COMPOUNDS

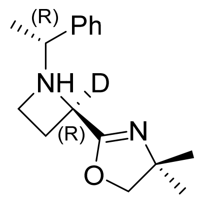

2a

^{1}H NMR

(500 MHz, $CDCl_3$)

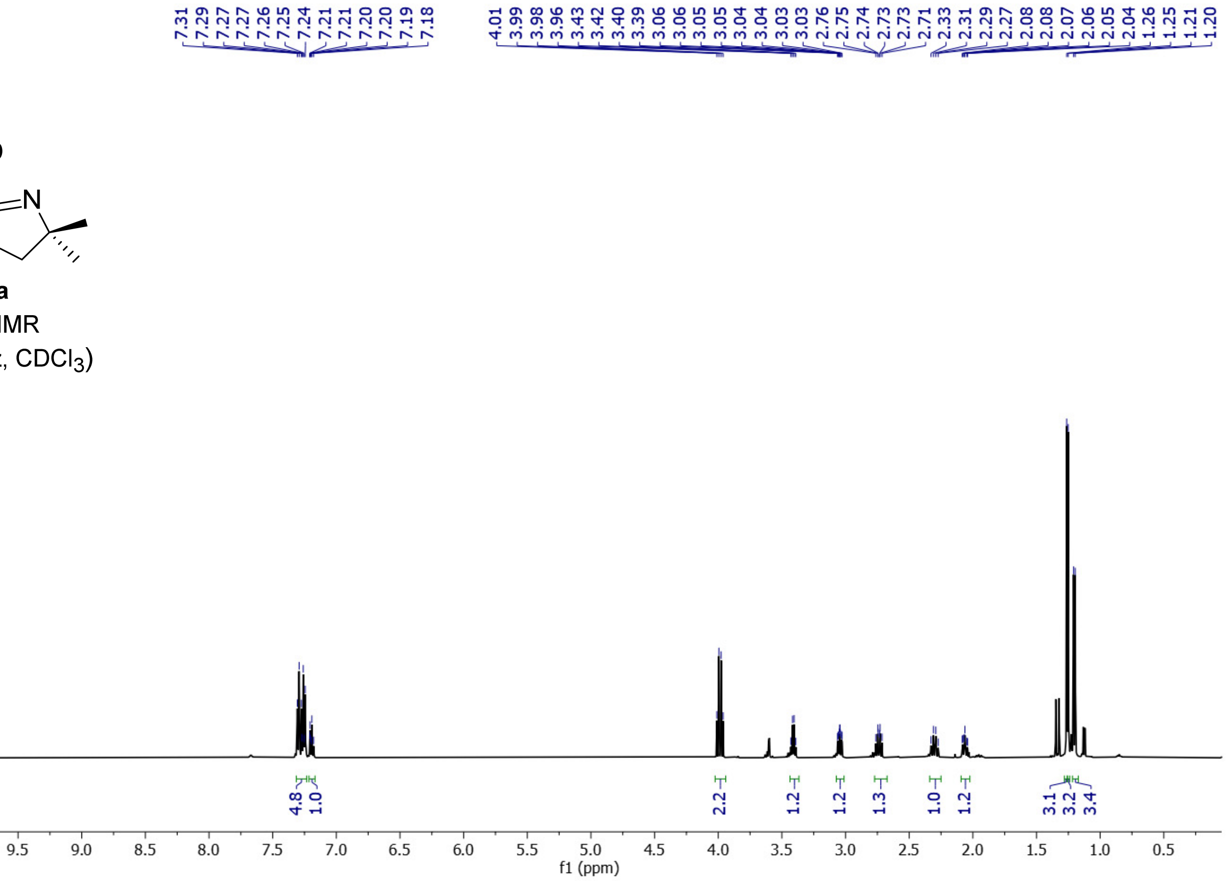

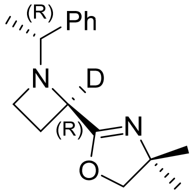

2a
^{13}C NMR
(126 MHz, $CDCl_3$)

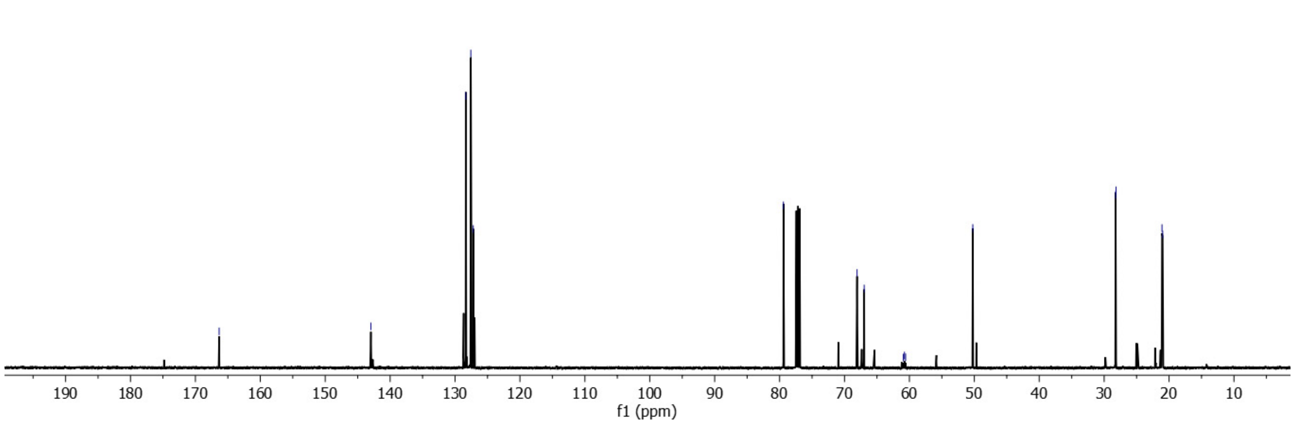

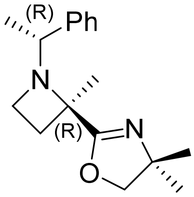

2b
^{1}H NMR
(500 MHz, $CDCl_3$)

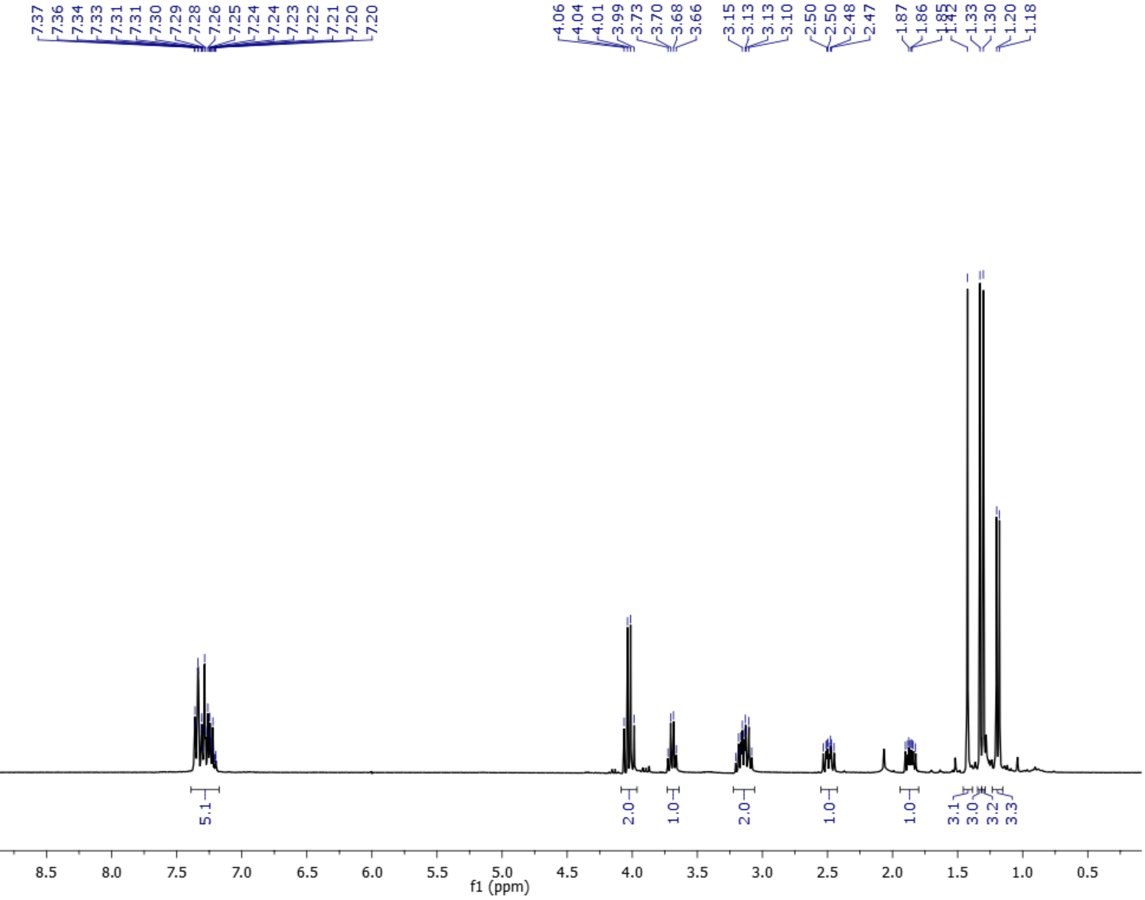

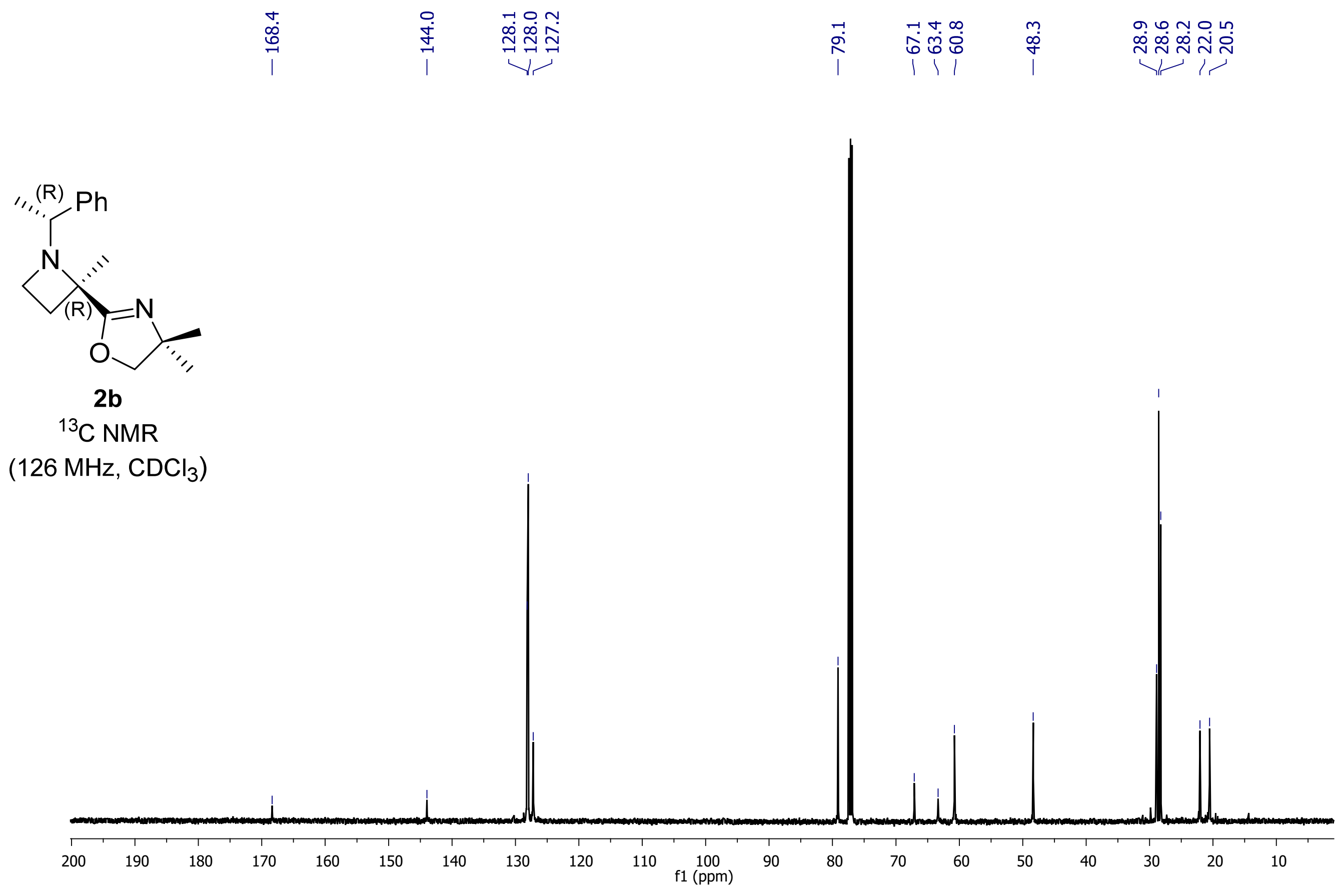
168.4
144.0
128.1
128.0
127.2
79.1
67.1
63.4
60.8
48.3
28.9
28.6
28.2
22.0
20.5
(R)
Ph
N
(R)
N
O
2b
^{13}C NMR
(126 MHz, $CDCl_3$)
f1 (ppm)

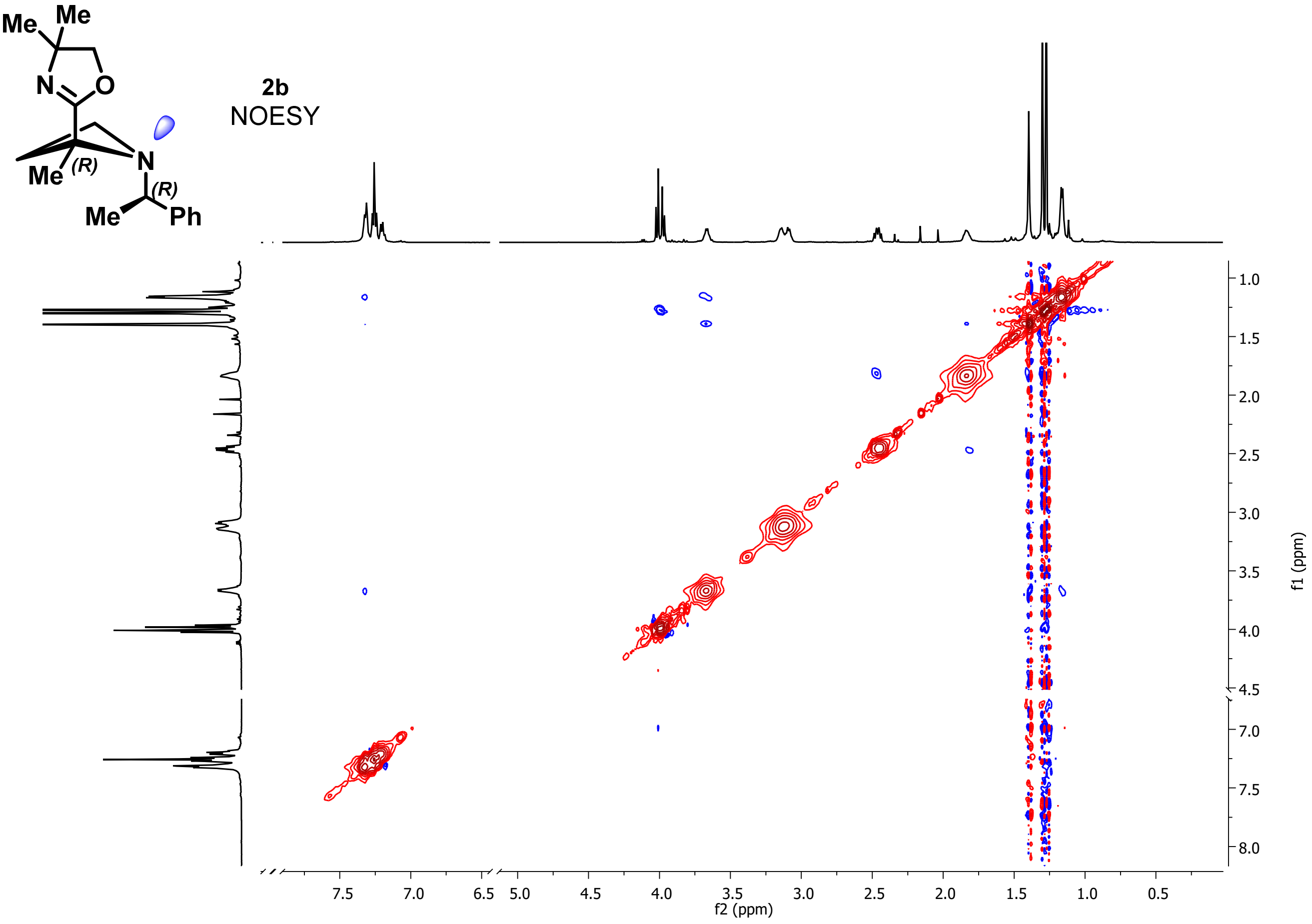
Me
Me
N
O
Me
(R)
N
(R)
Me
Ph
2b
NOESY
f2 (ppm)
f1 (ppm)

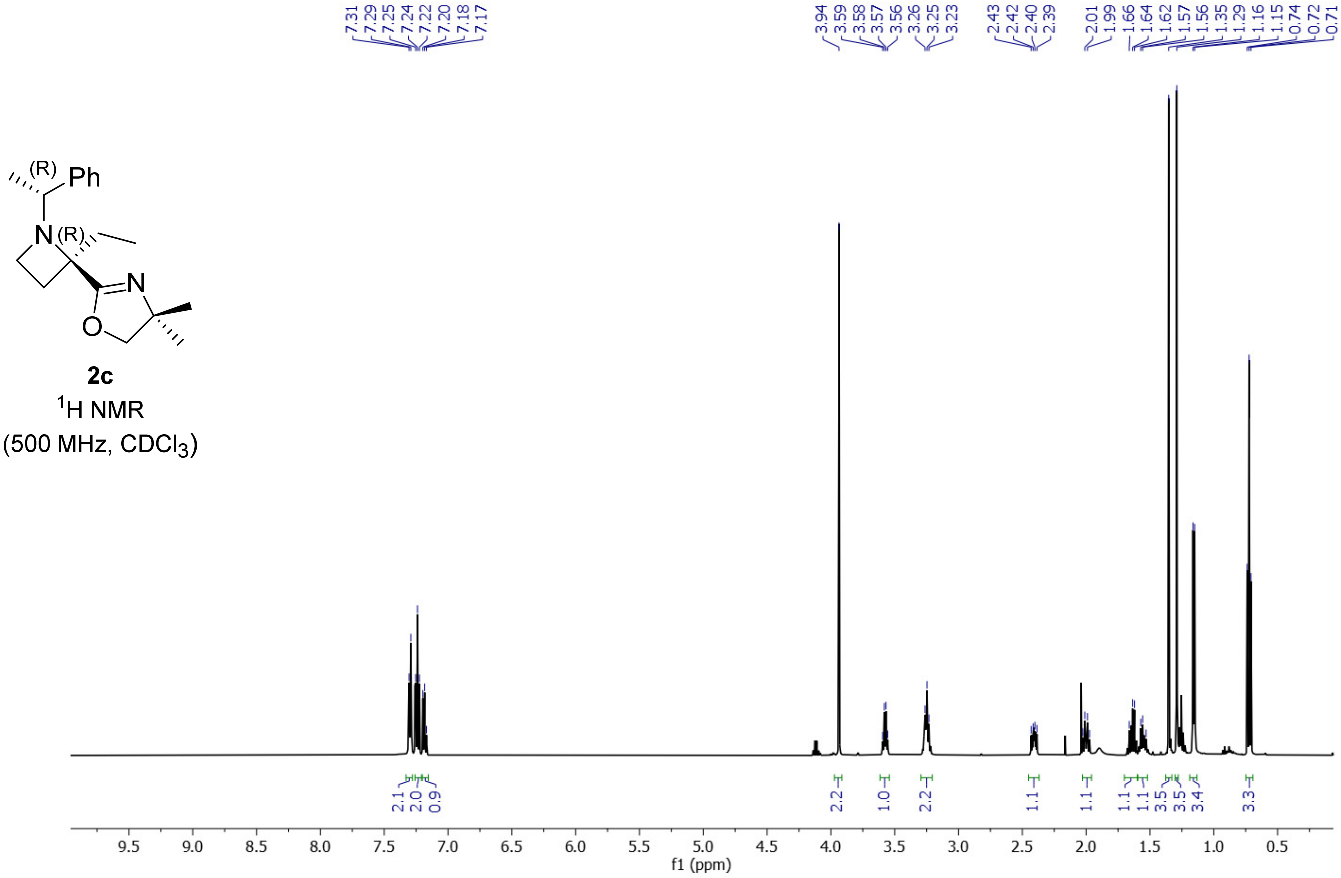

2c
^{1}H NMR
(500 MHz, $CDCl_3$)

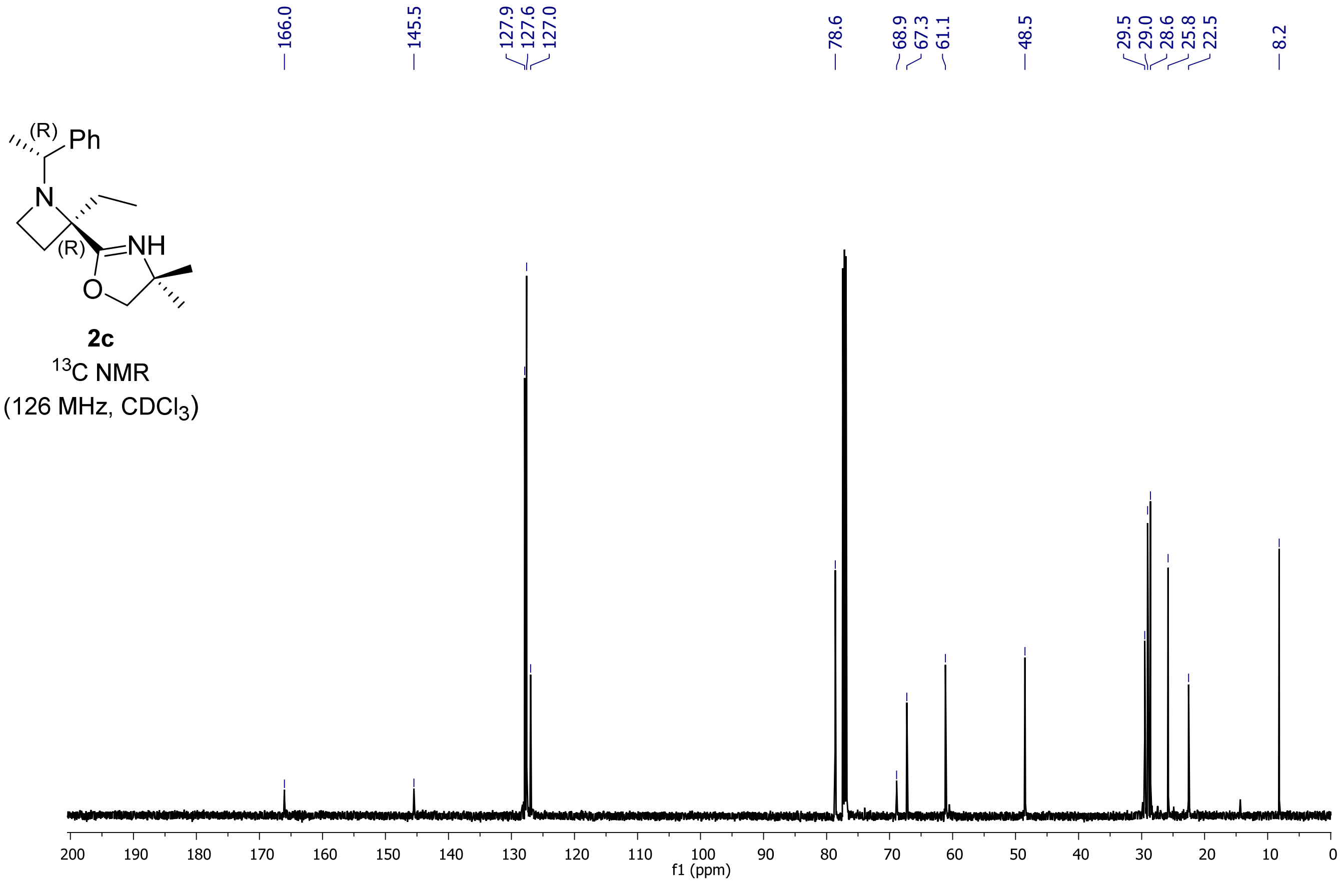

2c
^{13}C NMR
(126 MHz, $CDCl_3$)

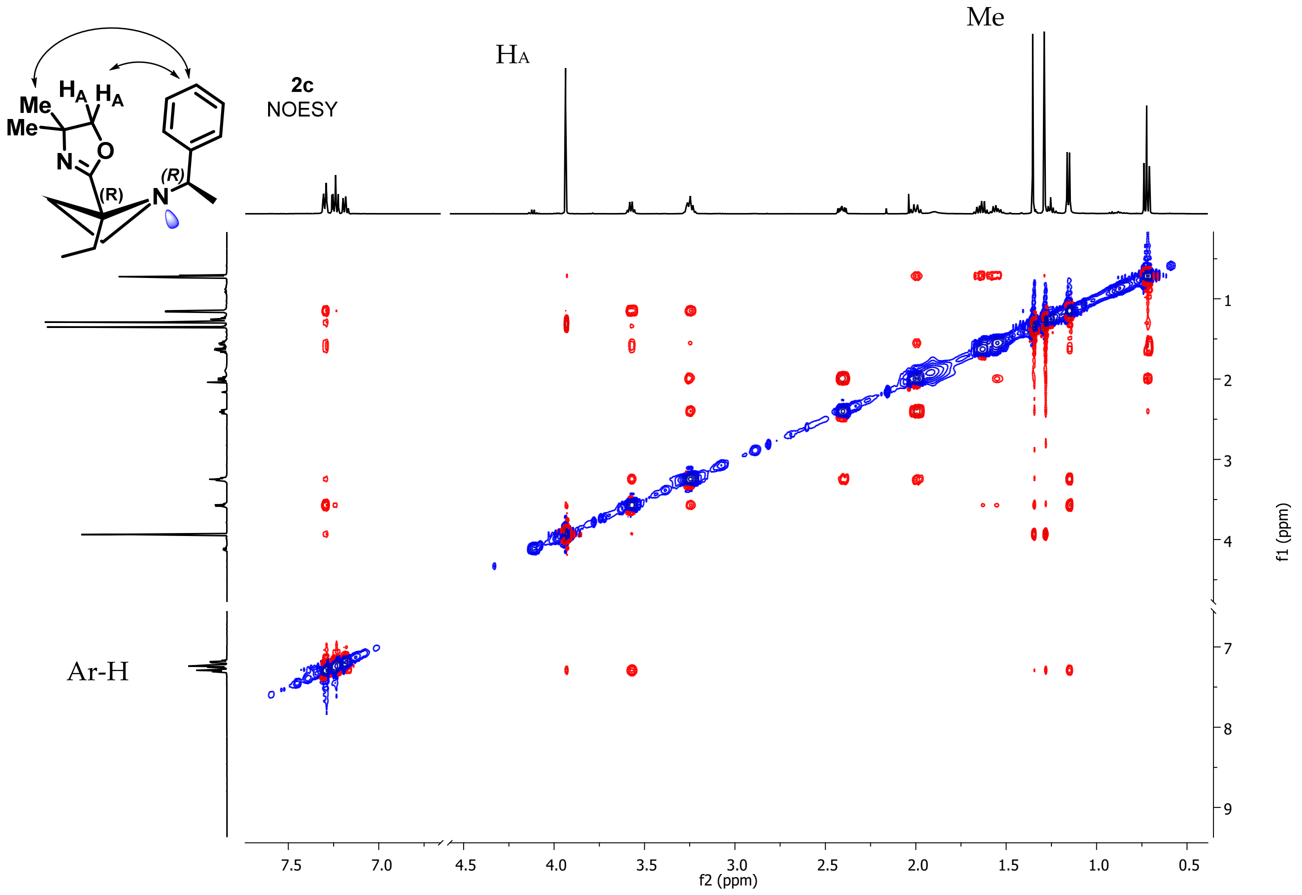
2c
NOESY
HA
Me
Ar-H
f2 (ppm)
f1 (ppm)

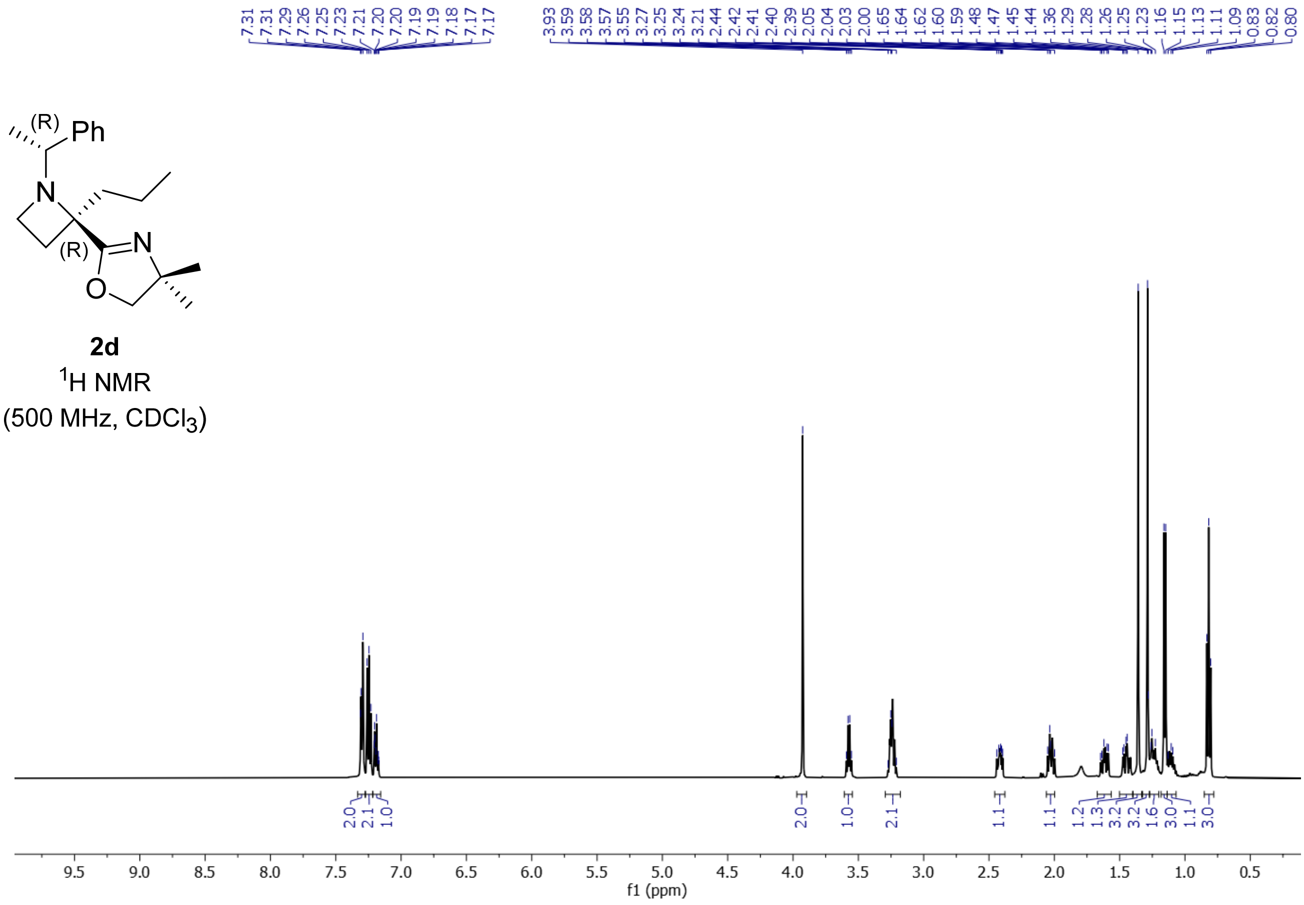
2d
1H NMR
(500 MHz, CDCl3)
f1 (ppm)

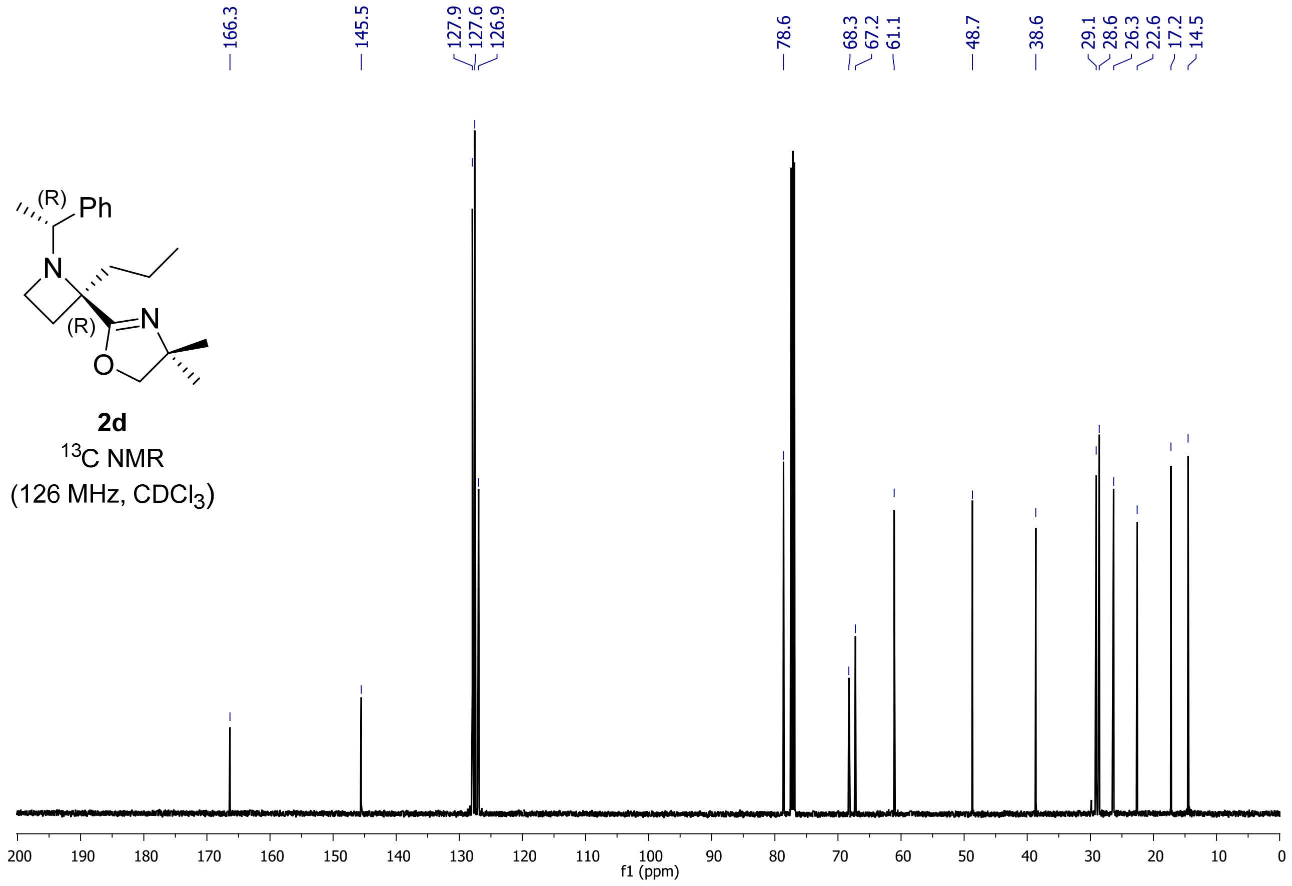

2d
^{13}C NMR
(126 MHz, CDCl$_3$)
f1 (ppm)

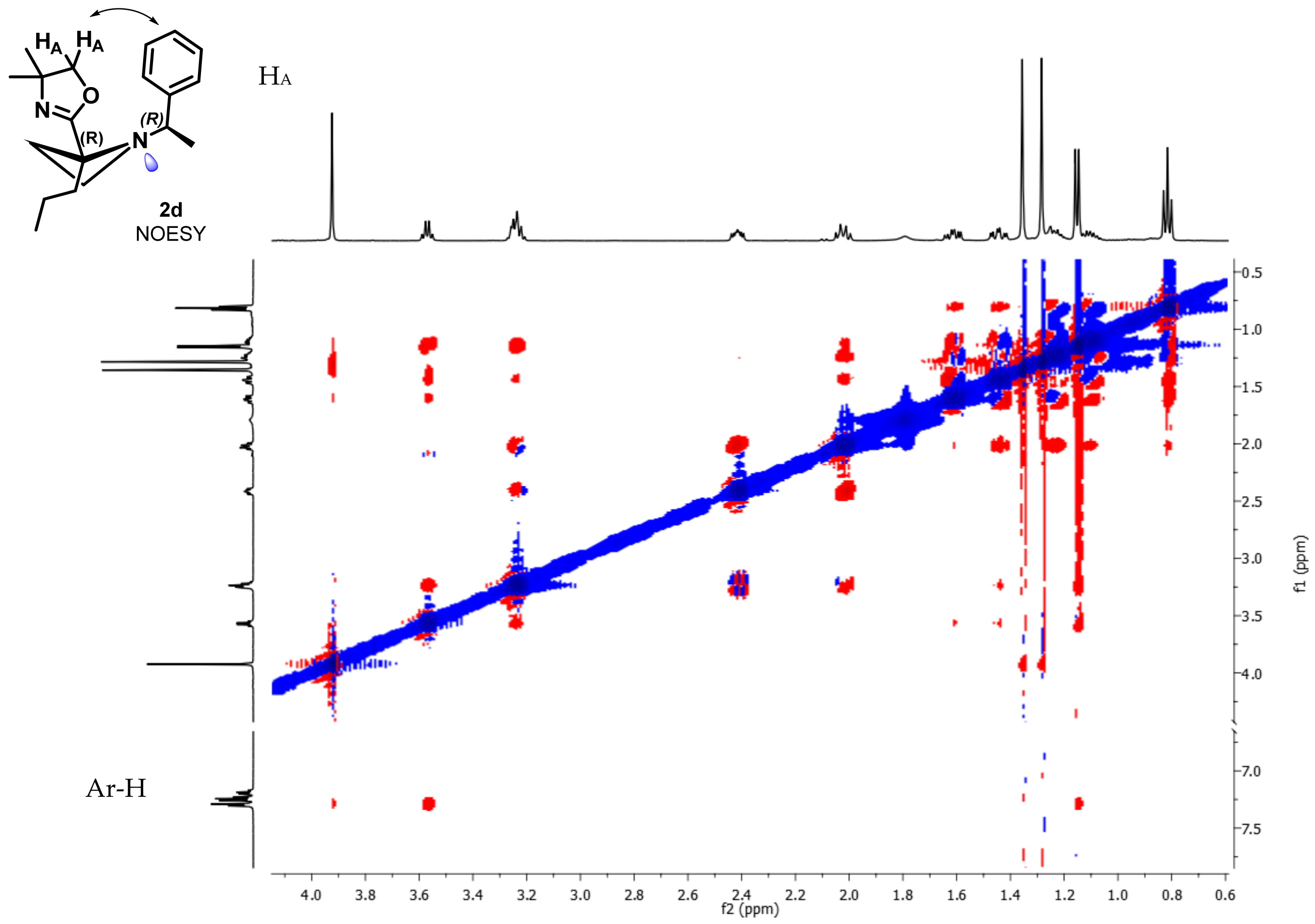

2d
NOESY
HA
Ar-H
f2 (ppm)
f1 (ppm)

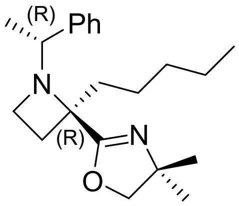

2e
^1H NMR
(500 MHz, $CDCl_3$)

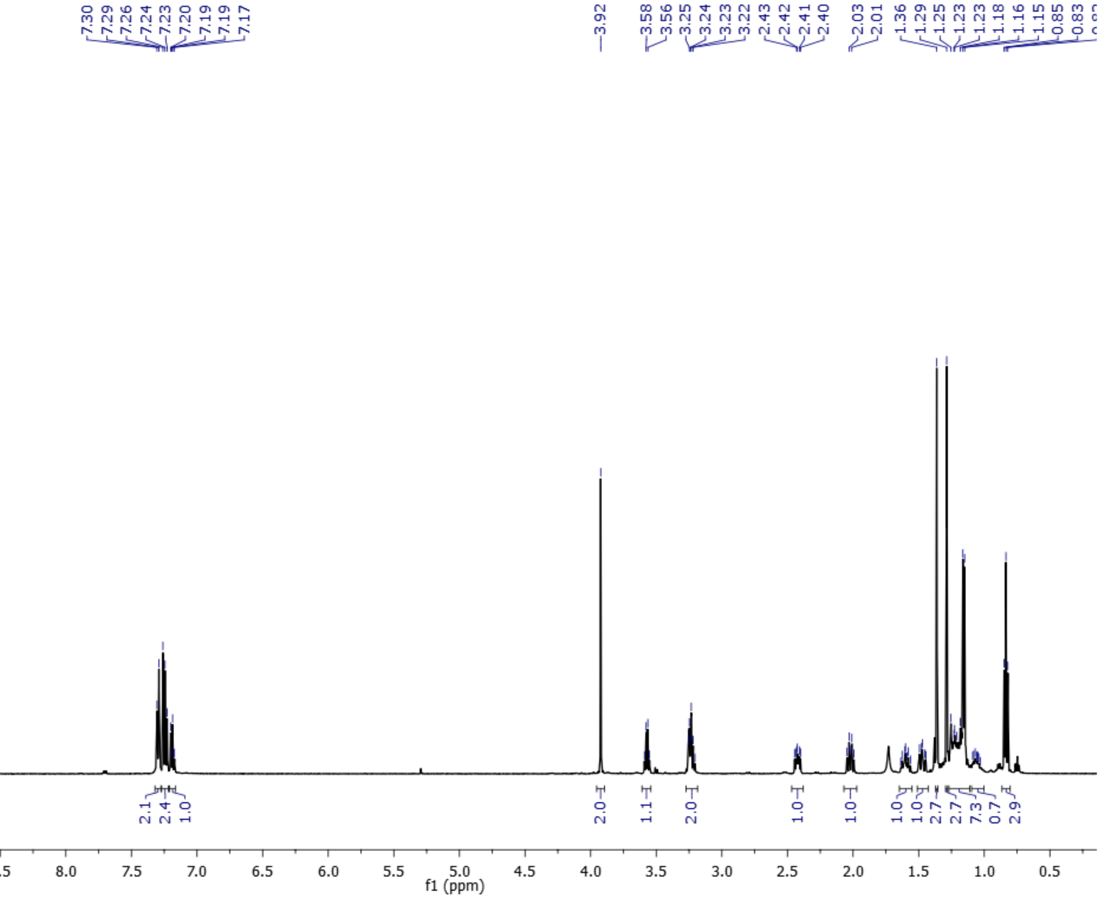

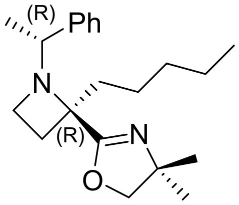

2e
^{13}C NMR
(126 MHz, $CDCl_3$)

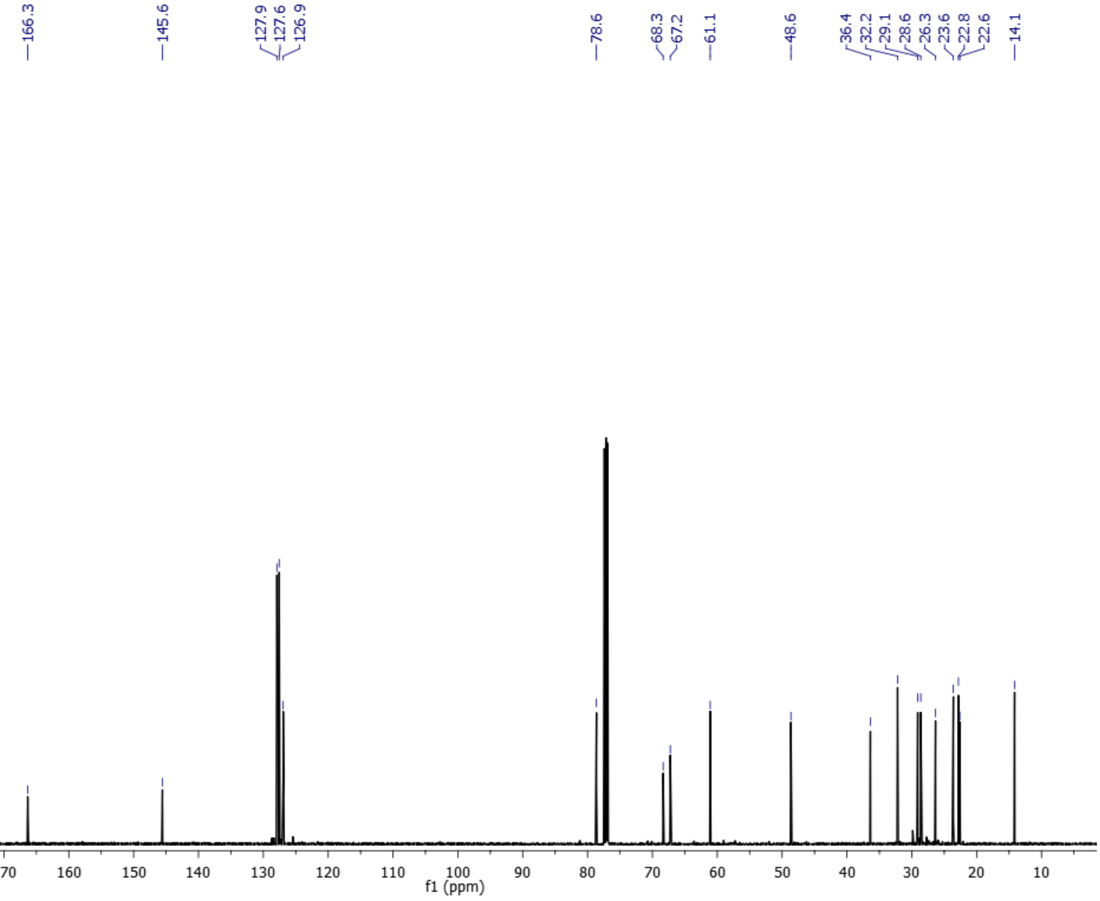

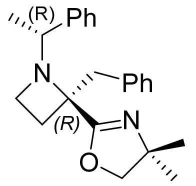

2f
^{1}H NMR
(500 MHz, $CDCl_3$)

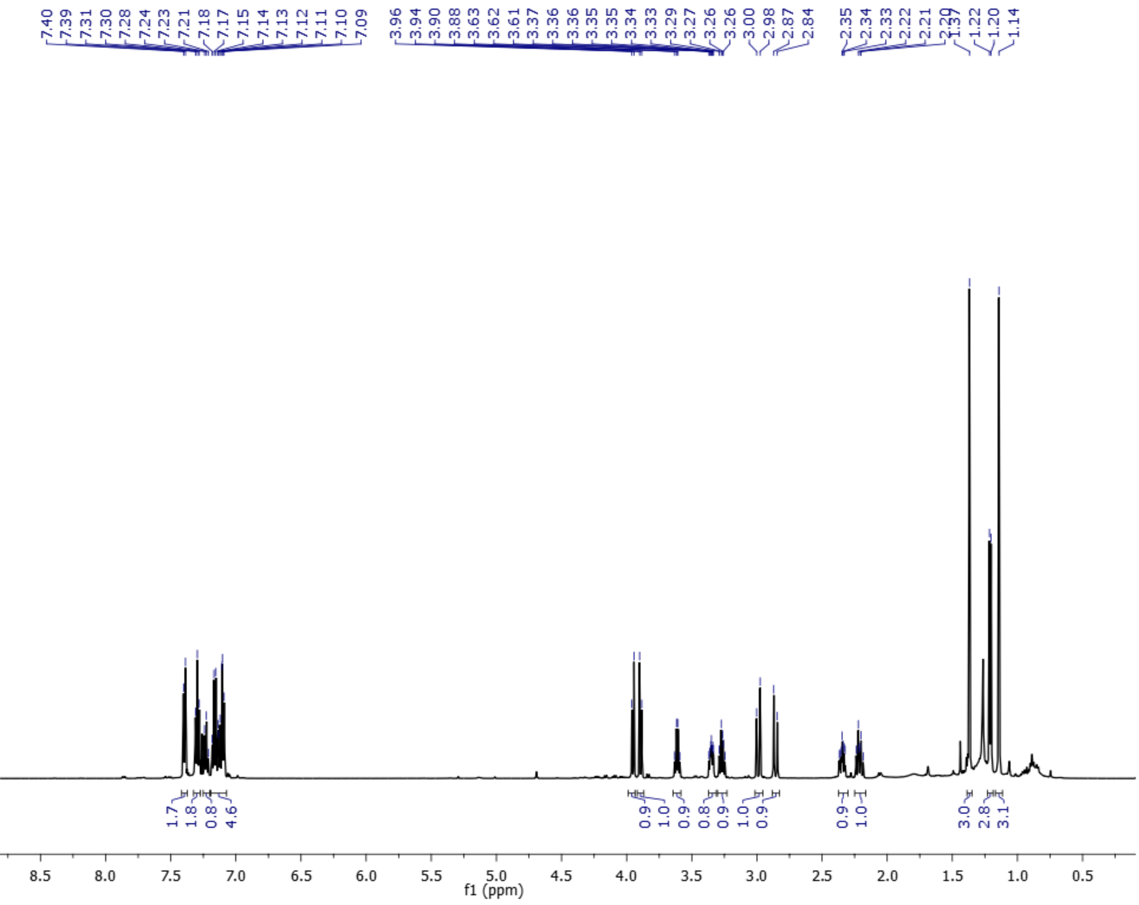

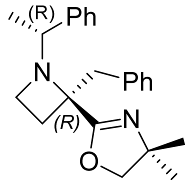

2f
^{13}C NMR
(126 MHz, $CDCl_3$)

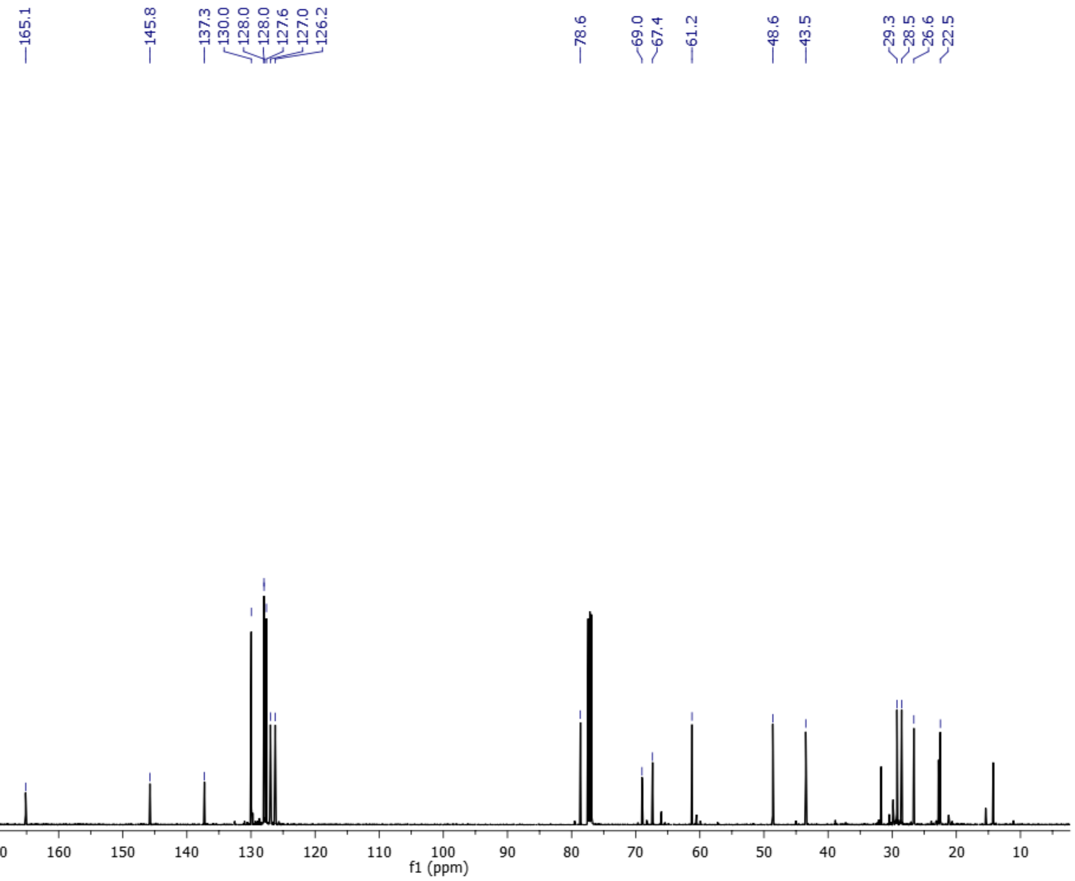

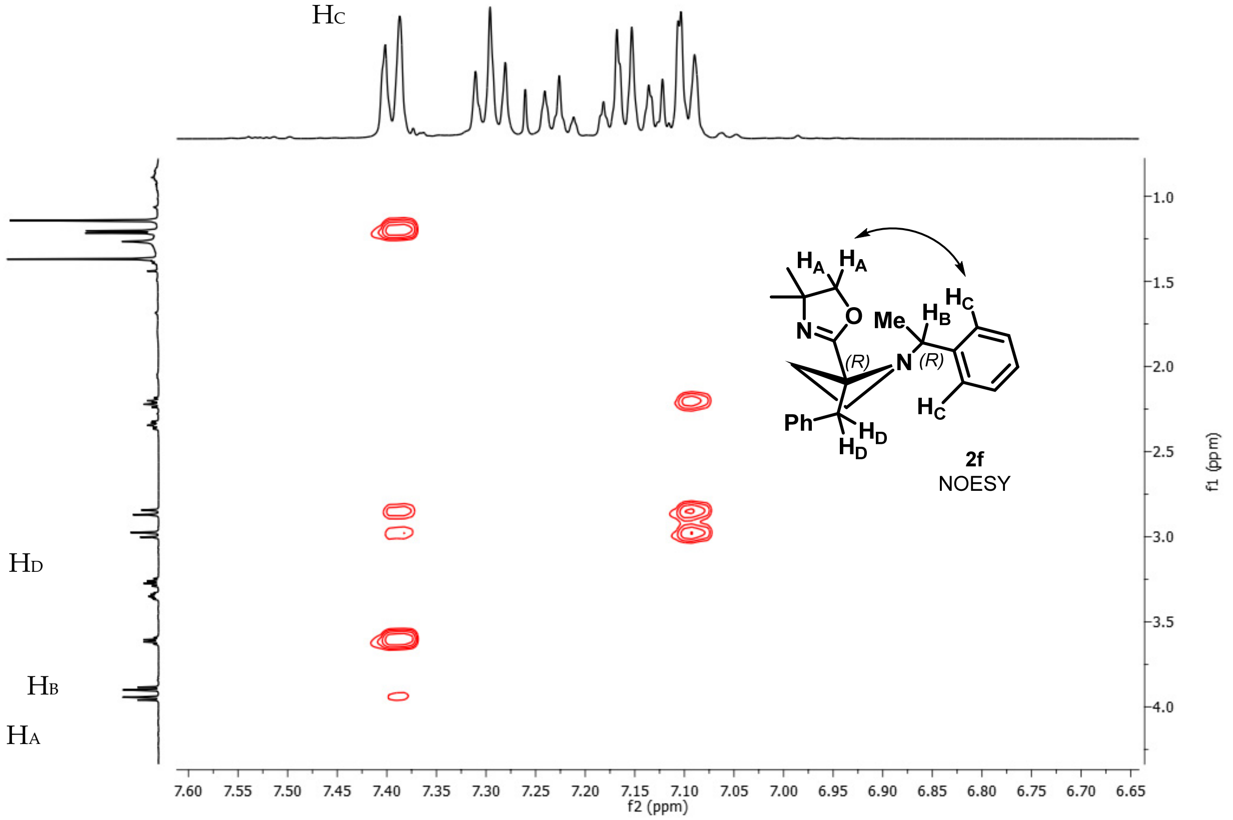
HC
HD
HB
HA
2f
NOESY
f2 (ppm)
f1 (ppm)

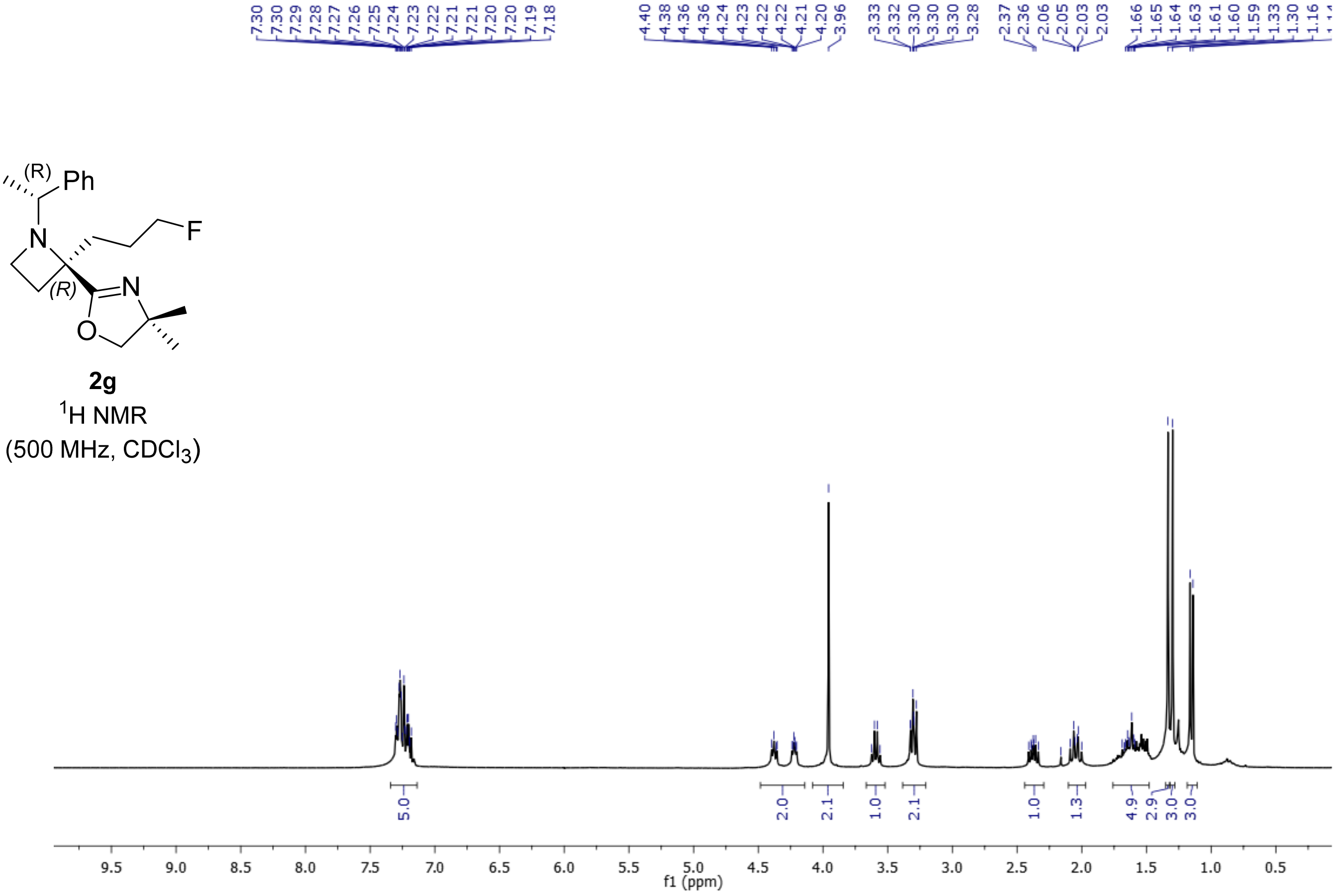
2g
1H NMR
(500 MHz, CDCl3)
f1 (ppm)

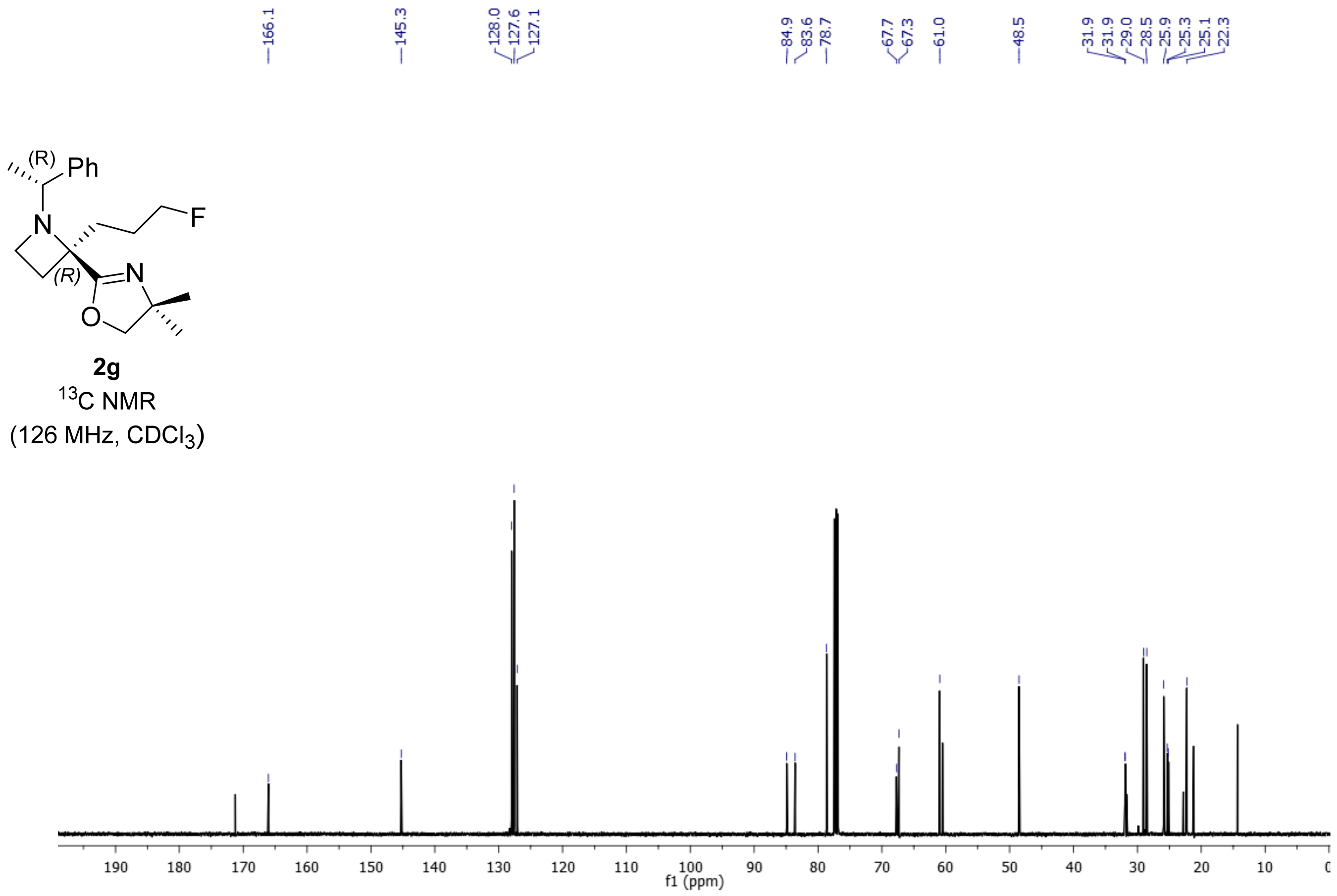

(R)
Ph
N
F
(R)
N
O
2g
^{13}C NMR
(126 MHz, CDCl$_3$)
166.1
145.3
128.0
127.6
127.1
84.9
83.6
78.7
67.7
67.3
61.0
48.5
31.9
31.9
29.0
28.5
25.9
25.3
25.1
22.3
f1 (ppm)

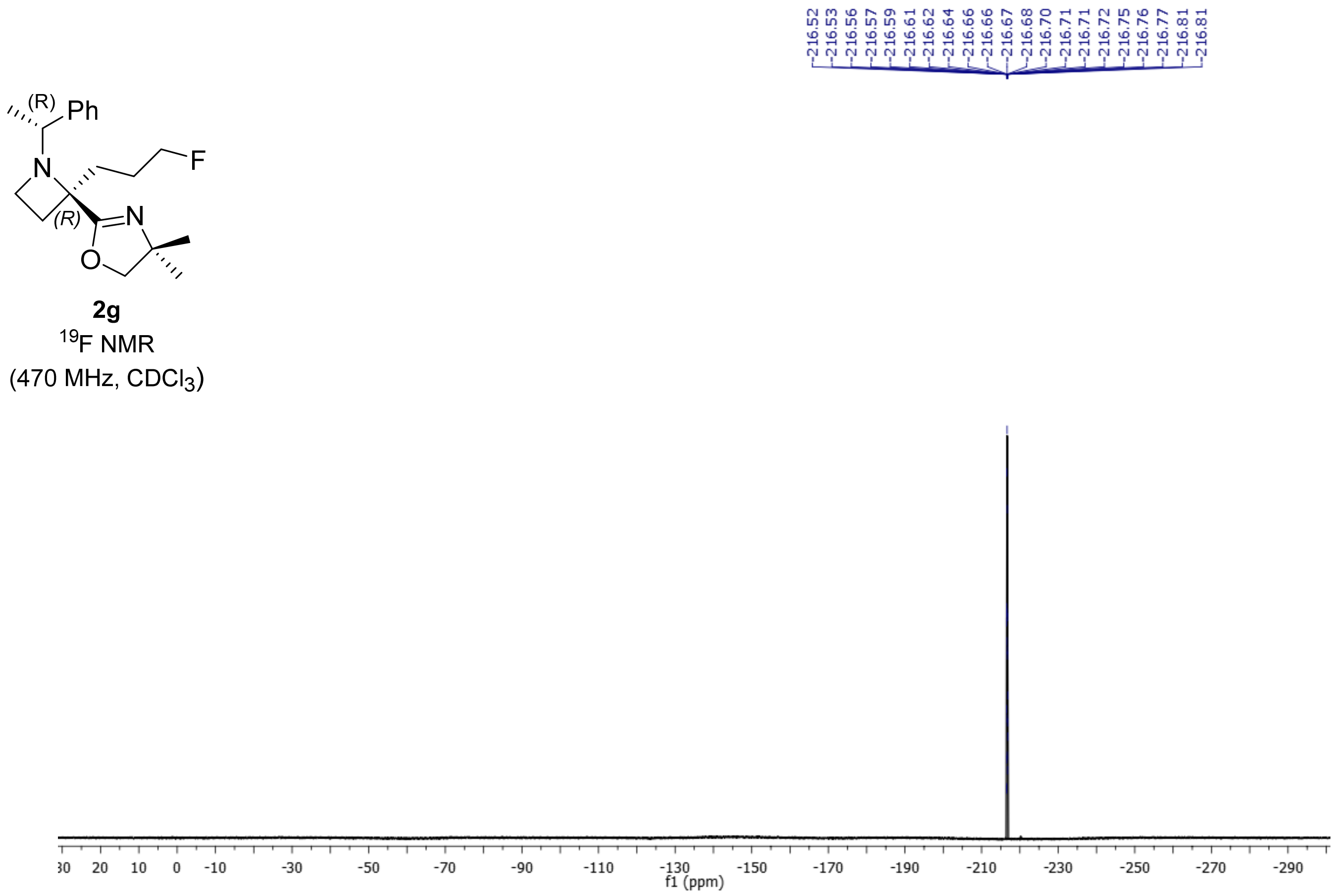

(R)
Ph
N
F
(R)
N
O
2g
^{19}F NMR
(470 MHz, CDCl$_3$)
-216.52
-216.53
-216.56
-216.57
-216.59
-216.61
-216.62
-216.64
-216.66
-216.66
-216.67
-216.68
-216.70
-216.71
-216.71
-216.72
-216.75
-216.76
-216.77
-216.81
-216.81
f1 (ppm)

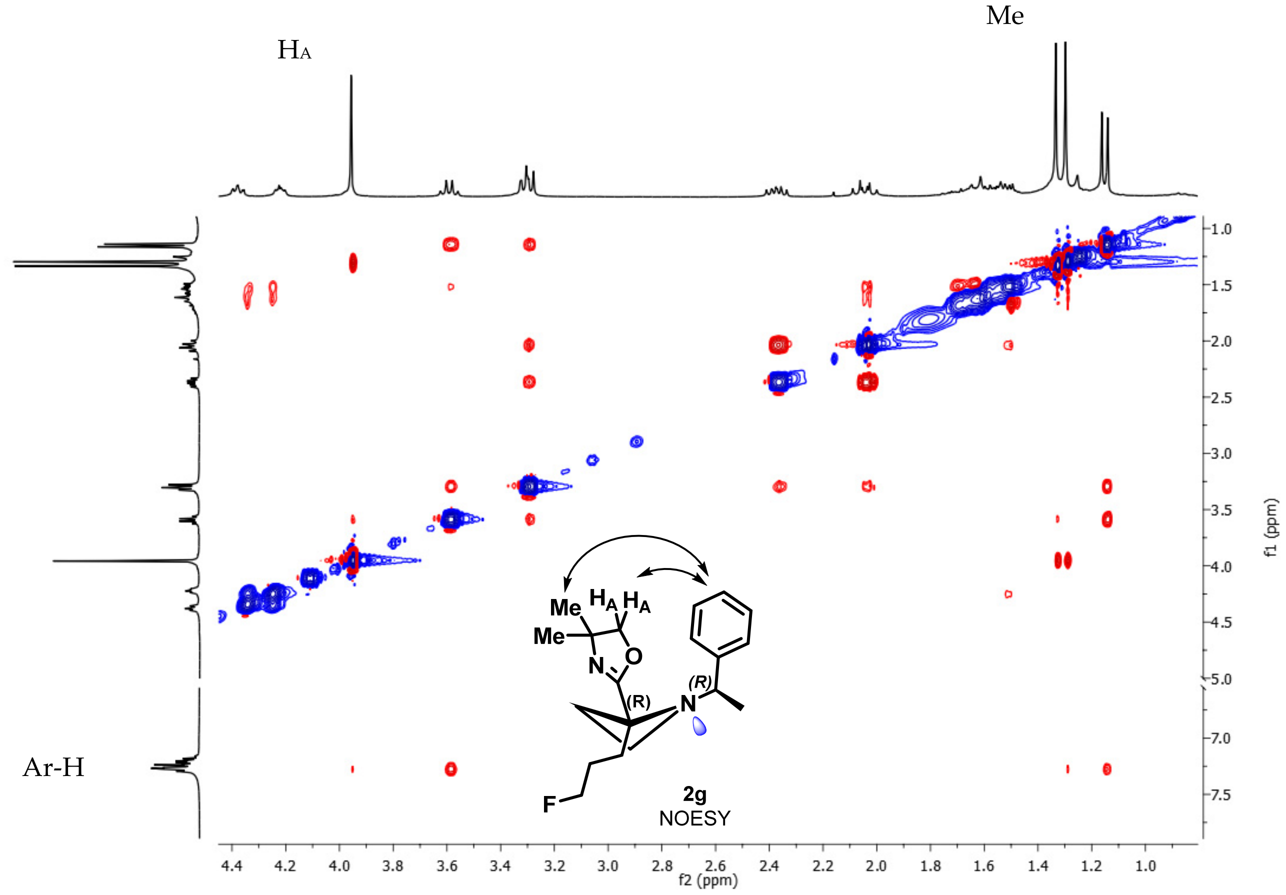
HA
Me
Ar-H
Me
HA HA
Me
N
O
(R)
(R)
N
F
2g
NOESY
f2 (ppm)
f1 (ppm)

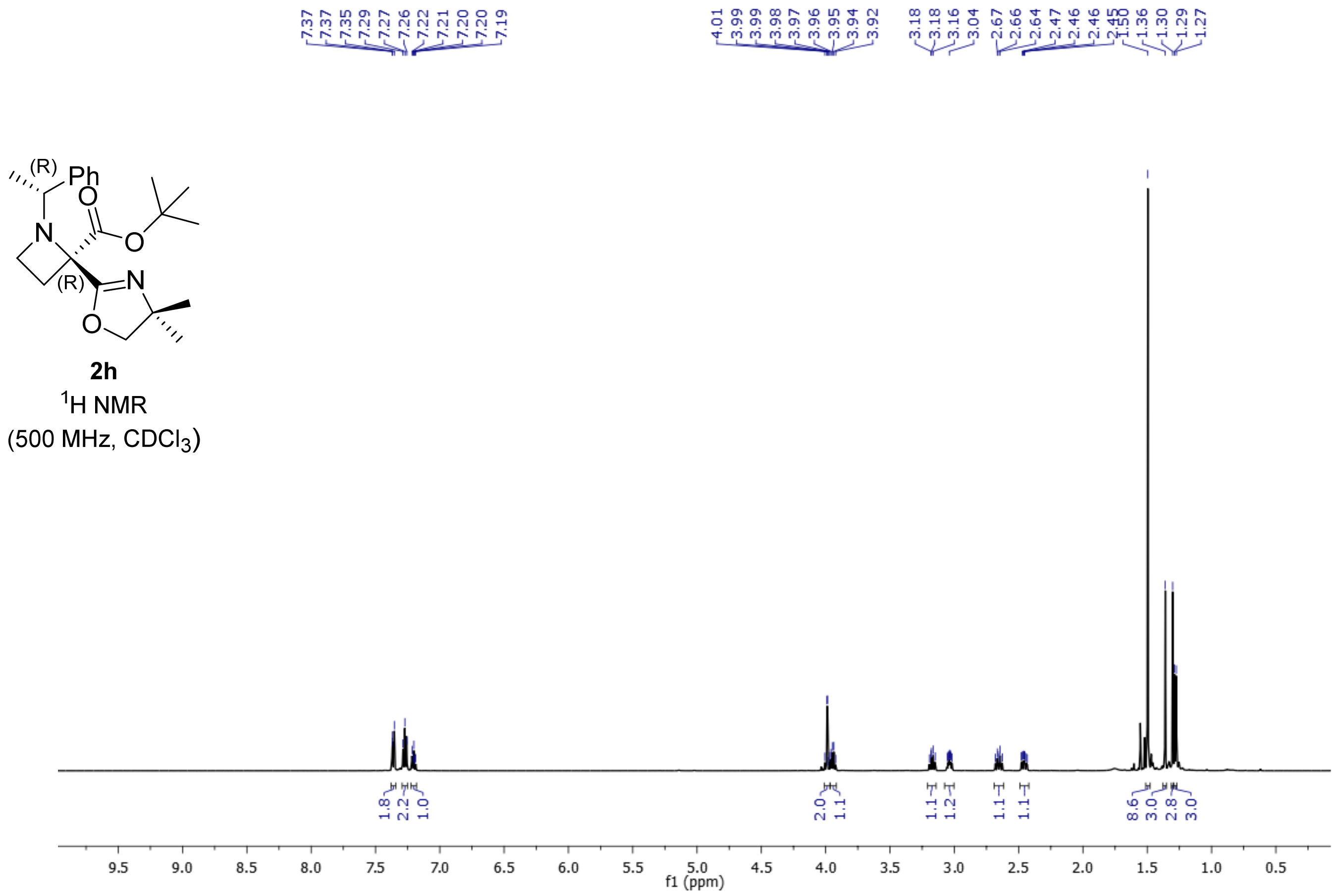
(R)
Ph
O
O
N
(R)
N
O
2h
^{1}H NMR
(500 MHz, $CDCl_3$)
f1 (ppm)

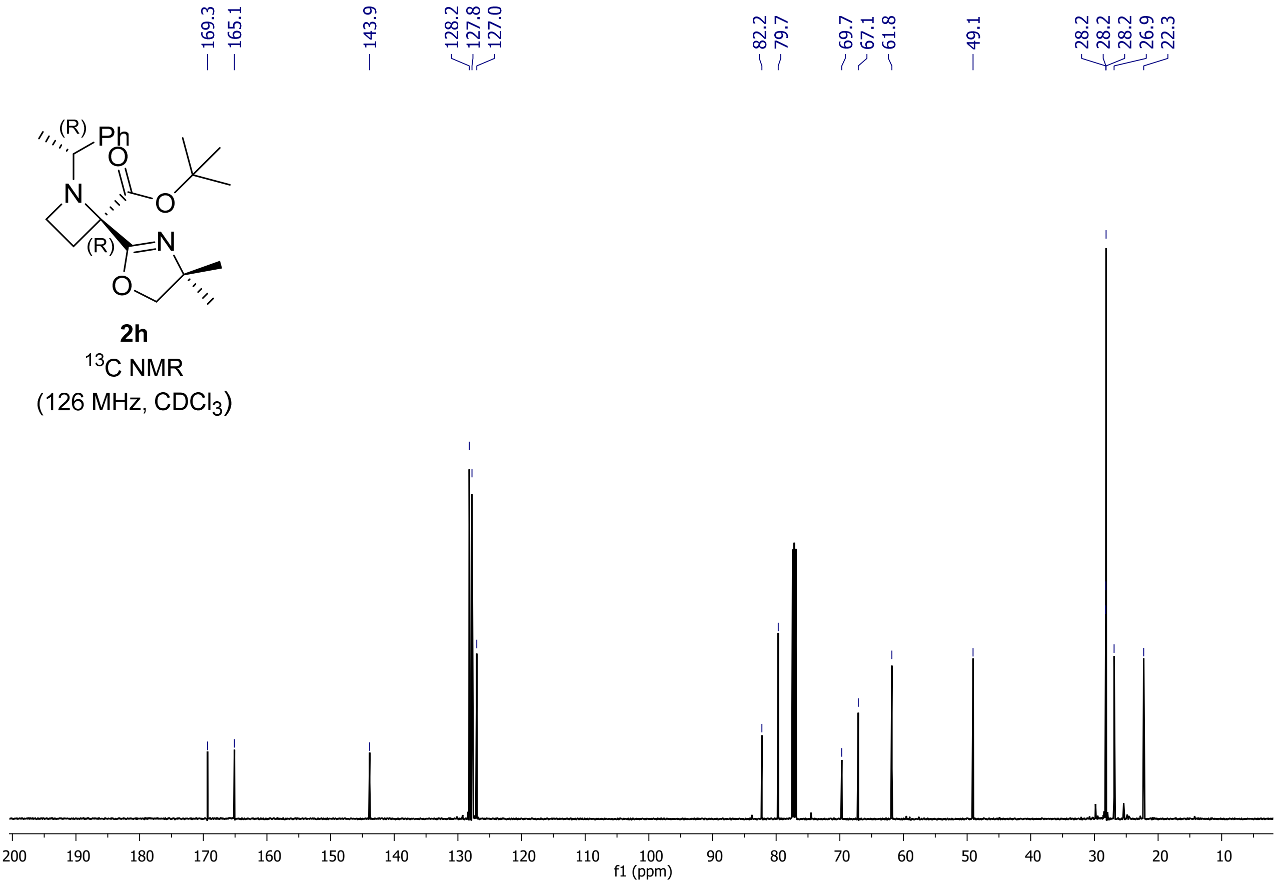
169.3
165.1
143.9
128.2
127.8
127.0
82.2
79.7
69.7
67.1
61.8
49.1
28.2
28.2
28.2
26.9
22.3
(R)
Ph
O
N
O
(R)
N
O
2h
13C NMR
(126 MHz, CDCl3)
f1 (ppm)

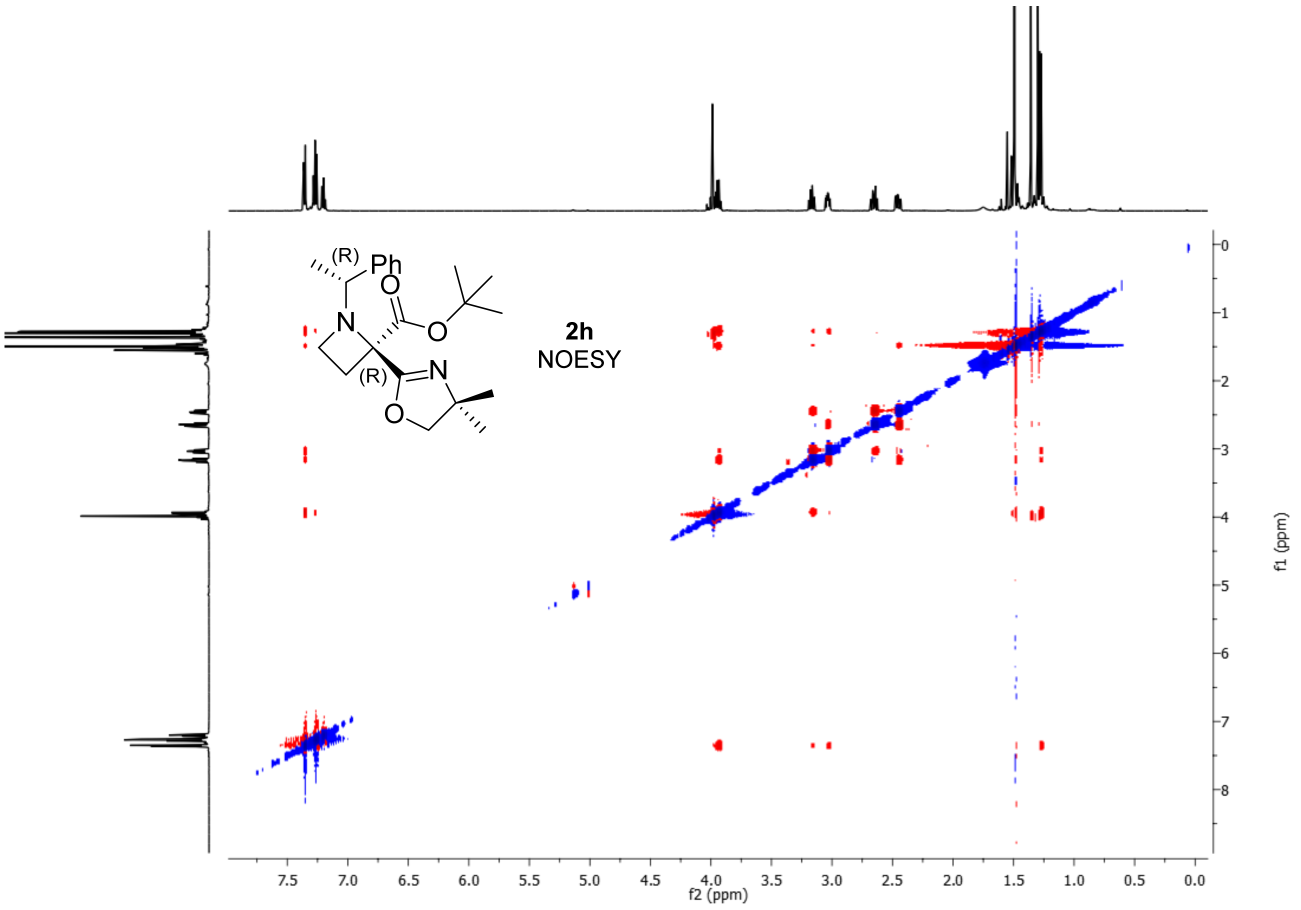
(R)
Ph
O
N
O
(R)
N
O
2h
NOESY
f2 (ppm)
f1 (ppm)

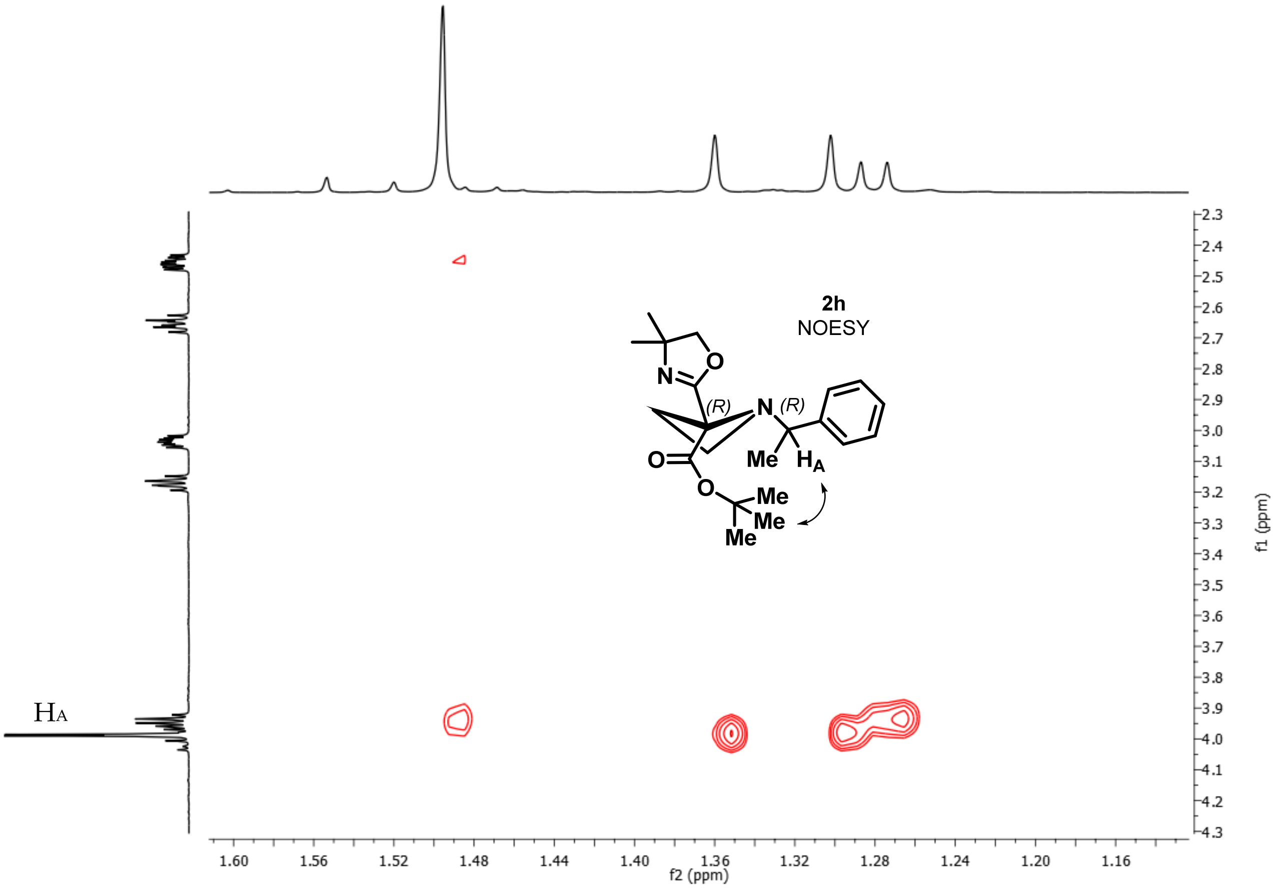
2h
NOESY
HA
f2 (ppm)
f1 (ppm)

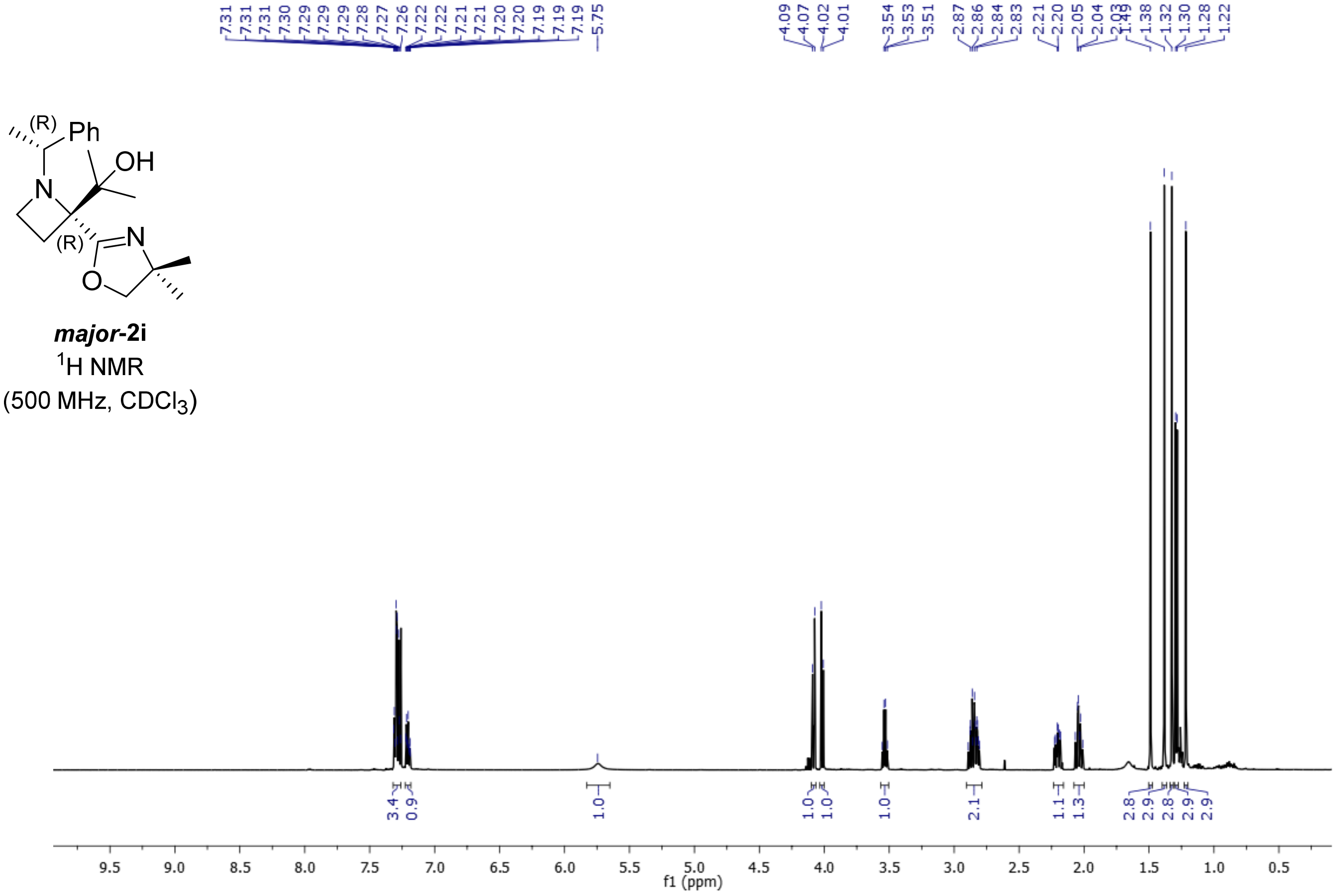
major-2i
1H NMR
(500 MHz, CDCl3)
f1 (ppm)

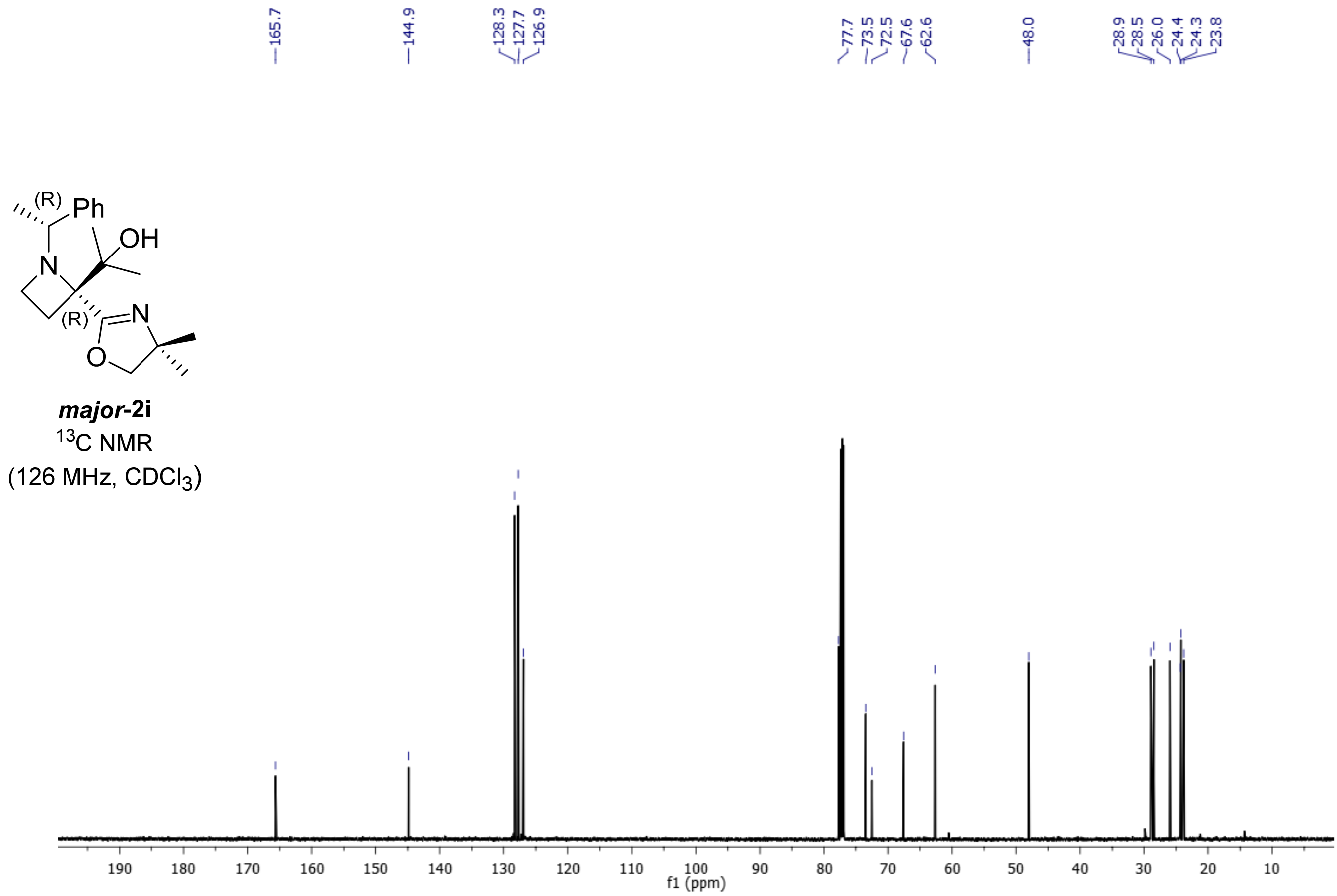
165.7
144.9
128.3
127.7
126.9
77.7
73.5
72.5
67.6
62.6
48.0
28.9
28.5
26.0
24.4
24.3
23.8
(R)
Ph
OH
N
(R)
N
O
***major*-2i**
^{13}C NMR
(126 MHz, $CDCl_3$)
f1 (ppm)

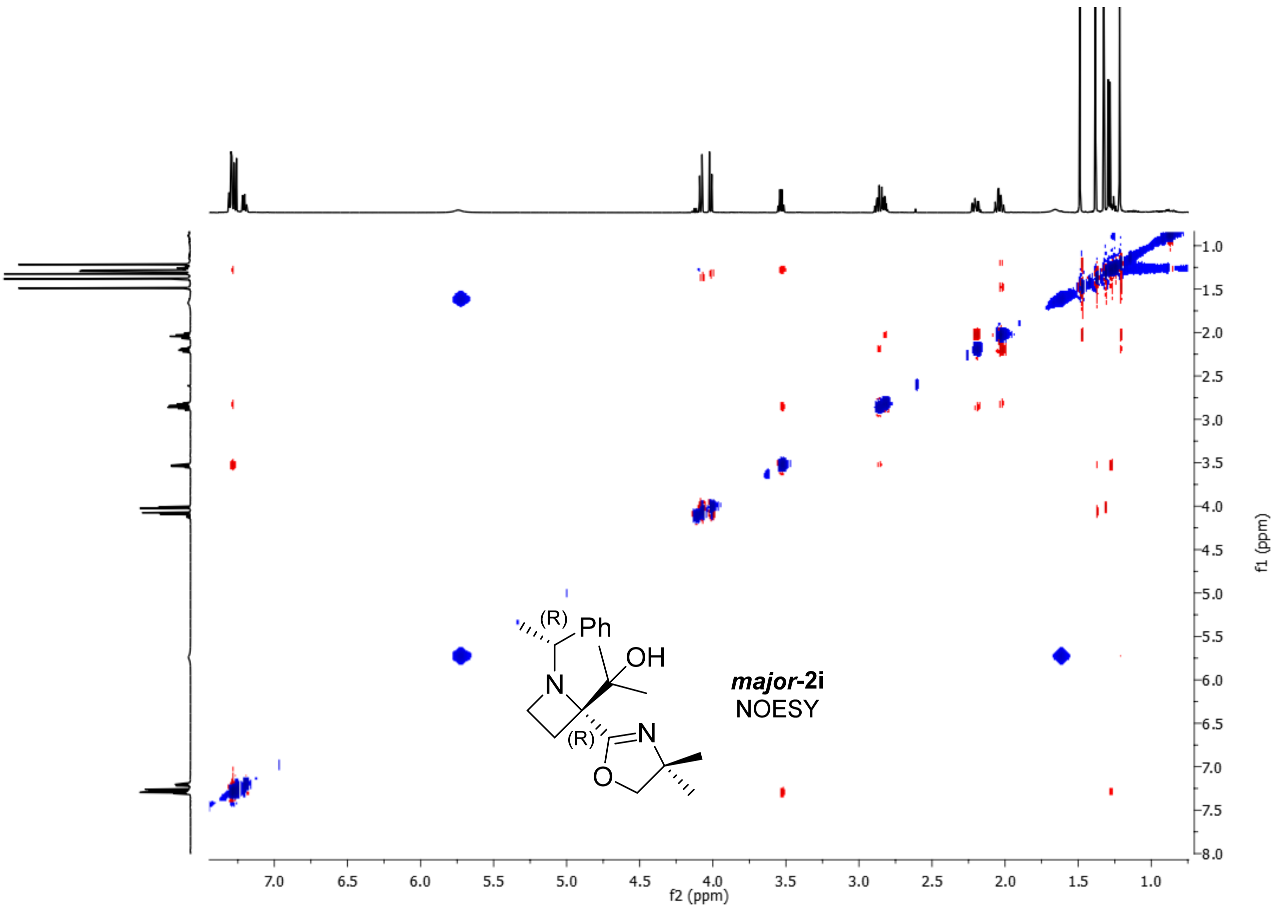
(R)
Ph
OH
N
(R)
N
O
***major*-2i**
NOESY
f1 (ppm)
f2 (ppm)

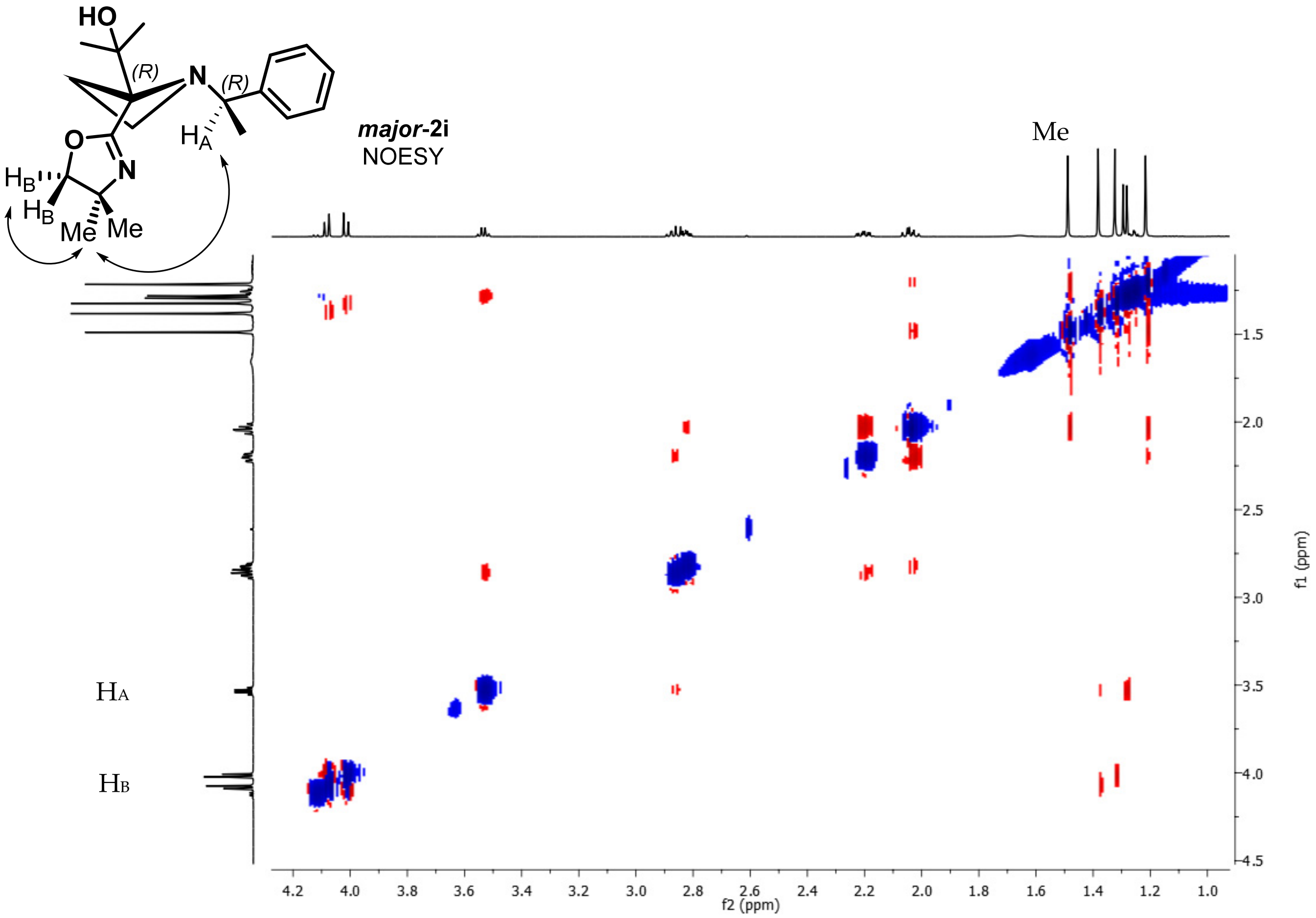
HO
(R)
N
(R)
H_A
O
N
H_B
H_B
Me
Me
***major*-2i**
NOESY
Me
H_A
H_B
f2 (ppm)
f1 (ppm)

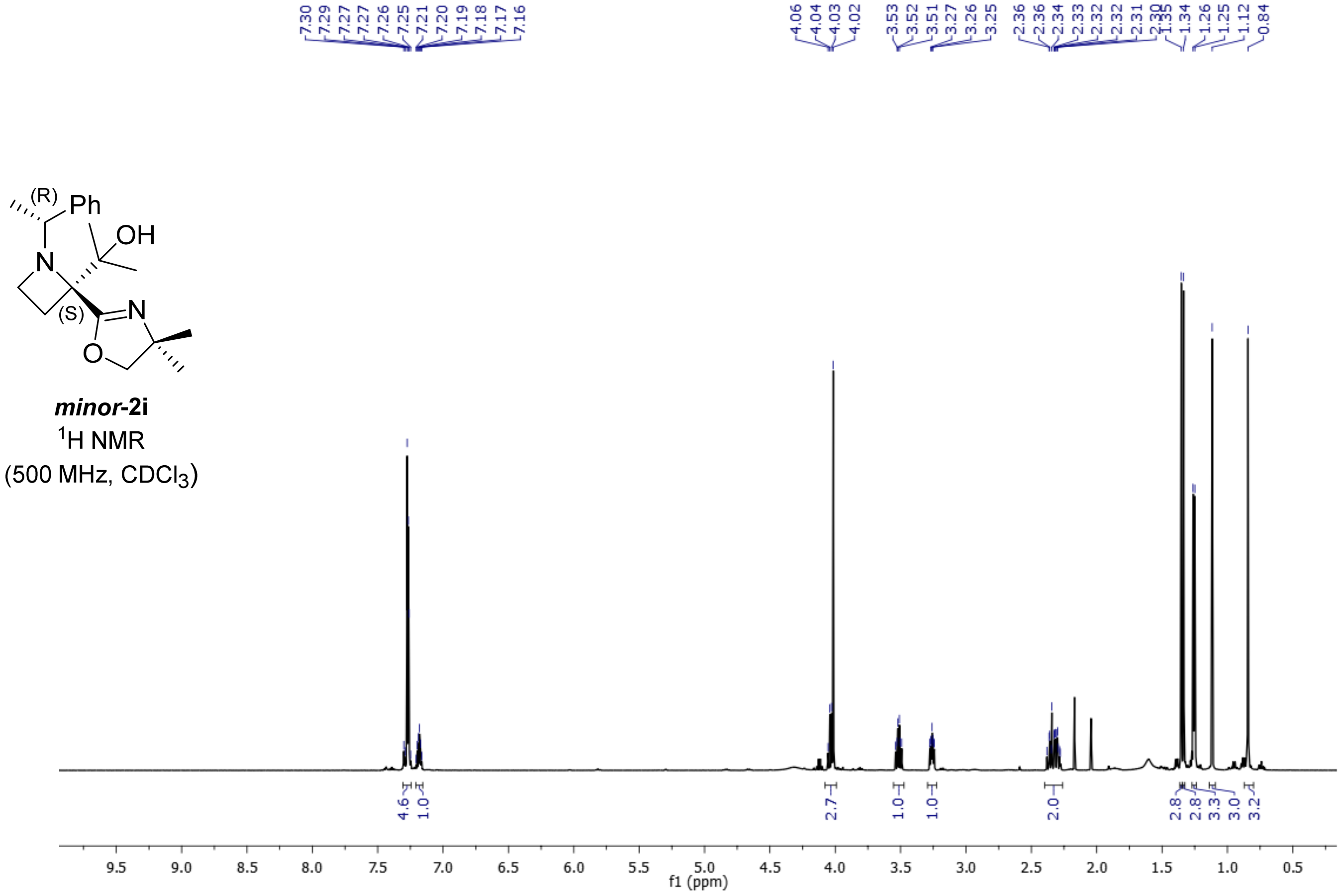
(R)
Ph
OH
N
(S)
N
O
***minor*-2i**
^{1}H NMR
(500 MHz, $CDCl_3$)
f1 (ppm)

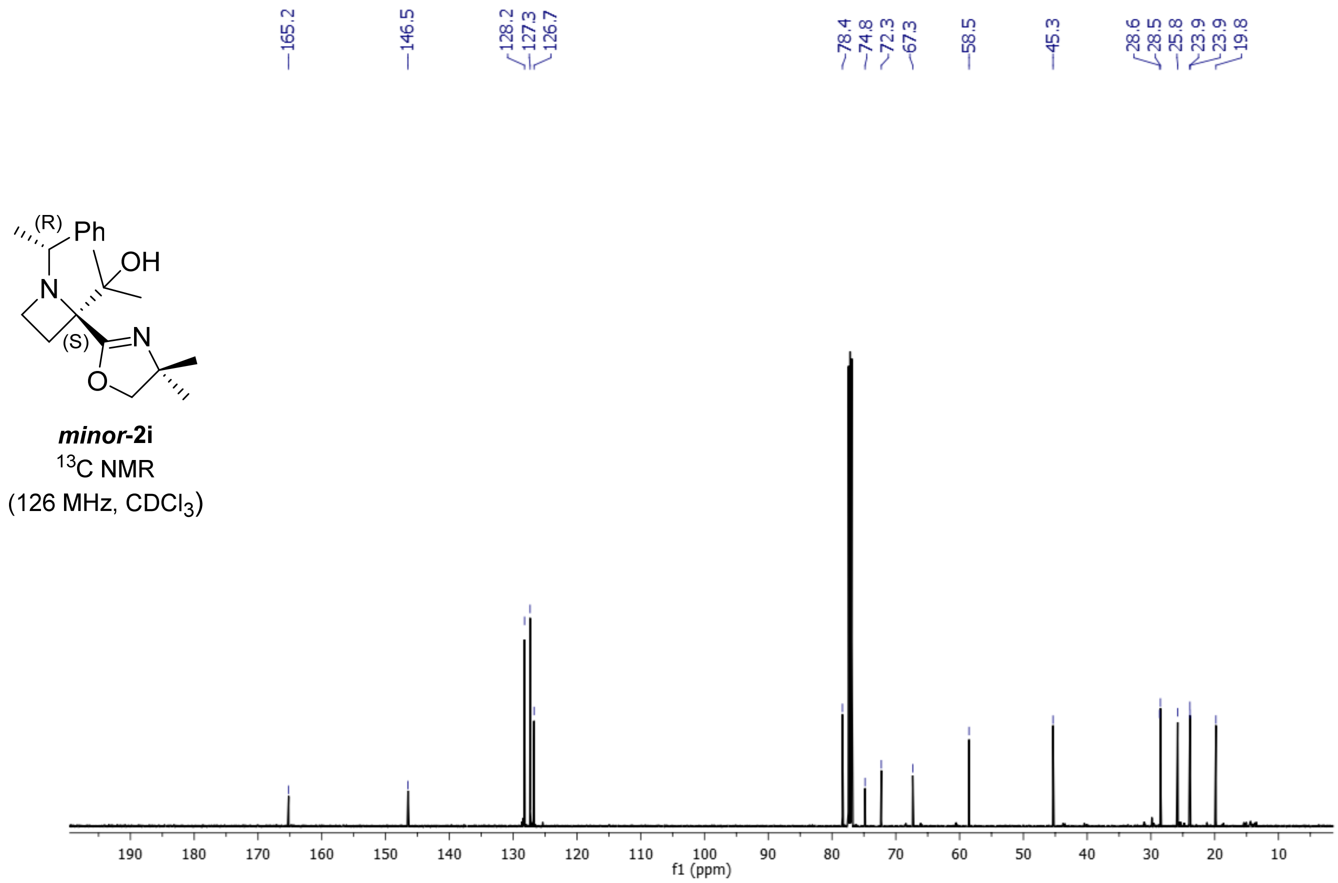

165.2
146.5
128.2
127.3
126.7
78.4
74.8
72.3
67.3
58.5
45.3
28.6
28.5
25.8
23.9
23.9
19.8
(R)
Ph
OH
N
(S)
N
O
***minor*-2i**
^{13}C NMR
(126 MHz, $CDCl_3$)
f1 (ppm)

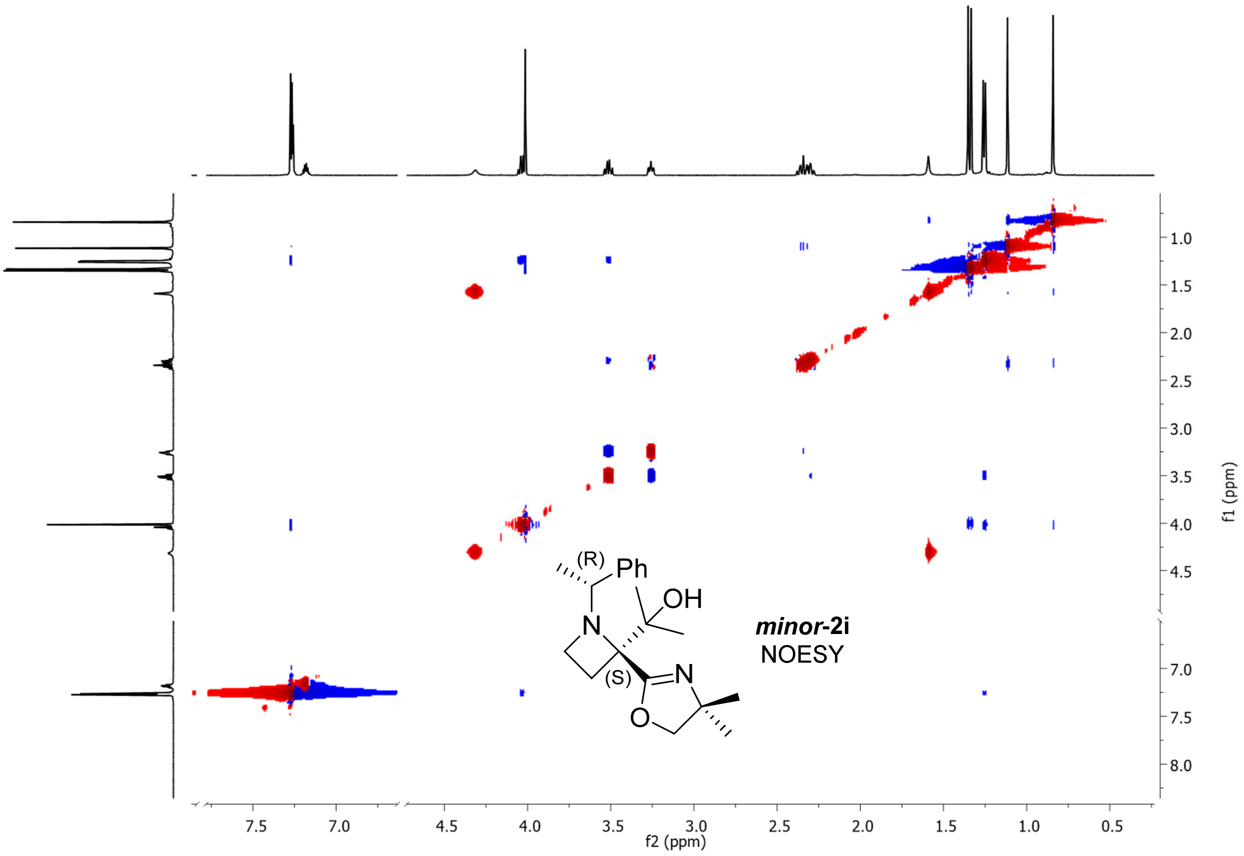

(R)
Ph
OH
N
(S)
N
O
***minor*-2i**
NOESY
f2 (ppm)
f1 (ppm)

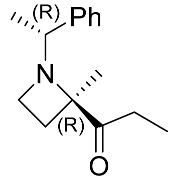

3a

^{1}H NMR

(500 MHz, $CDCl_3$)

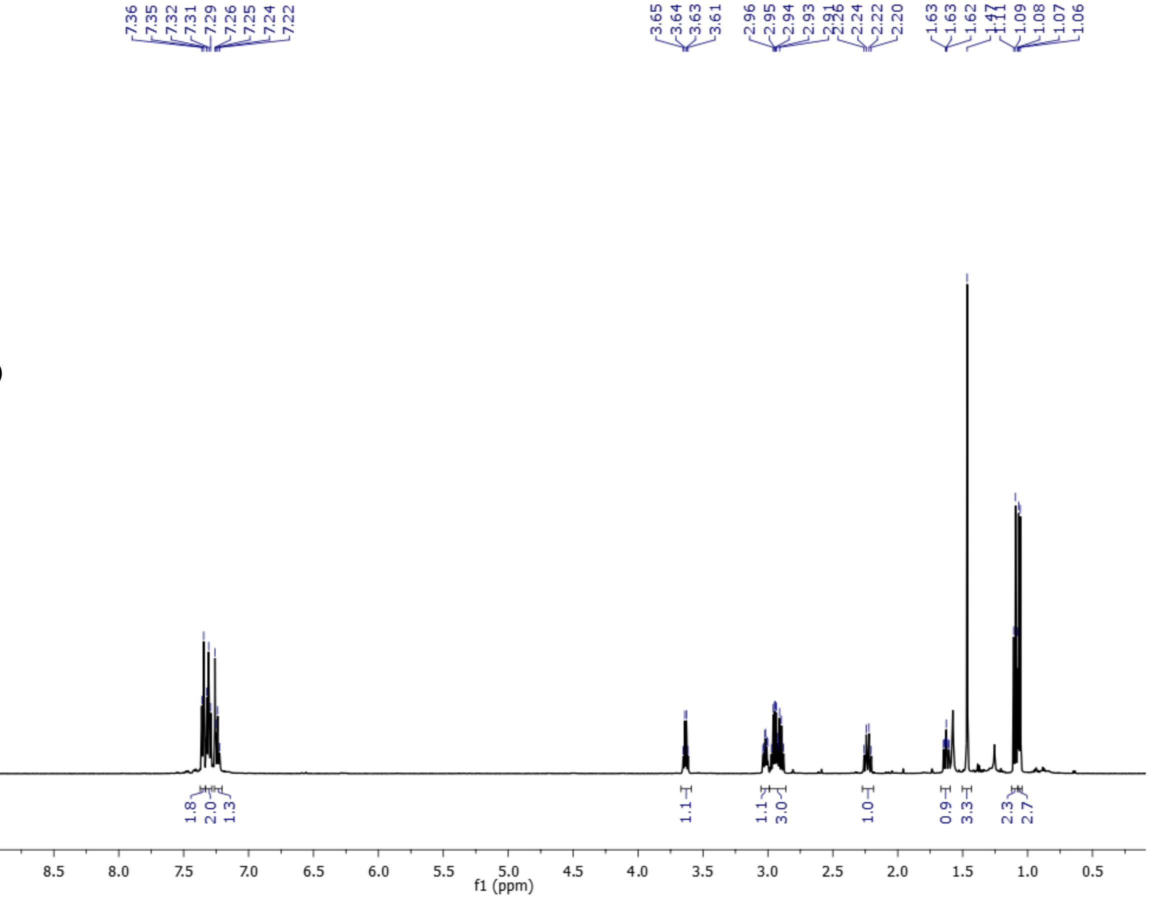

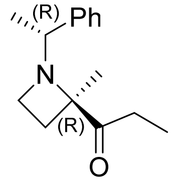

3a

^{13}C NMR

(126 MHz, $CDCl_3$)

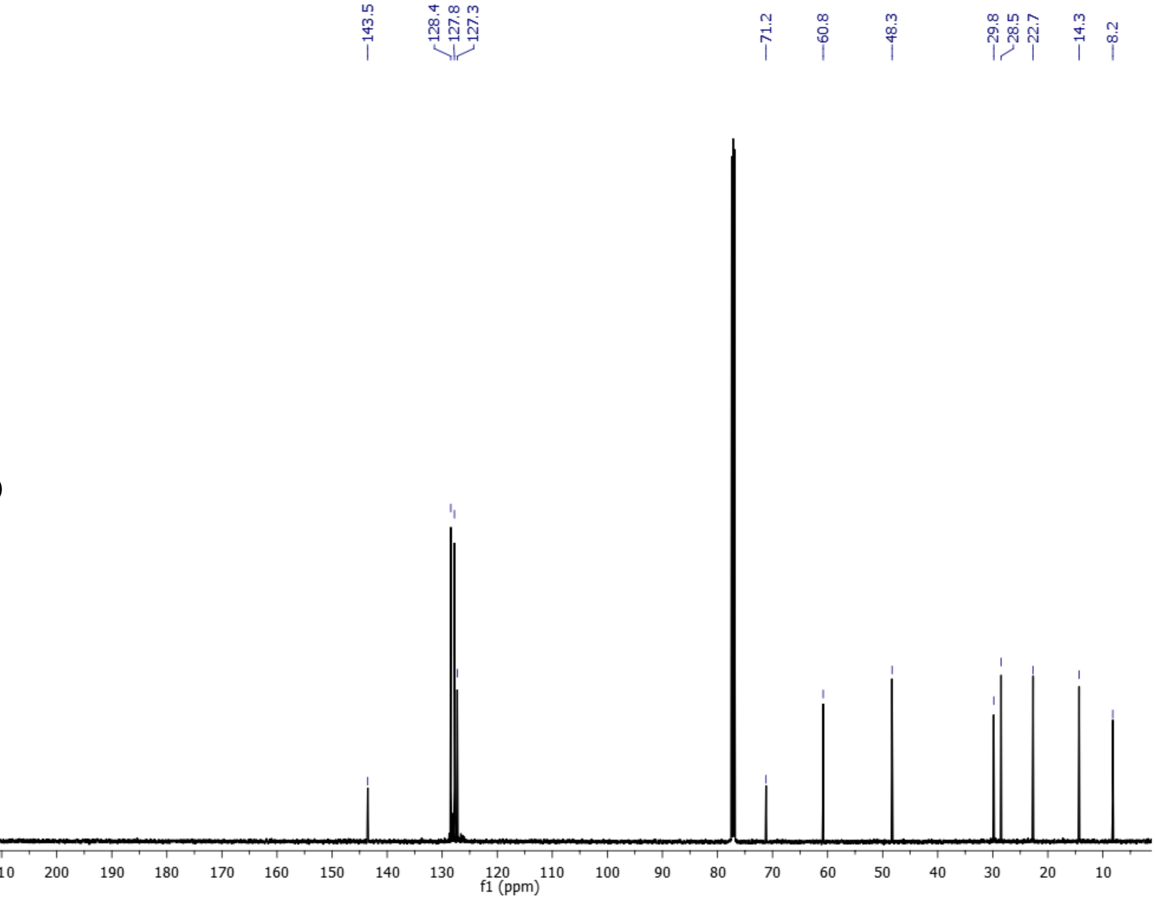

3b

^{1}H NMR

(500 MHz, $CDCl_3$)

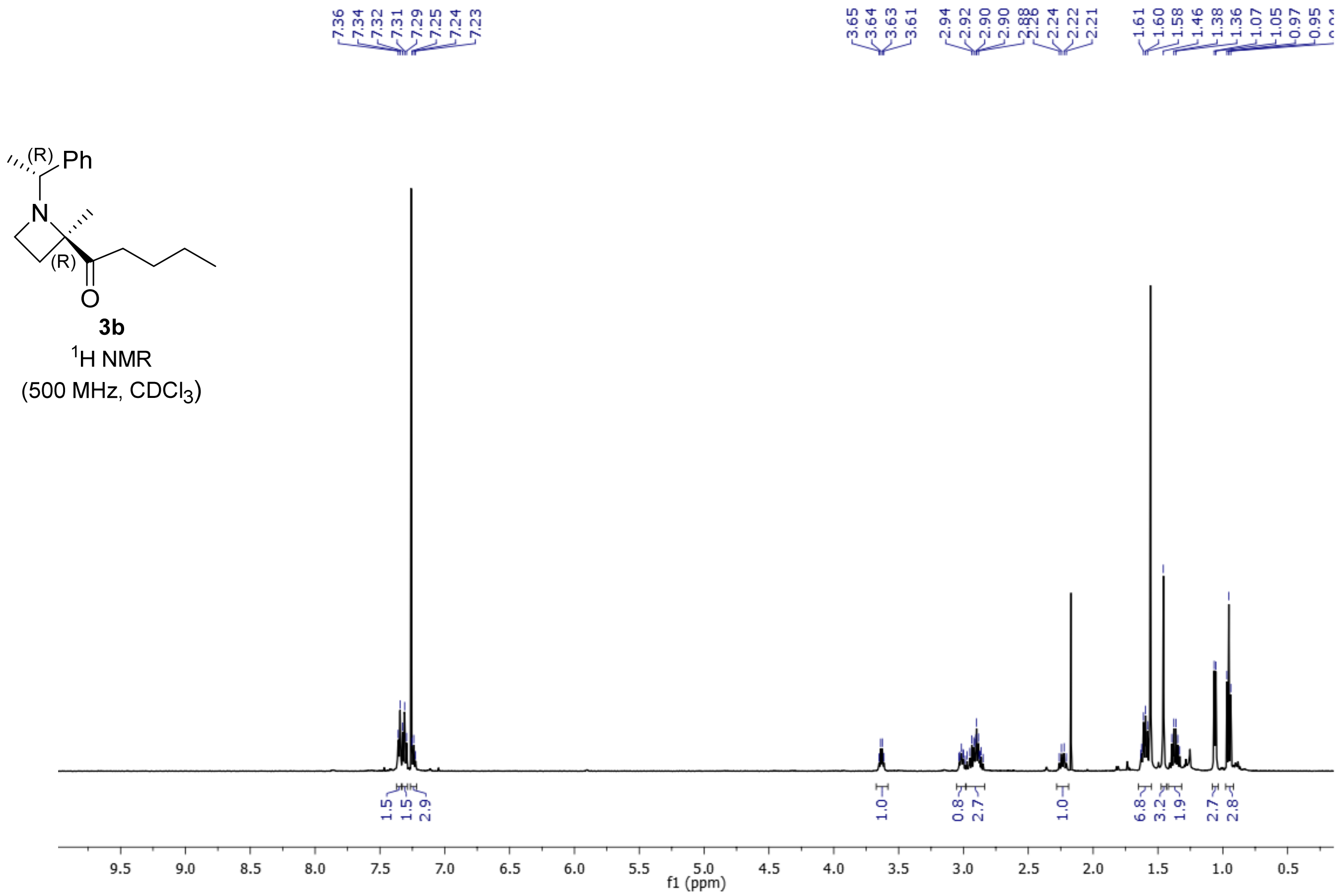

3b

^{13}C NMR

(126 MHz, $CDCl_3$)

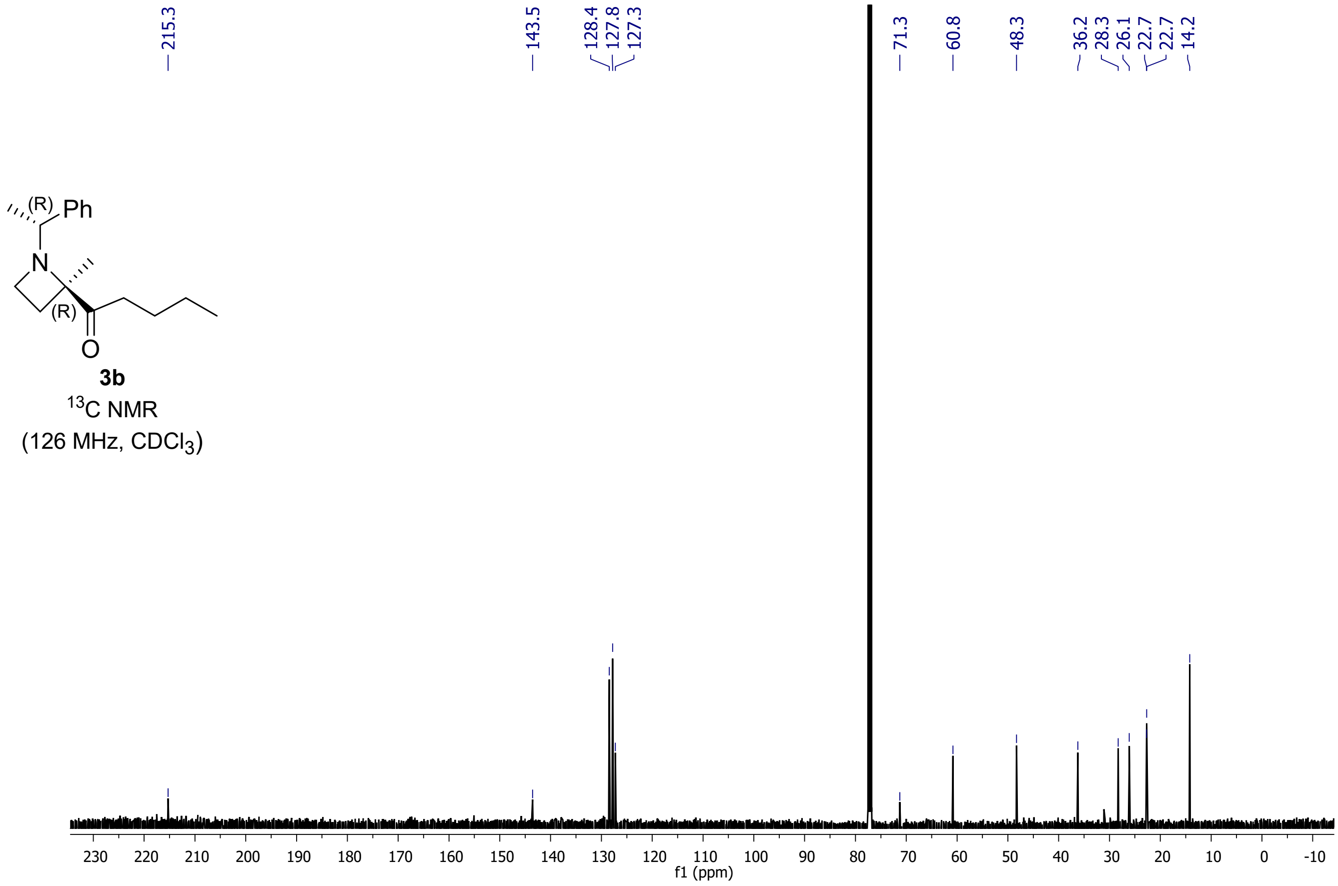

4. X-RAY STRUCTURE AND CIF OF *minor*-2i

2-((S)-2-(4,4-dimethyl-4,5-dihydrooxazol-2-yl)-1-((R)-1-phenylethyl)azetidin-2-yl)propan-2-ol

Compound ***minor*-2i** has been crystallized in order to solve stereochemical attribution, which was particularly tricky because of an unusual dr = 35:65 and weak chemical shift similarity with other products. ***minor*-2i** is the minor diastereoisomer formed and the C2 absolute stereochemistry is demonstrated to be (*S*). This evidence shows that, for the first time, the major diasteroisomer is the inverted one, which means that the stereoconvergence of the lithiated intermediates is electrophile dependent. Furthermore, the analysis revealed a *cis* disposition between the C2-oxazolinyl group and the substituent on the nitrogen. Dynamic nitrogen inversion can, however, be justified because of the steric hindrance of the -$C(CH_3)_2OH$ group. Colorless block crystals of the mentioned compound were obtained by slow evaporation of the solvent (dichloromethane) at room temperature. A single crystal (dimensions 0.400 x 0.300 x 0.100 mm) was selected and mounted on a glass fiber for X-ray diffraction measurement. The X-ray diffraction analysis gives the following cell parameters (Table S1).

Table S1. Main crystallographic data for 2-((S)-2-(4,4-dimethyl-4,5-dihydrooxazol-2-yl)-1-((R)-1-phenylethyl)azetidin-2-yl)propan-2-ol.

Molecular formula	$C_{19}H_{28}N_2O_2$
Molecular weight	316.43
Crystal system	monoclinic
Space group	$P2_1$
Z, molecules/unit cell	2
a, Å	9.065(2)
b, Å	9.604(1)
c, Å	10.849(3)
β, (deg.)	104.09(2)
V, Å^3	916.101(3)
Calculated density, g/cm^3	1.147
R_F factor	0.053
Temperature, K	293

For each atom at the general position x, y, z (x, y, z are the fractional coordinates in the unit cell), there are atoms at symmetry-equivalent positions in -x, y+1/2, -z).

A total of 4197 reflections were collected at 293 K in the θ range from 2.316 to 27.504°, of which 3038 were observed ($I > 2\sigma(I)$). The final values of agreement factors were R = 5.33% and wR = 12.42%, the number of refined parameters was 216, and the maximum and minimum residual densities were $\Delta\varrho max = 0.44$ and $\Delta\varrho min = –0.68$. The procedure of determination of the complete structure has provided the following results (Tables S2–S5).

Table S2. Unit cell content of 2-((S)-2-(4,4-dimethyl-4,5-dihydrooxazol-2-yl)-1-((R)-1-phenylethyl)azetidin-2-yl)propan-2-ol.

Atom	Symbol	Number in Cell	Atomic Number	Weight	Radius
Hydrogen	H	56	1	1.01	0.320
Carbon	C	38	6	12.01	0.770
Oxygen	O	4	8	16.00	0.730
Nitrogen	N	4	7	14.01	0.750

Table S3. Atomic positions of 2-((S)-2-(4,4-dimethyl-4,5-dihydrooxazol-2-yl)-1-((R)-1-phenylethyl)azetidin-2-yl)propan-2-ol.

Atom	*x*	*y*	*z*	U(Å²)
O_1	0.790	0.498	0.498	0.042
O_2	1.00	0.194	0.322	0.053
N_1	0.692	0.332	0.265	0.035
N_2	0.938	0.307	0.079	0.041
C_1	0.846	0.395	0.127	0.030
C_2	0.546	0.106	0.267	0.039
C_3	0.841	0.395	0.261	0.031
C_4	0.981	0.335	0.358	0.040
C_5	0.920	0.342	-0.057	0.041
C_6	0.629	0.210	0.187	0.043
C_7	0.776	0.536	0.295	0.039
C_8	0.818	0.473	-0.074	0.045
C_9	0.390	0.052	0.396	0.057
C_{10}	0.618	0.469	0.257	0.044
C_{11}	0.454	0.147	0.329	0.046
C_{12}	0.437	-0.084	0.404	0.056
C_{13}	0.952	0.322	0.490	0.060
C_{14}	0.608	-0.031	0.275	0.054
C_{15}	1.121	0.426	0.361	0.058
C_{16}	0.845	0.222	-0.139	0.063
C_{17}	0.543	-0.126	0.344	0.062
C_{18}	1.075	0.374	-0.082	0.067
C_{19}	0.511	0.245	0.066	0.073
H_6	0.714	0.164	0.163	0.052
H_{7A}	0.808	0.560	0.384	0.048<br>048
H_{7B}	0.790	0.613	0.241	0.048
H_{8A}	0.869	0.552	-0.101	0.054
H_{8B}	0.723	0.457	-0.137	0.054
H_9	0.315	0.081	0.436	0.069
H_{10A}	0.558	0.483	0.319	0.054
H_{10B}	0.560	0.494	0.172<br>17.1	0.054
H_{11}	0.424	0.240	0.325	0.056
H_{12}	0.395	-0.148	0.451	0.068
H_{13A}	0.861	0.269	0.485	0.090
H_{13B}	0.941	0.413	0.522	0.090
H_{13C}	1.037	0.275	0.545	0.090
H_{14}	0.628	-0.061	0.234	0.065
H_{15A}	1.144	0.423	0.280	0.088
H_{15B}	1.206	0.391	0.425	0.088
H_{15C}	1.100	0.520	0.382	0.088
H_{16A}	0.749	0.202	-0.122	0.096
H_{16B}	0.910	0.141	-0.121	0.096
H_{16C}	0.830	0.247	-0.227	0.096
H_{17}	0.573	-0.219	0.348	0.075

H_{18A}	1.123	0.449	-0.027	0.101
H_{18B}	1.062	0.401	-0.169	0.101
H_{18C}	1.138	0.292	-0.065	0.101
H_{19A}	0.552	0.312	0.017	0.110
H_{19B}	0.422	0.283	0.087	0.110
H_{19C}	0.483	0.162	0.016	0.110
H_{100}	1.022	0.192	0.244	0.110

Table S4. Bond lengths of 2-((S)-2-(4,4-dimethyl-4,5-dihydrooxazol-2-yl)-1-((R)-1-phenylethyl)azetidin-2-yl)propan-2-ol.

Bond Lengths (Å)			
O_1 - C_{17}	1.360(4)	C_3 – C_7	1.553(6)
O_1 - C_8	1.451(4)	C_4 - C_{13}	1.518(6)
O_2 - C_4	1.439(5)	C_4 - C_{15}	1.543(6)
N_1 – C_3	1.489(5)	C_5 – C_8	1.541(6)
N_1 – C_6	1.472(5)	C_5 - C_{16}	1.517(6)
N_1 – C_{10}	1.477(5)	C_5 - C_{18}	1.526(6)
N_2 – C_1	1.267(5)	C_6 - C_{19}	1.525(5)
N_2 – C_5	1.490(5)	C_7 – C_{10}	1.531(5)
C_1 – C_3	1.525(5)	C_9 – C_{11}	1.384(7)
C_2 – C_6	1.532(5)	C_9 – C_{12}	1.378(7)
C_2 – C_{11}	1.382(6)	C_{12} – C_{17}	1.356(7)
C_2 – C_{14}	1.381(6)	C_{14} – C_{17}	1.396(7)
C_3 – C_4	1.552(4)		

Table S5. Bond angles of 2-((S)-2-(4,4-dimethyl-4,5-dihydrooxazol-2-yl)-1-((R)-1-phenylethyl)azetidin-2-yl)propan-2-ol.

Bond Angles (°)			
C_1 – O_1 – C_8	106.4(3)	C_3 – C_4 – C_{13}	111.6(3)
C_3 – N_1 – C_6	122.4(3)	C_3 – C_4 – C_{15}	109.7(3)
C_3 – N_1 – C_{10}	92.0(3)	C_{13} – C_4 – C_{15}	110.8(3)
C_6 – N_1 – C_{10}	124.7(3)	N_2 – C_5 – C_8	103.1(3)
C_1 – N_2 – C_5	108.1(3)	N_2 – C_5 – C_{16}	109.6(3)
O_1 – C_1 – N_2	117.7(3)	N_2 – C_5 – C_{18}	109.9(3)
O_1 – C_1 – C_3	115.2(3)	C_8 – C_5 – C_{16}	112.3(3)
N_2 – C_1 – C_3	127.0(3)	C_8 – C_5 – C_{18}	111.6(3)
C_6 – C_2 – C_{11}	120.8(3)	C_{16} – C_5 – C_{18}	110.2(3)
C_6 – C_2 – C_{14}	120.9(3)	N_1 – C_6 – C_2	110.1(3)
C_{11} – C_2 – C_{14}	118.2(4)	N_1 – C_6 – C_{19}	114.5(3)
N_1 – C_3 – C_1	11.4(3)	C_2 – C_6 – C_{19}	109.9(3)
N_1 – C_3 – C_4	115.1(3)	C_3 – C_7 – C_{10}	87.6(3)
N_1 – C_3 – C_7	87.2(3)	O_1 – C_8 – C_5	104.8(3)
C_1 – C_3 – C_4	115.5(3)	C_{11} – C_9 – C_{12}	120.1(4)
C_1 – C_3 – C_7	112.1(3)	N_1 – C_{10} – C_7	88.5(3)
C_4 – C_3 – C_7	117.4(3)	C_2 – C_{11} – C_9	120.8(4)
O_2 – C_4 – C_3	109.6(3)	C_9 – C_{12} – C_{17}	119.9(4)

$O_2 – C_4 – C_{13}$	104.9(3)	$C_2 – C_{14} – C_{17}$	120.7(4)
$O_2 – C_4 – C_{15}$	110.1(3)	$C1_2 – C_{17} – C_{14}$	120.2(4)

The refined structure model with the numbering of the corresponding atoms and the molecular packing is shown in Figures S2 and S3.

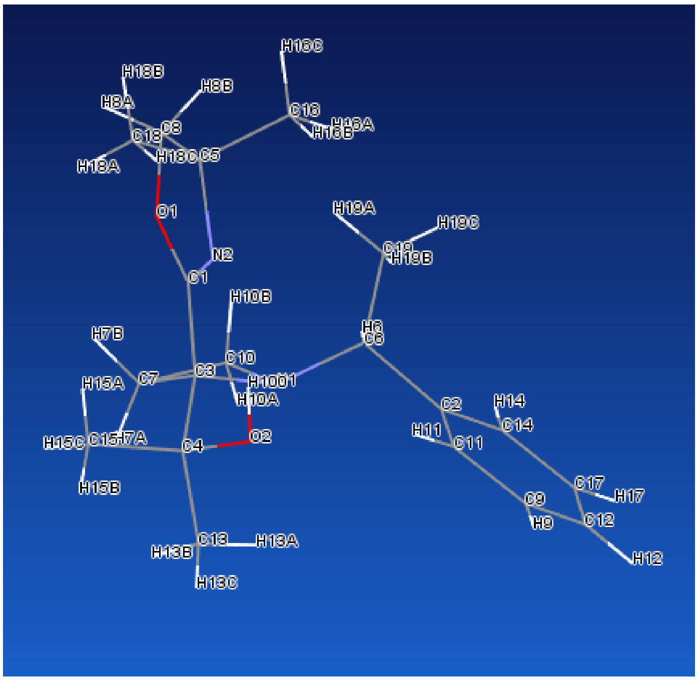

Figure S2. Final structural model of 2-((S)-2-(4,4-dimethyl-4,5-dihydrooxazol-2-yl)-1-((R)-1-phenylethyl)azetidin-2-yl)propan-2-ol with the numbering of atoms.

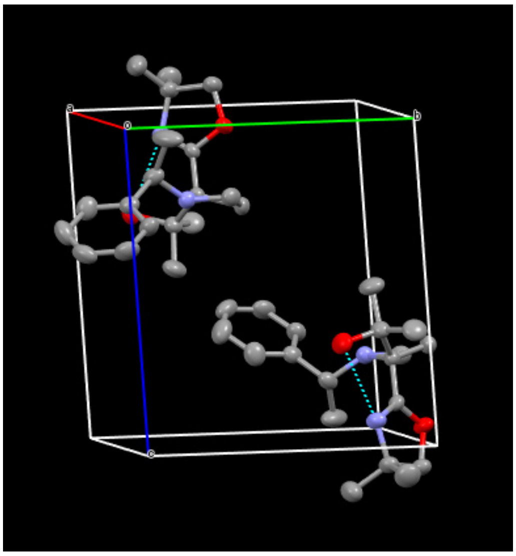

Figure S3. Crystal packing of 2-((S)-2-(4,4-dimethyl-4,5-dihydrooxazol-2-yl)-1-((R)-1-phenylethyl)azetidin-2-yl)propan-2-ol with the numbering of atoms. Intramolecular hydrogen bonds are indicated as dashed lines.

Crystal structure solution revealed a cis relationship between the substituent on the nitrogen and the oxazoline group and a puckering angle of the nitrogenated cycle of 23.45°.

Ortep view (50% probability) for **minor-2i**

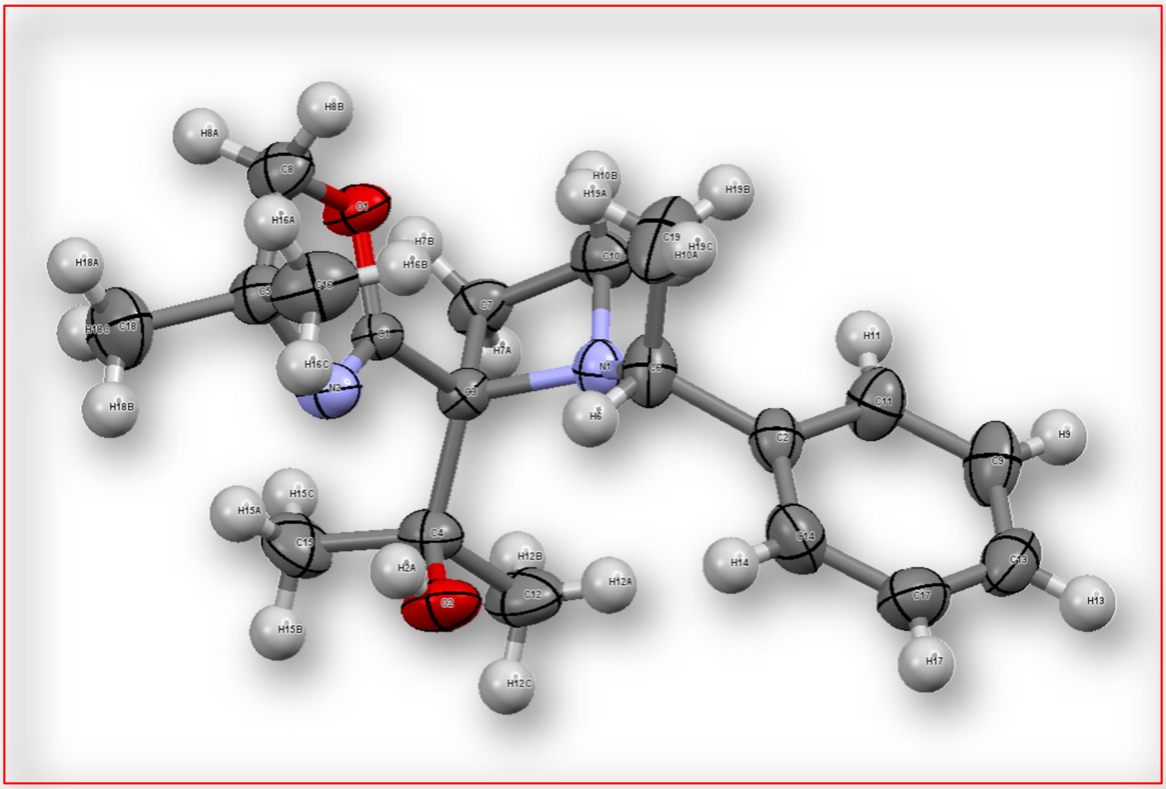

5. DFT CALCULATIONS

DFT Calculations on diastereoisomeric oxazolinylazetidines **2b,c**

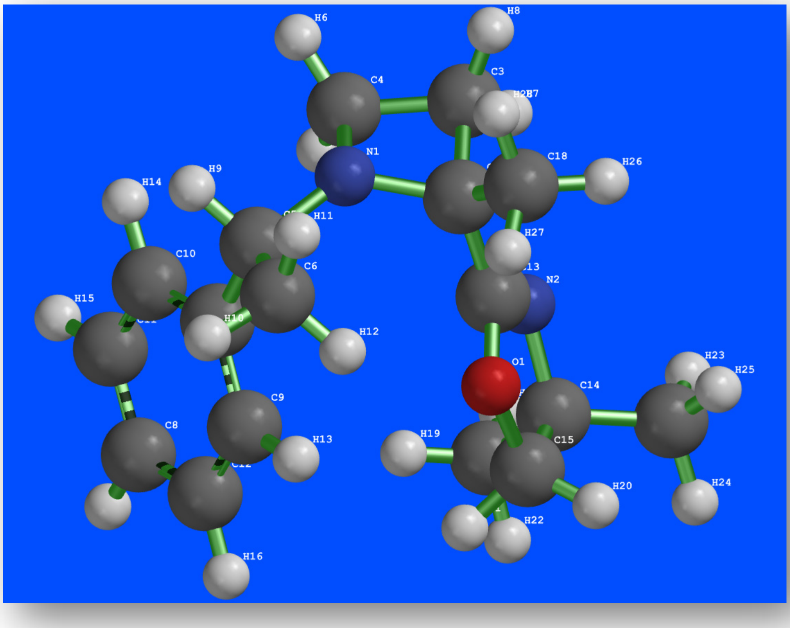

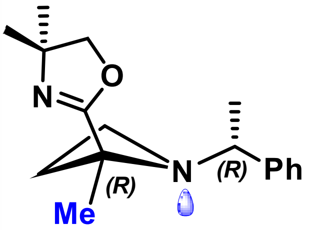

syn-(2R,1'R)-**2b**

** GEOMETRY OPTIMIZATION IN DELOCALIZED INTERNAL COORDINATES **

Job type: Geometry optimization.

Method: RB3LYP
Basis set: 6-31G(D)
Number of shells: 128
Number of basis functions: 348
Multiplicity: 1
SCF model:
A restricted hybrid HF-DFT SCF calculation will be
performed using Pulay DIIS + Geometric Direct Minimization
Searching for a Minimum
Optimization Cycle: 25
Coordinates (Angstroms)
ATOM X Y Z
1 C 0.088704 2.076201 0.240237
2 C 0.338866 2.409914 1.741096
3 N 1.493287 1.562253 0.204250
4 C 1.576093 1.491196 1.666623
5 H 2.521762 1.867100 2.082126
6 H 1.374587 0.494756 2.089069
7 H -0.453359 2.110726 2.425524
8 H 0.594357 3.463620 1.878771
9 C 2.160886 0.603996 -0.683475
10 H 3.225896 0.734470 -0.435531
11 C 1.992302 1.019097 -2.149177
12 H 2.600987 0.380384 -2.797786
13 H 2.315529 2.056658 -2.275073
14 H 0.951905 0.943562 -2.476737
15 C 1.873819 -0.884098 -0.426807
16 C 1.449506 -3.624449 0.117213
17 C 0.963415 -1.636795 -1.182146
18 C 2.575070 -1.540284 0.596335
19 C 2.363583 -2.891144 0.874655
20 C 0.755779 -2.992260 -0.916186
21 H 0.415903 -1.167681 -1.992899
22 H 3.310543 -0.984633 1.174938
23 H 2.921658 -3.372346 1.673936
24 H 0.056027 -3.558586 -1.526534
25 H 1.288297 -4.679670 0.321456
26 C -1.027102 1.054108 0.092691
27 N -1.728929 0.557999 1.032035
28 C -2.736096 -0.338599 0.422674
29 C -2.416331 -0.256474 -1.102935
30 O -1.317988 0.680136 -1.195731

31 H -3.245125 0.135516 -1.701577
32 H -2.089204 -1.211824 -1.524848
33 C -2.562390 -1.758921 0.977061
34 H -2.695615 -1.758966 2.063722
35 H -1.562658 -2.143777 0.753537
36 H -3.303592 -2.439123 0.538762
37 C -4.137675 0.201493 0.743173
38 H -4.295801 0.228782 1.825991
39 H -4.911625 -0.434265 0.295471
40 H -4.260528 1.219225 0.356224
41 C -0.173607 3.269426 -0.685649
42 H -1.120984 3.753556 -0.417046
43 H -0.236115 2.969233 -1.734620
44 H 0.637194 3.995035 -0.573324
Point Group: c1 Number of degrees of freedom: 126
Energy is -846.952250732
Hessian Updated using BFGS Update
internal optimization with constraints (0)
126 Hessian modes will be used to form the next step
Hessian Eigenvalues:
0.002951 0.003013 0.003337 0.003853 0.004226 0.004854
0.007664 0.012062 0.015806 0.018422 0.021829 0.022589
0.023104 0.024556 0.024687 0.026494 0.026713 0.026985
0.028708 0.030084 0.031595 0.034199 0.036008 0.037359
0.040084 0.040749 0.043193 0.043412 0.043699 0.043976
0.044394 0.044490 0.044871 0.045707 0.046004 0.046653
0.048837 0.050449 0.053283 0.055663 0.061570 0.062445
0.067576 0.071050 0.079632 0.083124 0.088788 0.092069
0.096184 0.099918 0.106596 0.126770 0.127371 0.128573
0.128910 0.130542 0.131302 0.131955 0.138131 0.143322
0.149388 0.150379 0.153140 0.155138 0.157208 0.158467
0.159398 0.162889 0.165423 0.170180 0.174354 0.177406
0.182590 0.196655 0.200462 0.201642 0.216432 0.224474
0.245980 0.249767 0.254736 0.265527 0.267194 0.273117
0.275928 0.279102 0.292445 0.293204 0.296810 0.301024
0.301665 0.302000 0.302184 0.302498 0.302637 0.302837
0.303136 0.303591 0.304074 0.304669 0.305150 0.305787
0.306655 0.308694 0.309731 0.311818 0.314030 0.314918
0.315594 0.320200 0.329771 0.332298 0.335692 0.337103
0.338277 0.340576 0.345388 0.349419 0.350199 0.356225
0.368708 0.375164 0.391051 0.528243 0.550520 0.586021
Minimum Search - Taking Simple RFO Step

Searching for Lamda that Minimizes Along All modes
Value Taken Lamda = 0.00000000
Step Taken. Stepsize is 0.005461
Maximum Tolerance Cnvgd?
Gradient 0.000129 0.000300 YES
Displacement 0.001855 0.001200 NO
Energy change -0.000001 0.000001 YES
****
** OPTIMIZATION CONVERGED **
****

- Entering anlman on Fri Mar 22 11:44:01 2019 -

Analysis of SCF Wavefunction
Ground-State Mulliken Net Atomic Charges
Atom Charge (a.u.)
----
1 C 0.164964
2 C -0.323251
3 N -0.412776
4 C -0.115681
5 H 0.126299
6 H 0.140730
7 H 0.178303
8 H 0.140929
9 C -0.040974
10 H 0.135867
11 C -0.474372
12 H 0.148117
13 H 0.152719
14 H 0.170257
15 C 0.167246
16 C -0.130491
17 C -0.185455
18 C -0.189253
19 C -0.126985
20 C -0.137616
21 H 0.138614
22 H 0.126289
23 H 0.128229
24 H 0.124571
25 H 0.127597

26 C 0.550684
27 N -0.468384
28 C 0.158209
29 C -0.061680
30 O -0.508583
31 H 0.150502
32 H 0.149419
33 C -0.455701
34 H 0.155849
35 H 0.167494
36 H 0.133184
37 C -0.442124
38 H 0.158528
39 H 0.137530
40 H 0.145596
41 C -0.463823
42 H 0.145106
43 H 0.163904
44 H 0.150415

Sum of atomic charges = 0.000000

Cartesian Multipole Moments

Charge (ESU x 10^10)
0.0000
Dipole Moment (Debye)
X -0.7698 Y -0.7750 Z -0.6749
Tot 1.2840
Quadrupole Moments (Debye-Ang)
XX -115.0772 XY 2.2222 YY -116.1571
XZ 5.4488 YZ -0.1577 ZZ -120.9972
Traceless Quadrupole Moments (Debye-Ang)
QXX 7.0000 QYY 3.7602 QZZ -10.7602
QXY 6.6665 QXZ 16.3465 QYZ -0.4731
Octapole Moments (Debye-Ang^2)
XXX -23.6138 XXY -0.4215 XYY -6.8755
YYY -5.9860 XXZ 1.3959 XYZ -7.5235
YYZ 4.2290 XZZ 0.9476 YZZ -0.5771
ZZZ -6.9852
Traceless Octapole Moments (Debye-Ang^2)
XXX -88.3320 YYY -26.9289 ZZZ -92.5353

XXY 14.6315 XXZ 25.0198 XYY -14.5069
XYZ -112.8522 XZZ 102.8389 YYZ 67.5156
YZZ 12.2974
Hexadecapole Moments (Debye-Ang^3)
XXXX -3062.0617 XXXY 171.3050 XXYY -998.5857
XYYY 132.6624 YYYY -2669.3088 XXXZ 152.3236
XXYZ -32.4479 XYYZ 69.9853 YYYZ -29.5553
XXZZ -677.0529 XYZZ 57.3489 YYZZ -634.0568
XZZZ 113.9105 YZZZ -20.5238 ZZZZ -1047.0599
Traceless Hexadecapole Moments (Debye-Ang^3)
XXXX 2296.1569 XXXY 1727.7922 XXXZ 864.1034
XXYY -3450.1874 XXYZ -2176.9792 XXZZ 1154.0304
XYYY -2329.6829 XYYZ 2305.1612 XYZZ 601.8907
XZZZ -3169.2646 YYYY 4317.8030 YYYZ 586.8346
YYZZ -867.6156 YZZZ 1590.1447 ZZZZ -286.4148

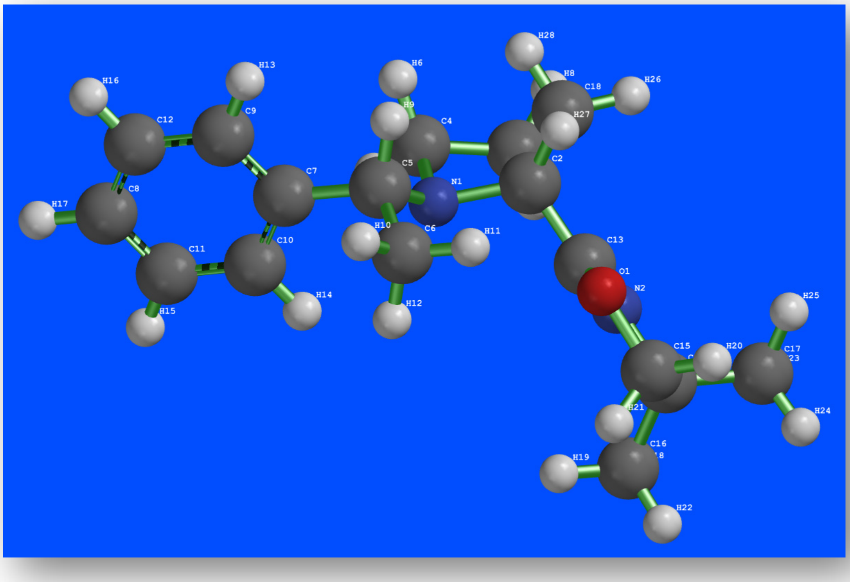

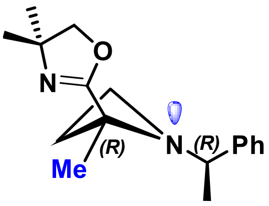

anti-(2R,1'R)-**2b**

** GEOMETRY OPTIMIZATION IN DELOCALIZED INTERNAL COORDINATES **
Method: RB3LYP
Basis set: 6-31G(D)
Number of shells: 128
Number of basis functions: 348
Multiplicity: 1
SCF model:
A restricted hybrid HF-DFT SCF calculation will be
performed using Pulay DIIS + Geometric Direct Minimization

Job type: Geometry optimization.
Method: RB3LYP
Basis set: 6-31G(D)
Number of shells: 128
Number of basis functions: 348
Coordinates (Angstroms)
ATOM X Y Z
1 C -0.772735 1.381804 -0.229840
2 C -0.620410 2.188435 1.085681
3 N 0.503301 0.660881 0.129967
4 C 0.846639 1.715389 1.109269
5 H 1.543905 2.470502 0.710379
6 H 1.248727 1.340970 2.056344
7 H -1.228601 1.774055 1.889683
8 H -0.794026 3.263962 1.000338
9 C 1.509740 0.276281 -0.861685
10 H 1.785531 1.136936 -1.500347
11 C 0.991807 -0.847559 -1.775252
12 H 1.769374 -1.130529 -2.492807
13 H 0.099054 -0.542775 -2.326852
14 H 0.733916 -1.729681 -1.179880
15 C 2.776918 -0.174220 -0.142104
16 C 5.125111 -1.071989 1.122153
17 C 4.034945 0.247228 -0.586371
18 C 2.710421 -1.051682 0.949528
19 C 3.873919 -1.494838 1.577738
20 C 5.202417 -0.199703 0.036168
21 H 4.101902 0.933181 -1.428447
22 H 1.735837 -1.369880 1.308978
23 H 3.804308 -2.170583 2.426676
24 H 6.170428 0.140095 -0.323598
25 H 6.031563 -1.417191 1.612630
26 C -1.974817 0.464127 -0.268338
27 N -2.798721 0.266755 0.680920
28 C -3.813153 -0.698856 0.202381
29 C -3.335543 -1.038651 -1.244838
30 O -2.179945 -0.195664 -1.451491
31 H -4.073770 -0.798936 -2.016732
32 H -3.022487 -2.081692 -1.362632
33 C -3.808277 -1.933069 1.114329
34 H -4.063861 -1.648512 2.140019
35 H -2.817768 -2.400398 1.128603

36 H -4.537633 -2.676944 0.770080
37 C -5.191166 -0.021567 0.209116
38 H -5.449597 0.303583 1.221869
39 H -5.968129 -0.711468 -0.143026
40 H -5.192474 0.860328 -0.440996
41 C -0.721410 2.271556 -1.481515
42 H -1.637639 2.870116 -1.545936
43 H -0.638654 1.687615 -2.401861
44 H 0.126271 2.962936 -1.427786
Point Group: c1 Number of degrees of freedom: 126
Energy is -846.957687064
Hessian Updated using BFGS Update
internal optimization with constraints (0)
126 Hessian modes will be used to form the next step
Hessian Eigenvalues:
0.000786 0.003305 0.003491 0.003645 0.003988 0.004309
0.005234 0.006538 0.016519 0.019994 0.021481 0.022570
0.022833 0.023721 0.024503 0.025589 0.026161 0.027296
0.028136 0.029104 0.030748 0.034493 0.036825 0.038843
0.039653 0.040838 0.043147 0.043415 0.044249 0.044279
0.044450 0.044568 0.044874 0.045649 0.046381 0.046778
0.047381 0.052896 0.053441 0.054738 0.056916 0.060692
0.061832 0.065803 0.073515 0.077025 0.079730 0.084190
0.094954 0.096281 0.108846 0.127641 0.128624 0.128944
0.130481 0.131851 0.131965 0.132449 0.138565 0.139869
0.148328 0.149483 0.151825 0.153023 0.155815 0.158773
0.160411 0.162221 0.164928 0.169037 0.170375 0.174407
0.186910 0.193805 0.198645 0.204650 0.209991 0.218543
0.219483 0.237138 0.244043 0.251561 0.258886 0.266235
0.269144 0.279450 0.283987 0.293968 0.296023 0.299109
0.300609 0.301324 0.301724 0.302336 0.302405 0.302818
0.303087 0.303719 0.303857 0.303996 0.305051 0.305185
0.306305 0.307289 0.308300 0.309271 0.311387 0.312334
0.313044 0.321874 0.327980 0.330237 0.334854 0.336488
0.338017 0.340225 0.343390 0.354893 0.359116 0.365043
0.373094 0.393303 0.404240 0.425460 0.504155 0.599313
Minimum Search - Taking Simple RFO Step
Searching for Lamda that Minimizes Along All modes
Value Taken Lamda = 0.00000000
Step Taken. Stepsize is 0.008583
Maximum Tolerance Cnvgd?
Gradient 0.000079 0.000300 YES

Displacement 0.003247 0.001200 NO
Energy change -0.000001 0.000001 YES
****
** OPTIMIZATION CONVERGED **
****

- Entering anlman on Fri Mar 22 19:41:47 2019 -

Analysis of SCF Wavefunction
Ground-State Mulliken Net Atomic Charges
Atom Charge (a.u.)
----
1 C 0.158505
2 C -0.325434
3 N -0.431320
4 C -0.117652
5 H 0.122093
6 H 0.147263
7 H 0.182858
8 H 0.140852
9 C -0.013964
10 H 0.104972
11 C -0.456614
12 H 0.142143
13 H 0.168511
14 H 0.149565
15 C 0.148090
16 C -0.129487
17 C -0.189332
18 C -0.170730
19 C -0.132075
20 C -0.129032
21 H 0.120790
22 H 0.143652
23 H 0.126874
24 H 0.125990
25 H 0.125807
26 C 0.561900
27 N -0.467592
28 C 0.155473
29 C -0.060403
30 O -0.501467

31 H 0.150982
32 H 0.152170
33 C -0.443898
34 H 0.158550
35 H 0.151306
36 H 0.137360
37 C -0.442331
38 H 0.159276
39 H 0.138076
40 H 0.146078
41 C -0.480745
42 H 0.154248
43 H 0.168474
44 H 0.150219

Sum of atomic charges = 0.000000

Cartesian Multipole Moments

Charge (ESU x 10^10)
0.0000
Dipole Moment (Debye)
X -0.8407 Y -0.0258 Z -1.0184
Tot 1.3208
Quadrupole Moments (Debye-Ang)
XX -110.9613 XY 4.6558 YY -116.3802
XZ 3.4340 YZ -3.0704 ZZ -120.3907
Traceless Quadrupole Moments (Debye-Ang)
QXX 14.8483 QYY -1.4083 QZZ -13.4400
QXY 13.9674 QXZ 10.3021 QYZ -9.2111
Octapole Moments (Debye-Ang^2)
XXX -13.1943 XXY -22.0238 XYY -13.2770
YYY -8.5524 XXZ -9.1992 XYZ -12.3778
YYZ 2.5827 XZZ 1.6979 YZZ -9.3745
ZZZ 6.9716
Traceless Octapole Moments (Debye-Ang^2)
XXX 25.0458 YYY 231.2714 ZZZ 101.3786
XXY -210.5054 XXZ -139.0539 XYY -124.8349
XYZ -185.6670 XZZ 99.7891 YYZ 37.6753
YZZ -20.7660
Hexadecapole Moments (Debye-Ang^3)
XXXX -6732.1787 XXXY 104.0371 XXYY -1407.7708

XYYY 57.9348 YYYY -1267.4764 XXXZ -90.1114
XXYZ -28.5135 XYYZ -28.8121 YYYZ 74.8059
XXZZ -1335.6301 XYZZ 4.6894 YYZZ -368.1762
XZZZ -157.5979 YZZZ 69.0436 ZZZZ -978.5649
Traceless Hexadecapole Moments (Debye-Ang^3)
XXXX 9111.0274 XXXY 3424.1358 XXXZ 2981.7636
XXYY -5635.0090 XXYZ -4723.3007 XXZZ -3476.0184
XYYY -1416.6050 XYYZ 1122.5493 XYZZ -2007.5308
XZZZ -4104.3129 YYYY 4010.7147 YYYZ 2666.4597
YYZZ 1624.2943 YZZZ 2056.8410 ZZZZ 1851.7242

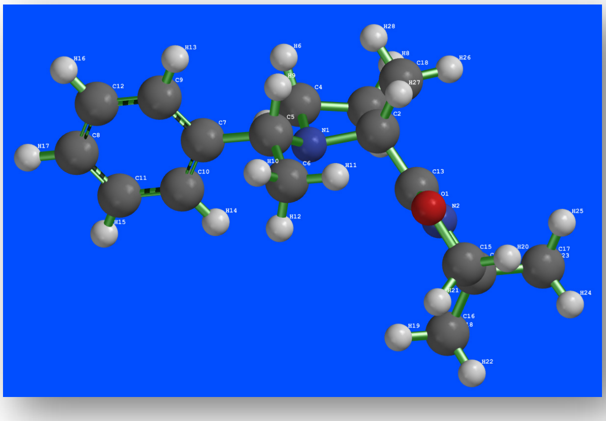	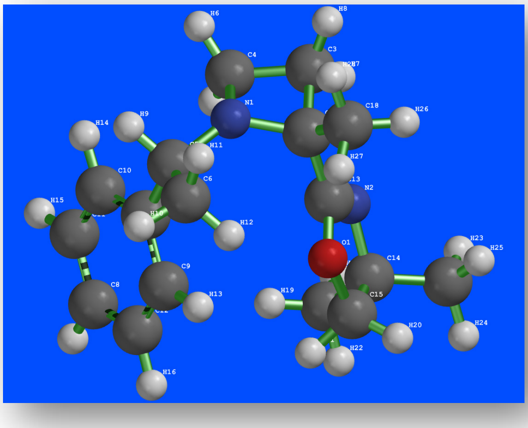
anti-(2*R*,1′*R*)-**2b**<br>**energy (kcal/mol): -531474.47**	*syn*-(2*R*,1′*R*)-**2b**<br>**energy: -531471.01**

Azetidine	**Energy (kcal/mol)**	**Erel. (kcal/mol)**
anti-(2*R*,1′*R*)-**2b**	**−531474.47**	0
syn-(2*R*,1′*R*)-**2b**	**−531471.01**	3,46

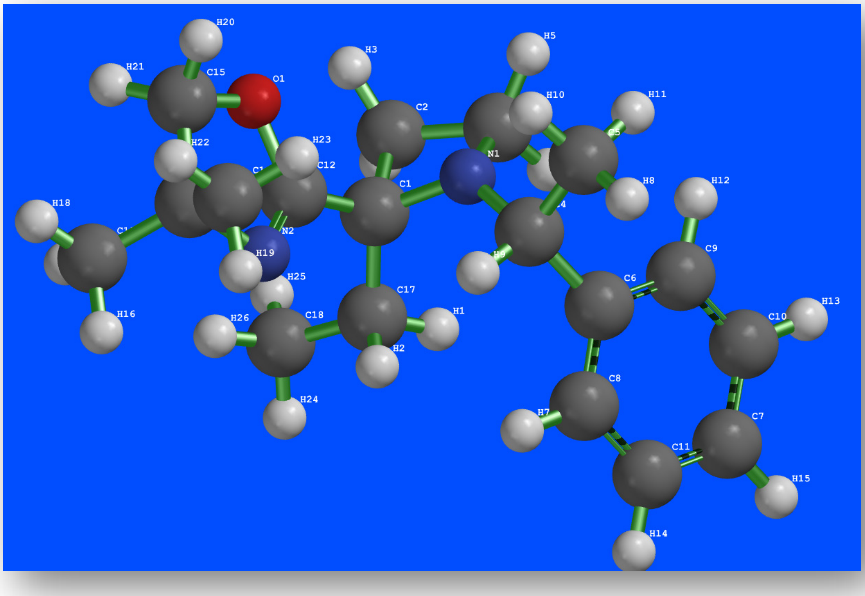

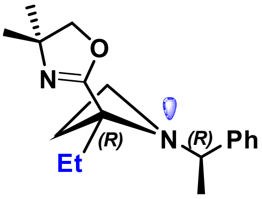

anti-(2R,1'R)-**2c**

** GEOMETRY OPTIMIZATION IN DELOCALIZED INTERNAL COORDINATES **

Job type: Geometry optimization.

Method: RB3LYP

Basis set: 6-31G(D)

Number of shells: 136

Number of basis functions: 367

Searching for a Minimum

Optimization Cycle: 28

Coordinates (Angstroms)

ATOM X Y Z

1 C -0.449231 -0.614580 -0.914754

2 C -0.234468 -0.724857 -2.455874

3 N 0.356042 0.636364 -0.969248

4 C 0.893110 0.323103 -2.298072

5 H 1.894580 -0.134784 -2.295124

6 H 0.897505 1.166361 -3.001613

7 H -1.086188 -0.363686 -3.031764

8 H 0.068224 -1.710764 -2.816033

9 C 1.070805 1.241040 0.156998

10 H 0.427495 1.055644 1.026178

11 C 1.137025 2.765007 -0.052061

12 H 1.615467 3.256960 0.802072

13 H 0.125207 3.163368 -0.175642

14 H 1.710555 3.023372 -0.950071

15 C 2.450798 0.658576 0.478108

16 C 4.982515 -0.423046 1.105469

17 C 2.653293 -0.075460 1.654918
18 C 3.550479 0.852264 -0.372097
19 C 4.801276 0.313058 -0.066398
20 C 3.902984 -0.610881 1.969719
21 H 1.819305 -0.226993 2.336939
22 H 3.436844 1.441815 -1.278085
23 H 5.636910 0.475136 -0.742806
24 H 4.032481 -1.172859 2.891394
25 H 5.957094 -0.839667 1.345881
26 C -1.843508 -0.281171 -0.422713
27 N -2.216258 -0.316272 0.797044
28 C -3.593224 0.224256 0.858935
29 C -3.935710 0.505815 -0.638072
30 O -2.726453 0.178204 -1.357151
31 H -4.176767 1.554615 -0.838300
32 H -4.743340 -0.125772 -1.023804
33 C -4.527877 -0.821503 1.480857
34 H -4.208113 -1.058518 2.500634
35 H -4.516216 -1.748860 0.897717
36 H -5.560075 -0.451470 1.519373
37 C -3.586833 1.511904 1.695824
38 H -3.229144 1.304384 2.709424
39 H -4.595136 1.938947 1.764398
40 H -2.924650 2.261088 1.248034
41 C 0.145692 -1.796151 -0.125808
42 H 0.160427 -1.537315 0.937186
43 H 1.187887 -1.923852 -0.441284
44 C -0.619223 -3.113654 -0.307509
45 H -0.126588 -3.920829 0.246130
46 H -0.667107 -3.418699 -1.359973
47 H -1.643205 -3.028047 0.069515
Point Group: c1 Number of degrees of freedom: 135
Energy is -886.265906210
Hessian Updated using BFGS Update
internal optimization with constraints (0)
135 Hessian modes will be used to form the next step
Hessian Eigenvalues:
0.000661 0.002772 0.003476 0.003621 0.003976 0.004319
0.005390 0.006816 0.008532 0.013543 0.017027 0.022090
0.022633 0.022994 0.024211 0.025363 0.025949 0.026727
0.027437 0.029100 0.030289 0.031325 0.033464 0.035736
0.037656 0.038605 0.040016 0.040920 0.042871 0.043279

0.043541 0.043672 0.043732 0.044001 0.044409 0.044553
0.044608 0.045862 0.046916 0.048025 0.050737 0.052069
0.057686 0.059418 0.062281 0.063013 0.068475 0.072966
0.077570 0.079475 0.082124 0.092207 0.096898 0.097939
0.103899 0.127084 0.127558 0.128442 0.130514 0.130893
0.131675 0.132033 0.134062 0.140107 0.142319 0.149031
0.149679 0.150812 0.153549 0.155935 0.156190 0.156971
0.158383 0.162413 0.163306 0.168508 0.174158 0.176478
0.188246 0.195261 0.205920 0.209390 0.224972 0.232948
0.240897 0.244368 0.250438 0.260630 0.268179 0.276482
0.277762 0.281058 0.285852 0.293578 0.296871 0.297762
0.299310 0.301489 0.302218 0.302280 0.302572 0.302709
0.303109 0.303203 0.303537 0.303995 0.304151 0.304245
0.304493 0.304966 0.305394 0.305709 0.306331 0.309564
0.309971 0.316243 0.318397 0.323207 0.329742 0.333829
0.335057 0.336748 0.338315 0.340780 0.343757 0.351089
0.353874 0.362213 0.365151 0.376490 0.382138 0.390636
0.474167 0.489776 0.596529
Minimum Search - Taking Simple RFO Step
Searching for Lamda that Minimizes Along All modes
Value Taken Lamda = 0.00000000
Step Taken. Stepsize is 0.010737
Maximum Tolerance Cnvgd?
Gradient 0.000197 0.000300 YES
Displacement 0.004654 0.001200 NO
Energy change -0.000001 0.000001 YES
****
** OPTIMIZATION CONVERGED **
****

- Entering anlman on Tue Mar 19 20:36:38 2019 -

Analysis of SCF Wavefunction
Ground-State Mulliken Net Atomic Charges
Atom Charge (a.u.)
----
1 C 0.167864
2 C -0.335755
3 N -0.416386
4 C -0.118692
5 H 0.130481
6 H 0.133253

7 H 0.172268
8 H 0.145549
9 C -0.063144
10 H 0.151297
11 C -0.438428
12 H 0.145403
13 H 0.149550
14 H 0.142866
15 C 0.182356
16 C -0.131905
17 C -0.196513
18 C -0.189685
19 C -0.131520
20 C -0.126841
21 H 0.131597
22 H 0.128333
23 H 0.126844
24 H 0.127495
25 H 0.126838
26 C 0.555790
27 N -0.472667
28 C 0.157436
29 C -0.060535
30 O -0.498720
31 H 0.152377
32 H 0.149457
33 C -0.444698
34 H 0.157959
35 H 0.147419
36 H 0.137806
37 C -0.445123
38 H 0.157382
39 H 0.137671
40 H 0.150303
41 C -0.278400
42 H 0.158253
43 H 0.141545
44 C -0.458196
45 H 0.144860
46 H 0.139612
47 H 0.157343

Sum of atomic charges = 0.000000

Cartesian Multipole Moments

Charge (ESU x 10^10)

0.0000

Dipole Moment (Debye)

X -0.7745 Y 0.0851 Z -0.3407

Tot 0.8504

Quadrupole Moments (Debye-Ang)

XX -114.3783 XY -3.2893 YY -127.2056

XZ -1.9610 YZ -0.6607 ZZ -125.6684

Traceless Quadrupole Moments (Debye-Ang)

QXX 24.1173 QYY -14.3645 QZZ -9.7528

QXY -9.8680 QXZ -5.8829 QYZ -1.9820

Octapole Moments (Debye-Ang^2)

XXX -36.6826 XXY -1.4041 XYY -9.6157

YYY -3.8026 XXZ -4.2671 XYZ -13.3991

YYZ -5.3561 XZZ 6.7954 YZZ -5.7445

ZZZ -4.6466

Traceless Octapole Moments (Debye-Ang^2)

XXX -194.7127 YYY 41.5221 ZZZ 58.7293

XXY 11.7917 XXZ -21.1968 XYY -25.7264

XYZ -200.9869 XZZ 220.4392 YYZ -37.5325

YZZ -53.3137

Hexadecapole Moments (Debye-Ang^3)

XXXX -6009.9966 XXXY -183.4738 XXYY -1342.9952

XYYY -95.4719 YYYY -1487.2989 XXXZ -41.2320

XXYZ -56.9372 XYYZ -7.7496 YYYZ -27.9997

XXZZ -1306.4599 XYZZ -43.8198 YYZZ -473.6545

XZZZ -26.4458 YZZZ -39.9230 ZZZZ -1378.2540

Traceless Hexadecapole Moments (Debye-Ang^3)

XXXX 12205.0930 XXXY -4740.3080 XXXZ -935.1303

XXYY -6928.7989 XXYZ -4119.3547 XXZZ -5276.2941

XYYY 4499.9003 XYYZ 317.7007 XYZZ 240.4077

XZZZ 617.4296 YYYY 5093.0706 YYYZ 2637.1947

YYZZ 1835.7283 YZZZ 1482.1599 ZZZZ 3440.5658

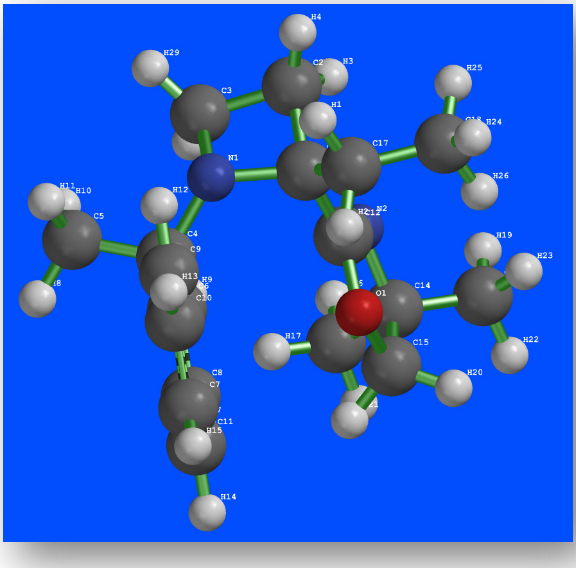

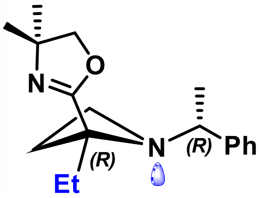

syn-(2*R*,1'*R*)-**2c**

** GEOMETRY OPTIMIZATION IN DELOCALIZED INTERNAL COORDINATES **

Job type: Geometry optimization.

Method: RB3LYP

Basis set: 6-31G(D)

Number of shells: 136

Number of basis functions: 367

Multiplicity: 1

Searching for a Minimum

Optimization Cycle: 46

Coordinates (Angstroms)

ATOM X Y Z

1 C -0.658370 1.360450 0.497671

2 C -1.276958 2.052758 1.743487

3 N 0.572448 1.098286 1.332597

4 C -0.189217 1.348962 2.577457

5 H -0.571957 0.433263 3.055073

6 H -2.310675 1.795327 1.970027

7 H -1.149252 3.137811 1.702525

8 C 1.375440 -0.119563 1.212323

9 H 0.735342 -1.022149 1.199572

10 C 2.294545 -0.236189 2.442583

11 H 2.920844 -1.129435 2.354911

12 H 1.714672 -0.316102 3.368483

13 H 2.950521 0.637743 2.517796

14 C 2.213662 -0.121555 -0.060903

15 C 3.871150 -0.176900 -2.334264

16 C 2.336283 -1.284172 -0.829348

17 C 2.934157 1.016134 -0.447861
18 C 3.753595 0.990615 -1.576309
19 C 3.159468 -1.316093 -1.956221
20 H 1.777026 -2.172796 -0.544259
21 H 2.832146 1.925221 0.137383
22 H 4.302108 1.884002 -1.864750
23 H 3.238433 -2.228448 -2.542239
24 H 4.508720 -0.196707 -3.214364
25 C -1.415790 0.093148 0.146002
26 N -2.394118 -0.414450 0.786976
27 C -2.779588 -1.669043 0.101988
28 C -1.766987 -1.759575 -1.082638
29 O -0.952862 -0.572718 -0.957585
30 H -2.245217 -1.741257 -2.067139
31 H -1.109487 -2.633107 -1.023090
32 C -2.630144 -2.843756 1.078819
33 H -3.283148 -2.704499 1.946401
34 H -1.598991 -2.918814 1.441102
35 H -2.895937 -3.791474 0.594225
36 C -4.230289 -1.553042 -0.387235
37 H -4.906425 -1.389078 0.457923
38 H -4.540496 -2.467881 -0.907110
39 H -4.341837 -0.710172 -1.078151
40 C -0.406952 2.232414 -0.739747
41 H 0.214825 1.680448 -1.451390
42 H 0.182057 3.092692 -0.401369
43 C -1.692485 2.705464 -1.428837
44 H -1.457026 3.375525 -2.262972
45 H -2.347507 3.251372 -0.739060
46 H -2.262023 1.861810 -1.834115
47 H 0.350768 1.941990 3.324308
Point Group: c1 Number of degrees of freedom: 135
Energy is -886.271761853
Hessian Updated using BFGS Update
internal optimization with constraints (0)
135 Hessian modes will be used to form the next step
Hessian Eigenvalues:
0.000399 0.003708 0.004131 0.004507 0.004795 0.005824
0.006764 0.007689 0.009890 0.013742 0.017688 0.021870
0.022738 0.023132 0.023676 0.024863 0.025810 0.026306
0.027880 0.028036 0.030140 0.030444 0.033628 0.035980
0.036662 0.038437 0.038888 0.040600 0.042638 0.043474

0.043920 0.044129 0.044513 0.044780 0.045653 0.046533
0.047101 0.047661 0.049677 0.050617 0.051691 0.054452
0.055026 0.061874 0.063133 0.067120 0.071330 0.079045
0.082256 0.084628 0.087499 0.093440 0.097881 0.106119
0.110838 0.126347 0.126990 0.127529 0.128126 0.130457
0.131620 0.132186 0.136795 0.140149 0.141505 0.145069
0.148684 0.150681 0.153620 0.156251 0.157847 0.158927
0.160235 0.163184 0.168246 0.173783 0.181389 0.191938
0.196772 0.202290 0.209376 0.211247 0.228192 0.234881
0.237576 0.246207 0.253952 0.257956 0.266209 0.269816
0.271454 0.277117 0.284502 0.285949 0.290452 0.292849
0.297797 0.299268 0.300377 0.300522 0.302105 0.302365
0.302535 0.303080 0.303699 0.304041 0.304420 0.305308
0.305584 0.305815 0.306635 0.306778 0.308263 0.310602
0.315250 0.318721 0.320716 0.325239 0.330688 0.333852
0.335407 0.337253 0.338710 0.340510 0.344089 0.354575
0.357653 0.370140 0.383580 0.399420 0.414478 0.427427
0.526938 0.653998 0.887840
Minimum Search - Taking Simple RFO Step
Searching for Lamda that Minimizes Along All modes
Value Taken Lamda = -0.00000210
Step Taken. Stepsize is 0.055614
Maximum Tolerance Cnvgd?
Gradient 0.000266 0.000300 YES
Displacement 0.026338 0.001200 NO
Energy change -0.000001 0.000001 YES
****
** OPTIMIZATION CONVERGED **
****

- Entering anlman on Wed Mar 20 22:48:43 2019 -

Analysis of SCF Wavefunction
Ground-State Mulliken Net Atomic Charges
Atom Charge (a.u.)
----
1 C 0.176175
2 C -0.323262
3 N -0.429124
4 C -0.123492
5 H 0.136550
6 H 0.174509

7 H 0.141986
8 C -0.035532
9 H 0.109772
10 C -0.450086
11 H 0.151458
12 H 0.148523
13 H 0.155344
14 C 0.173262
15 C -0.130219
16 C -0.176895
17 C -0.174733
18 C -0.132127
19 C -0.131398
20 H 0.115611
21 H 0.137615
22 H 0.125047
23 H 0.123118
24 H 0.124239
25 C 0.531335
26 N -0.472202
27 C 0.156540
28 C -0.062745
29 O -0.496639
30 H 0.153051
31 H 0.150136
32 C -0.446000
33 H 0.159272
34 H 0.147273
35 H 0.138902
36 C -0.443886
37 H 0.158983
38 H 0.138681
39 H 0.148710
40 C -0.271717
41 H 0.168063
42 H 0.134683
43 C -0.458353
44 H 0.146419
45 H 0.146292
46 H 0.149723
47 H 0.137140

Sum of atomic charges = 0.000000

Cartesian Multipole Moments

Charge (ESU x 10^10)

0.0000

Dipole Moment (Debye)

X -0.6670 Y -1.2233 Z -0.4777

Tot 1.4729

Quadrupole Moments (Debye-Ang)

XX -127.7166 XY 3.7834 YY -119.0426

XZ 1.2629 YZ 1.9915 ZZ -124.9218

Traceless Quadrupole Moments (Debye-Ang)

QXX -11.4688 QYY 14.5532 QZZ -3.0844

QXY 11.3501 QXZ 3.7886 QYZ 5.9746

Octapole Moments (Debye-Ang^2)

XXX -19.0785 XXY -6.7628 XYY -7.5453

YYY -17.3962 XXZ -26.2711 XYZ -3.3231

YYZ -10.3076 XZZ 7.9457 YZZ -5.8092

ZZZ -13.4017

Traceless Octapole Moments (Debye-Ang^2)

XXX -118.0740 YYY 8.7708 ZZZ 248.7976

XXY -11.5380 XXZ -244.1247 XYY -57.1454

XYZ -49.8469 XZZ 175.2194 YYZ -4.6729

YZZ 2.7672

Hexadecapole Moments (Debye-Ang^3)

XXXX -4568.2806 XXXY -173.8130 XXYY -1044.8291

XYYY -235.1496 YYYY -1974.7753 XXXZ 227.6215

XXYZ -17.8913 XYYZ 82.1120 YYYZ -39.8524

XXZZ -987.2037 XYZZ -86.5510 YYZZ -609.2240

XZZZ 255.4180 YZZZ -43.0383 ZZZZ -1718.4115

Traceless Hexadecapole Moments (Debye-Ang^3)

XXXX -7537.0899 XXXY 4047.7470 XXXZ -1531.5607

XXYY 3098.1317 XXYZ -367.4291 XXZZ 4438.9582

XYYY -2392.6001 XYYZ 144.4866 XYZZ -1655.1469

XZZZ 1387.0741 YYYY -2652.6795 YYYZ 348.9651

YYZZ -445.4522 YZZZ 18.4639 ZZZZ -3993.5059

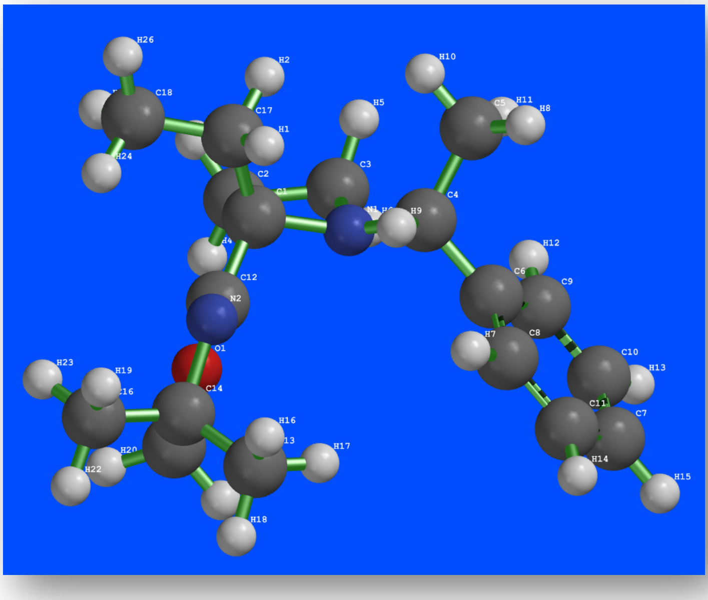

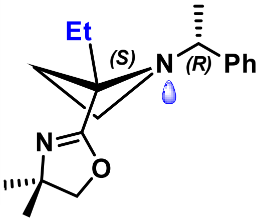

anti-(2*S*,1'*R*)-**2c**

** GEOMETRY OPTIMIZATION IN DELOCALIZED INTERNAL COORDINATES **

Job type: Geometry optimization.

Method: RB3LYP

Basis set: 6-31G(D)

Number of shells: 136

Number of basis functions: 367

Multiplicity: 1

Searching for a Minimum

Optimization Cycle: 16

Coordinates (Angstroms)

ATOM X Y Z

1 C 1.000449 1.474882 0.205994

2 C 0.893736 2.366700 1.482326

3 N -0.474100 1.317321 0.283713

4 C -0.631398 2.431515 1.221766

5 H -1.286997 2.224038 2.077635

6 H -0.948153 3.383704 0.764850

7 H 1.436314 3.313706 1.435116

8 H 1.153533 1.834681 2.397284

9 C -1.339273 0.971104 -0.845089

10 H -0.679056 0.489988 -1.576009

11 C -2.012951 2.175093 -1.535401

12 H -2.602144 1.834880 -2.394139

13 H -1.271722 2.897041 -1.894371

14 H -2.696453 2.698053 -0.857293

15 C -2.374120 -0.075656 -0.429780

16 C -4.254587 -2.029935 0.337614
17 C -2.347545 -1.358109 -0.989876
18 C -3.365970 0.214198 0.517825
19 C -4.294621 -0.752812 0.902023
20 C -3.278134 -2.329356 -0.612181
21 H -1.586310 -1.600417 -1.728745
22 H -3.412236 1.203873 0.965267
23 H -5.052980 -0.508501 1.641830
24 H -3.238242 -3.318935 -1.060913
25 H -4.979362 -2.783172 0.635520
26 C 1.651154 0.114533 0.356348
27 N 2.047743 -0.621499 -0.606837
28 C 2.475979 -1.917015 -0.031302
29 C 2.225570 -1.730242 1.499470
30 O 1.714944 -0.384451 1.624216
31 H 3.136901 -1.805402 2.101835
32 H 1.473275 -2.416705 1.901267
33 C 1.613243 -3.040536 -0.622977
34 H 1.737896 -3.079711 -1.710208
35 H 0.552804 -2.871286 -0.406537
36 H 1.900239 -4.014922 -0.207977
37 C 3.959709 -2.147539 -0.350834
38 H 4.114373 -2.170655 -1.434311
39 H 4.307876 -3.099640 0.068573
40 H 4.576667 -1.342653 0.063794
41 C 1.549678 2.225457 -1.023349
42 H 0.994095 3.167925 -1.113856
43 H 1.342593 1.637557 -1.924246
44 C 3.052497 2.524455 -0.955662
45 H 3.633768 1.598168 -0.965939
46 H 3.314393 3.084274 -0.049661
47 H 3.363049 3.126745 -1.816782
Point Group: c1 Number of degrees of freedom: 135
Energy is -886.264153144
Hessian Updated using BFGS Update
internal optimization with constraints (0)
135 Hessian modes will be used to form the next step
Hessian Eigenvalues:
0.000637 0.001719 0.003076 0.003674 0.003866 0.004078
0.004111 0.005967 0.008637 0.009945 0.013037 0.021437
0.022354 0.022668 0.022909 0.023612 0.025666 0.026244
0.026542 0.028225 0.029859 0.030136 0.034479 0.036326

0.038322 0.039126 0.040370 0.041267 0.042946 0.043119
0.043866 0.044012 0.044407 0.044667 0.045063 0.045330
0.045697 0.046747 0.046997 0.049034 0.049528 0.052693
0.053483 0.055118 0.059704 0.066310 0.071177 0.074766
0.075628 0.083734 0.090484 0.094052 0.098559 0.103233
0.116707 0.125835 0.125993 0.127230 0.128945 0.130030
0.131351 0.132043 0.133728 0.139383 0.140622 0.148480
0.149053 0.151341 0.153992 0.156490 0.156973 0.159539
0.159699 0.165812 0.169868 0.171671 0.173610 0.182339
0.191257 0.194409 0.212559 0.213797 0.216582 0.225799
0.229995 0.231258 0.244477 0.256198 0.257843 0.264898
0.265984 0.277156 0.282953 0.289231 0.292504 0.295909
0.297522 0.299818 0.300434 0.300921 0.300972 0.302006
0.302775 0.302962 0.303151 0.303347 0.303604 0.304612
0.304811 0.305001 0.305325 0.305629 0.305815 0.306117
0.306441 0.308786 0.314144 0.316423 0.322846 0.323745
0.329600 0.331024 0.332512 0.336239 0.338007 0.341823
0.352813 0.356573 0.359110 0.365720 0.381642 0.398291
0.407663 0.424991 0.664607
Minimum Search - Taking Simple RFO Step
Searching for Lamda that Minimizes Along All modes
Value Taken Lamda = 0.00000000
Step Taken. Stepsize is 0.013179
Maximum Tolerance Cnvgd?
Gradient 0.000069 0.000300 YES
Displacement 0.003470 0.001200 NO
Energy change 0.000000 0.000001 YES
****
** OPTIMIZATION CONVERGED **
****

- Entering anlman on Thu Jan 20 17:36:49 2022 -

Analysis of SCF Wavefunction
Ground-State Mulliken Net Atomic Charges
Atom Charge (a.u.)
----
1 C 0.163794
2 C -0.337406
3 N -0.415896
4 C -0.111507
5 H 0.137486

6 H 0.120628
7 H 0.145091
8 H 0.174307
9 C -0.064634
10 H 0.151048
11 C -0.460452
12 H 0.152749
13 H 0.147858
14 H 0.147191
15 C 0.204380
16 C -0.131112
17 C -0.201631
18 C -0.177582
19 C -0.130298
20 C -0.128680
21 H 0.130082
22 H 0.124033
23 H 0.125369
24 H 0.125285
25 H 0.124903
26 C 0.563202
27 N -0.475068
28 C 0.157150
29 C -0.060408
30 O -0.498641
31 H 0.149052
32 H 0.155377
33 C -0.451949
34 H 0.154641
35 H 0.157525
36 H 0.137081
37 C -0.443905
38 H 0.157466
39 H 0.137932
40 H 0.146773
41 C -0.277522
42 H 0.129191
43 H 0.158064
44 C -0.459112
45 H 0.165679
46 H 0.139350
47 H 0.143119

Sum of atomic charges = 0.000000

Cartesian Multipole Moments

Charge (ESU x 10^10)

0.0000

Dipole Moment (Debye)

X 0.4206 Y -0.1483 Z 0.5280

Tot 0.6911

Quadrupole Moments (Debye-Ang)

XX -124.5620 XY -2.4366 YY -116.9092

XZ 0.1543 YZ -0.2444 ZZ -127.6590

Traceless Quadrupole Moments (Debye-Ang)

QXX -4.5557 QYY 18.4026 QZZ -13.8469

QXY -7.3099 QXZ 0.4628 QYZ -0.7331

Octapole Moments (Debye-Ang^2)

XXX 10.7268 XXY -16.1246 XYY 5.1367

YYY -11.0563 XXZ 29.3532 XYZ -6.1717

YYZ 5.1419 XZZ 2.0093 YZZ -0.4712

ZZZ 4.0858

Traceless Octapole Moments (Debye-Ang^2)

XXX 0.0462 YYY 83.0241 ZZZ -285.9410

XXY -158.9123 XXZ 324.5551 XYY 23.4323

XYZ -92.5762 XZZ -23.4785 YYZ -38.6142

YZZ 75.8882

Hexadecapole Moments (Debye-Ang^3)

XXXX -4985.7697 XXXY -41.5709 XXYY -1243.7840

XYYY -37.1171 YYYY -2819.5767 XXXZ -89.6218

XXYZ -12.3136 XYYZ 8.9642 YYYZ 17.3510

XXZZ -982.0378 XYZZ -29.5289 YYZZ -618.6972

XZZZ -10.4870 YZZZ 13.3396 ZZZZ -911.9564

Traceless Hexadecapole Moments (Debye-Ang^3)

XXXX -4119.6534 XXXY 504.8146 XXXZ -5308.7834

XXYY 4588.4003 XXYZ -1563.4856 XXZZ -468.7470

XYYY 972.4675 XYYZ 2308.4126 XYZZ -1477.2821

XZZZ 3000.3708 YYYY -4327.4133 YYYZ 1010.1728

YYZZ -260.9870 YZZZ 553.3128 ZZZZ 729.7340

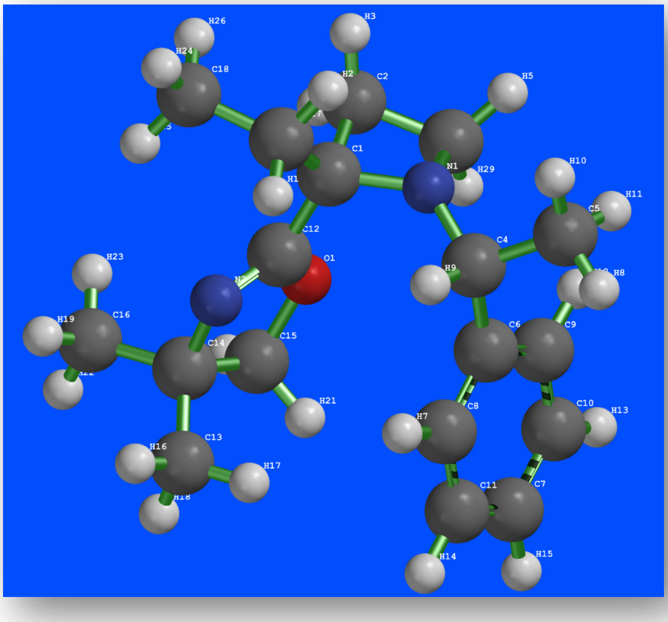

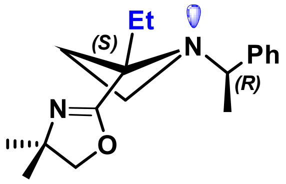

syn-(2S,1'R)-**2c**

** GEOMETRY OPTIMIZATION IN DELOCALIZED INTERNAL COORDINATES **

Job type: Geometry optimization.

Method: RB3LYP

Basis set: 6-31G(D)

Number of shells: 136

Number of basis functions: 367

Multiplicity: 1

Searching for a Minimum

Optimization Cycle: 40

Coordinates (Angstroms)

ATOM X Y Z

1 C 0.771352 1.736083 0.072961

2 C 0.507484 2.514424 1.406083

3 N -0.677861 1.909695 -0.213329

4 C -1.001863 2.273181 1.171849

5 H -1.671888 3.138113 1.270282

6 H 0.796013 3.564570 1.314056

7 H 0.934356 2.086843 2.313845

8 C -1.490583 1.135339 -1.148388

9 H -0.836793 0.961888 -2.012701

10 C -2.666110 2.009478 -1.624542

11 H -3.255530 1.488225 -2.386547

12 H -2.287814 2.948063 -2.041404

13 H -3.338203 2.252239 -0.793746

14 C -1.989915 -0.238045 -0.686447

15 C -2.950560 -2.783535 0.067501
16 C -1.583095 -1.394528 -1.364850
17 C -2.904086 -0.381308 0.369162
18 C -3.371004 -1.639639 0.749596
19 C -2.059125 -2.655839 -0.998582
20 H -0.890646 -1.303911 -2.198968
21 H -3.269943 0.497083 0.892580
22 H -4.074773 -1.725923 1.573724
23 H -1.738377 -3.535930 -1.550686
24 H -3.323111 -3.762513 0.357569
25 C 1.201929 0.302807 0.332176
26 N 2.037542 -0.373371 -0.354795
27 C 2.204247 -1.684813 0.317854
28 C 1.076380 -1.674401 1.390934
29 O 0.629534 -0.298786 1.421298
30 H 1.418620 -1.941043 2.394902
31 H 0.223895 -2.303423 1.115381
32 C 2.042705 -2.825566 -0.691916
33 H 2.798356 -2.746152 -1.480517
34 H 1.054197 -2.792311 -1.159687
35 H 2.159716 -3.800258 -0.201943
36 C 3.601899 -1.721317 0.957769
37 H 4.372709 -1.576951 0.194022
38 H 3.779591 -2.683358 1.454452
39 H 3.707994 -0.924547 1.702634
40 C 1.681206 2.416244 -0.961604
41 H 1.191158 3.361199 -1.223310
42 H 1.711491 1.800307 -1.868031
43 C 3.113777 2.681950 -0.482771
44 H 3.676192 3.226538 -1.249774
45 H 3.640681 1.746317 -0.278729
46 H 3.126386 3.293138 0.428409
47 H -1.383510 1.451226 1.793079
Point Group: c1 Number of degrees of freedom: 135
Energy is -886.267126354
Hessian Updated using BFGS Update
internal optimization with constraints (0)
135 Hessian modes will be used to form the next step
Hessian Eigenvalues:
0.002361 0.002964 0.003061 0.003828 0.004114 0.004493
0.005710 0.008169 0.009589 0.013012 0.016843 0.022457
0.022680 0.023909 0.024808 0.025227 0.026249 0.026684

0.027776 0.029030 0.029637 0.031504 0.035472 0.036379
0.040082 0.040378 0.041990 0.043076 0.043192 0.043386
0.043652 0.043979 0.044318 0.044568 0.044750 0.044990
0.046400 0.046930 0.048223 0.049414 0.051187 0.054044
0.054823 0.060583 0.064047 0.066817 0.068124 0.077634
0.079141 0.082114 0.085006 0.094038 0.096296 0.099453
0.107254 0.127938 0.128555 0.128767 0.129231 0.130560
0.131775 0.132261 0.133522 0.139326 0.143684 0.148917
0.149549 0.150902 0.155043 0.155653 0.157430 0.158505
0.159310 0.162742 0.166695 0.169386 0.175318 0.189953
0.198846 0.203488 0.206333 0.210320 0.225497 0.228934
0.242314 0.249253 0.250597 0.254665 0.261647 0.265759
0.273424 0.278434 0.289165 0.296883 0.297857 0.300110
0.300827 0.302091 0.302340 0.302656 0.302751 0.303044
0.303433 0.303728 0.304180 0.304489 0.304826 0.305206
0.305495 0.306435 0.306954 0.307958 0.308800 0.311804
0.312128 0.315426 0.317381 0.322442 0.330767 0.333423
0.336441 0.337366 0.340606 0.343190 0.346539 0.350188
0.356825 0.360467 0.369254 0.386386 0.394573 0.424773
0.447188 0.580036 0.633561
Minimum Search - Taking Simple RFO Step
Searching for Lamda that Minimizes Along All modes
Value Taken Lamda = 0.00000000
Step Taken. Stepsize is 0.003315
Maximum Tolerance Cnvgd?
Gradient 0.000097 0.000300 YES
Displacement 0.000900 0.001200 YES
Energy change 0.000000 0.000001 YES
****
** OPTIMIZATION CONVERGED **
****

- Entering anlman on Wed Mar 20 15:54:14 2019 -

Analysis of SCF Wavefunction
Ground-State Mulliken Net Atomic Charges
Atom Charge (a.u.)
----
1 C 0.160772
2 C -0.330575
3 N -0.410034
4 C -0.112798

5 H 0.128068
6 H 0.149205
7 H 0.157883
8 C -0.065555
9 H 0.141307
10 C -0.439500
11 H 0.147357
12 H 0.152096
13 H 0.144868
14 C 0.188224
15 C -0.134860
16 C -0.199974
17 C -0.181833
18 C -0.131224
19 C -0.132389
20 H 0.133386
21 H 0.130003
22 H 0.128199
23 H 0.128605
24 H 0.127526
25 C 0.564218
26 N -0.479816
27 C 0.156955
28 C -0.063658
29 O -0.504506
30 H 0.147768
31 H 0.164421
32 C -0.455735
33 H 0.155457
34 H 0.152625
35 H 0.136581
36 C -0.441464
37 H 0.158132
38 H 0.137461
39 H 0.147306
40 C -0.264087
41 H 0.131881
42 H 0.149112
43 C -0.458296
44 H 0.140789
45 H 0.166755
46 H 0.136011

47 H 0.143336

Sum of atomic charges = 0.000000

Cartesian Multipole Moments

Charge (ESU x 10^10)

0.0000

Dipole Moment (Debye)

X -0.4522 Y -1.1244 Z 1.0160

Tot 1.5815

Quadrupole Moments (Debye-Ang)

XX -127.6577 XY -1.4834 YY -119.6775

XZ 0.0299 YZ -0.2989 ZZ -125.3882

Traceless Quadrupole Moments (Debye-Ang)

QXX -10.2497 QYY 13.6908 QZZ -3.4411

QXY -4.4503 QXZ 0.0896 QYZ -0.8968

Octapole Moments (Debye-Ang^2)

XXX 1.6482 XXY -5.0626 XYY 1.0933

YYY -17.8273 XXZ 23.6856 XYZ 0.2139

YYZ 9.4862 XZZ 2.2787 YZZ -0.8108

ZZZ 6.6663

Traceless Octapole Moments (Debye-Ang^2)

XXX -20.4582 YYY -54.1036 ZZZ -258.5481

XXY -4.8368 XXZ 235.7695 XYY 1.3390

XYZ 3.2090 XZZ 19.1193 YYZ 22.7786

YZZ 58.9404

Hexadecapole Moments (Debye-Ang^3)

XXXX -3824.4718 XXXY -152.5380 XXYY -1112.4014

XYYY -92.6791 YYYY -3024.1248 XXXZ -231.6760

XXYZ -9.3224 XYYZ -61.9017 YYYZ 54.5635

XXZZ -780.5678 XYZZ -45.7753 YYZZ -661.3409

XZZZ -164.6956 YZZZ 40.6435 ZZZZ -944.4176

Traceless Hexadecapole Moments (Debye-Ang^3)

XXXX -3114.5546 XXXY -2921.8331 XXXZ -3703.6810

XXYY 2222.5698 XXYZ -2257.4714 XXZZ 891.9848

XYYY 3363.3522 XYYZ 374.4230 XYZZ -441.5191

XZZZ 3329.2580 YYYY -1839.7717 YYYZ 1893.3194

YYZZ -382.7981 YZZZ 364.1520 ZZZZ -509.1867

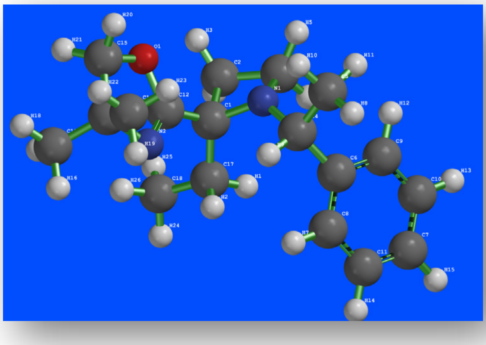	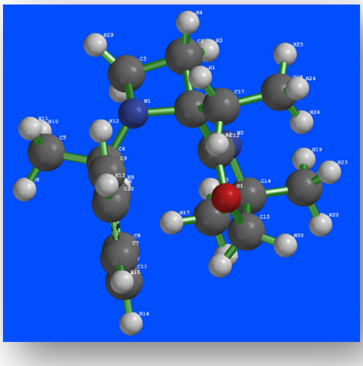
anti-(2*R*,1′*R*)-**2c**<br>**E (kcal/mol): −556140.72**	*syn*-(2*R*,1′*R*)-**2c**<br>**E (kcal/mol): −556144.39**
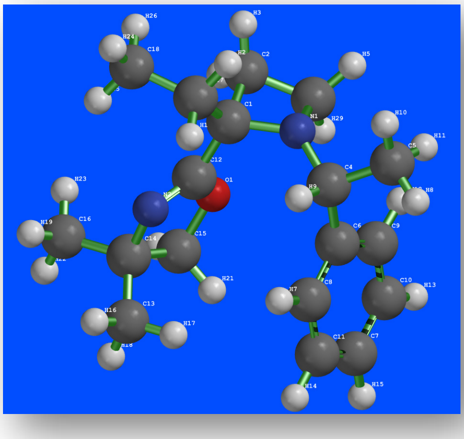	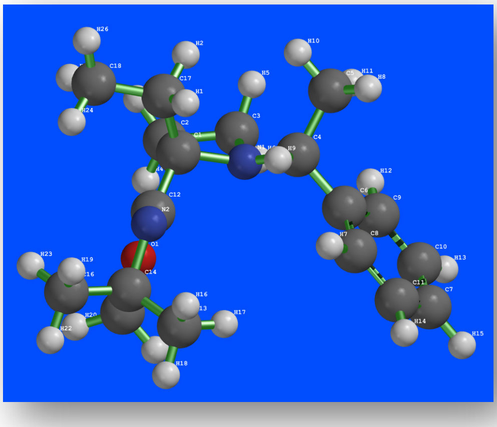
syn-(2*S*,1′*R*)-**2c**<br>**E (kcal/mol): −556141.48**	*anti*-(2*S*,1′*R*)-**2c**<br>**E (kcal/mol): −556139.62**

**Azetidine**	**E (kcal/mol)**	**Erel (kcal/mol)**
anti-(2*R*,1′*R*)-**2c**	−556140,72	3,67
syn-(2*R*,1′*R*)-**2c**	**−556144,39**	**0**
syn-(2*S*,1′*R*)-**2c**	−556141,48	2.91
anti-(2*S*,1′*R*)-**2c**	−556139,62	4.77

Density functional theory (DFT) [35] calculations were performed using Gaussian 09 (revision E.01) [36] and the Gaussview [37] was used to generate input geometries and visualize output structures. Regarding geometry optimizations and frequency calculations for neutral and lithiated oxazolinylazetidines, different functionals (B3LYP [38–40], CAM-B3LYP [41], B3PW91 [38,42], BVP86 [42], MPW1PW91 [43], B97XD [44], M06-2X [45],and PBEPBE [46]) implemented in the Gaussian program were used with the 6-31+G(d,p) basis set.

To model the solvation effect, the calculations were carried out in tetrahydrofuran (THF) by applying the most commonly used integral equation formalism (IEF) version of

polarized continuum model (PCM) [47,48] and the conductor-like polarized continuum model (CPCM) [49,50]. All stationary points were characterized as minima and thermal corrections were computed from unscaled frequencies, assuming a standard state of 298.15 K and 1 atm.

Computed wavelength (cm−1) for C=N bond in neutral oxazolinylazetidine

(BVP86/6-31+G(d,p), IEF-PCM, solvent: THF)

No.	structure	IR spectrum
**1.**	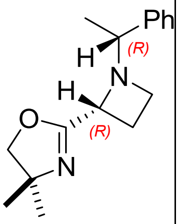	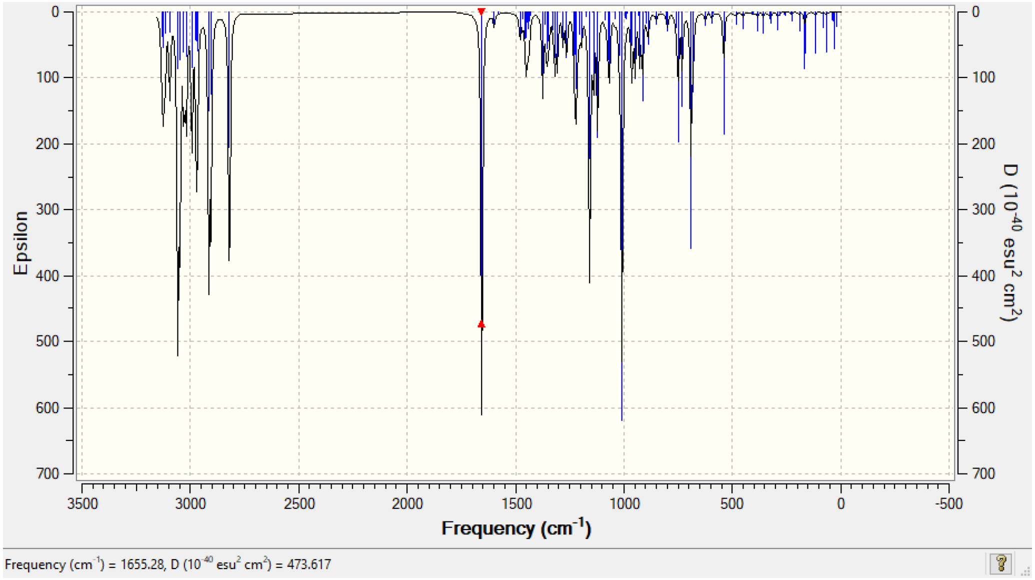
**2.**	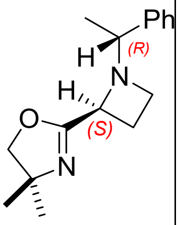	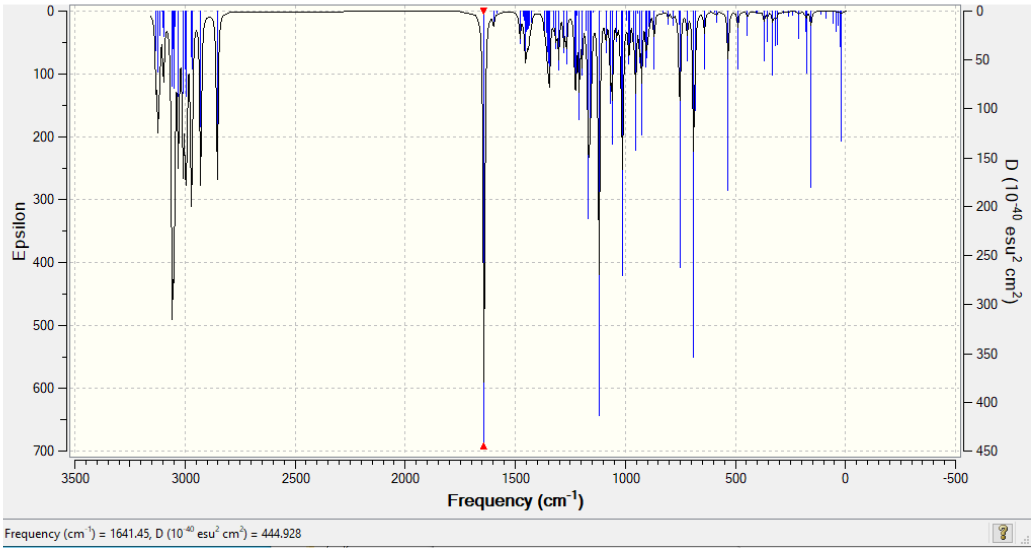

Optimized Structure and Computed Energies [values are in Hartree]

(6-31+G(d,p), IEF-PCM, solvent: THF)

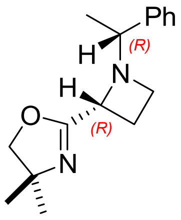

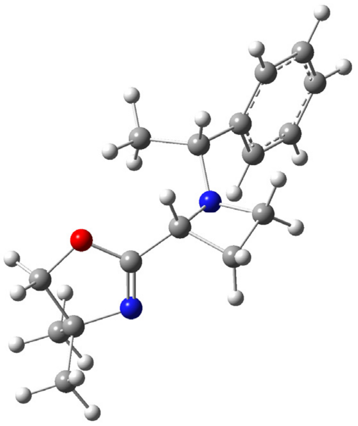

Method	Total Electronic Energy	Sum of Electronic and Zero-Point Energies	Sum of Electronic and Thermal Enthalpies	Gibbs Free Energy
BVP86	-807.7336182	-807.389779	-807.370076	-807.438611
B3LYP	-807.7080247	-807.353964	-807.334760	-807.402408
CAM-B3LYP	-807.2678996	-806.909373	-806.890390	-806.957662
B3PW91	-807.4049945	-807.049898	-807.030739	-807.098044
MPW1PW91	-807.521483	-807.164084	-807.144960	-807.212848
B97XD	-807.4631075	-807.104062	-807.085320	-807.151268
M0-62X	-807.3443184	-806.986767	-806.967786	-807.034883
PBEPBE	-806.6718202	-806.327470	-806.307764	-806.376601

Cartesian Coordinates (BVP86/6-31+G(d,p), IEF-PCM, solvent: THF)

C	0.57124500	2.37099200	-1.05791500
C	-0.91352500	1.93852800	-1.00274900
C	0.78067200	1.42571700	0.15642800
H	1.09227600	1.99817900	-1.95112300
H	0.80050700	3.43328100	-0.89634200
H	-1.43174600	1.70681300	-1.94730600
H	-1.53073600	2.64951900	-0.41328800
N	-0.50663200	0.74170700	-0.20488800
C	2.02384300	0.57396500	0.19547800
C	3.79045200	-0.72163000	-0.28585400
C	3.76434700	-0.42154800	1.25167500
N	2.59460500	0.00099100	-0.80390000
O	2.59067600	0.43176100	1.44490800

C	-1.40041300	0.33474700	0.89160700
H	-1.59214800	1.20482300	1.56714300
C	-2.74607000	-0.10943000	0.32426400
C	-3.94846000	0.34035600	0.90428500
C	-2.81309600	-1.00547100	-0.76343400
C	-5.19214800	-0.10034100	0.41921300
H	-3.91013500	1.04316800	1.74442800
C	-4.05347600	-1.44333700	-1.25427800
H	-1.88353300	-1.34711300	-1.22951300
C	-5.24815800	-0.99422600	-0.66305200
H	-6.11572700	0.26038900	0.88298100
H	-4.08853100	-2.13511700	-2.10203700
H	-6.21475100	-1.33542800	-1.04654000
C	-0.74977800	-0.78317900	1.72807600
H	-1.42141600	-1.08037600	2.54944700
H	-0.55280400	-1.66821300	1.10092300
H	0.20236600	-0.44660300	2.17001300
H	3.62725900	-1.32332600	1.87021600
H	4.64591300	0.13728400	1.60479500
C	5.05466300	-0.15665900	-0.95919100
C	3.64417900	-2.22591000	-0.57919300
H	5.15561900	0.92337000	-0.76182600
H	5.95566100	-0.66605600	-0.57626100
H	5.00861800	-0.30645200	-2.05012400
H	2.73145600	-2.63030500	-0.11180500
H	3.58366300	-2.40132200	-1.66561300
H	4.51268100	-2.78136100	-0.18535400
H	0.69862900	1.97642200	1.11728700

Optimized Structure and Computed Energies [values are in Hartree]

(6-31+G(d,p), IEF-PCM, solvent: THF)

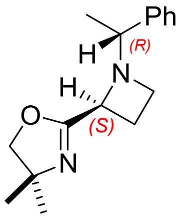

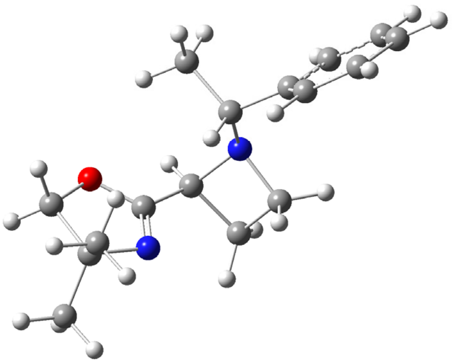

Method	Total Electronic Energy	Sum of Electronic and Zero-Point Energies	Sum of Electronic and Thermal Enthalpies	Gibbs Free Energy
BVP86	-807.7305657	-807.386845	-807.367048	-807.436050
B3LYP	-807.7044855	-807.350463	-807.331194	-807.399432
CAM-B3LYP	-807.2646018	-806.906096	-806.887104	-806.954356
B3PW91	-807.4010834	-807.046057	-807.026801	-807.094792
MPW1PW91	-807.5183618	-807.160932	-807.141842	-807.209046
B97XD	-807.4598274	-807.100824	-807.082083	-807.147893
M0-62X	-807.3433066	-806.985799	-806.966914	-807.033150
PBEPBE	-806.6688984	-806.324537	-806.304817	-806.373406

Cartesian Coordinates (BVP86/6-31+G(d,p), IEF-PCM, solvent: THF)

C	-0.64432500	-2.44001300	1.25222900
C	0.58830700	-1.51382800	1.40227000
C	-0.85172200	-1.80051000	-0.15303700
H	-0.38183700	-3.50565200	1.18301900
H	-1.46285200	-2.29248400	1.96869600
H	1.52611500	-1.94825800	1.78714000
H	0.34913800	-0.59282100	1.97121900
N	0.57369900	-1.31077000	-0.07555700
C	0.96802300	-0.00421000	-0.62249000
H	0.32810600	0.80855500	-0.20586700
C	2.40799800	0.30139200	-0.21625100
C	2.74779600	1.56030300	0.31643300
C	3.43150100	-0.65205400	-0.40152500
C	4.07909300	1.86831000	0.64736600
H	1.96089800	2.30644700	0.47431000
C	4.76104100	-0.35111800	-0.06622200
H	3.17460100	-1.63780400	-0.80230100
C	5.09059300	0.91234100	0.45716400
H	4.32322500	2.85240900	1.05989300
H	5.54233300	-1.10389400	-0.21260500
H	6.12711100	1.14714400	0.71851100
C	0.81375100	-0.01076900	-2.15691700
H	1.09998500	0.96945400	-2.57144200
H	1.46318700	-0.78133100	-2.60446200
H	-0.22852000	-0.21587300	-2.45070300
C	-1.92988000	-0.73792600	-0.18074800
C	-3.50016100	0.51488600	-1.23320700
C	-3.39442600	0.90143900	0.27970900
H	-4.49803300	0.15342400	-1.52740900

H	-3.18714900	1.32637700	-1.91092500
N	-2.29882600	0.02710100	0.78744700
O	-2.55827100	-0.59286400	-1.40118800
C	-3.00944400	2.37885700	0.47116200
C	-4.68901300	0.57271300	1.04779000
H	-2.07007100	2.61073900	-0.05704500
H	-3.80215300	3.03895900	0.07898800
H	-2.86978000	2.60363100	1.54108000
H	-4.96548600	-0.48631400	0.91472000
H	-4.55492600	0.76325900	2.12500300
H	-5.52246900	1.19839000	0.68484400
H	-1.02415200	-2.48465200	-1.00074100

Computed wavelength (cm^{-1}) for C=N bond in lithiated oxazolinylazetidine [*anti*-(2*R*,1′*R*)-1-Li and *syn*-(2*S*,1′*R*)-1-Li] (B97XD/6-31+G(d,p), IEF-PCM, solvent: THF)

No.	structure	IR spectrum
1.	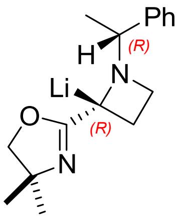	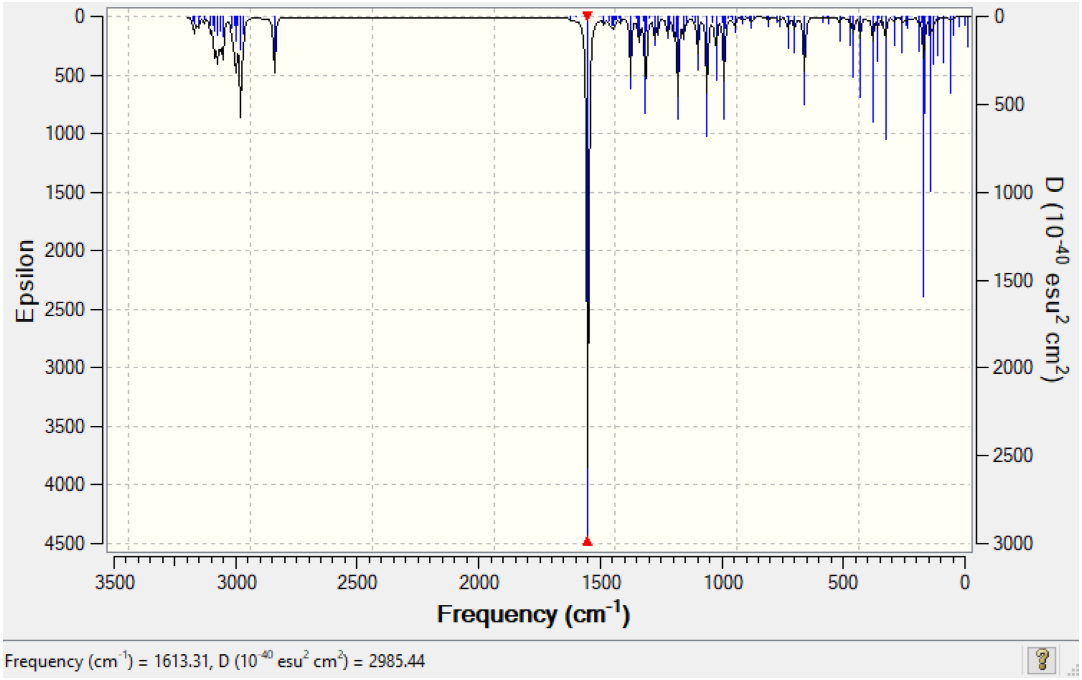

**2.**	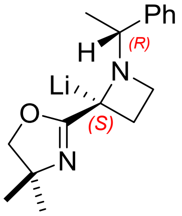	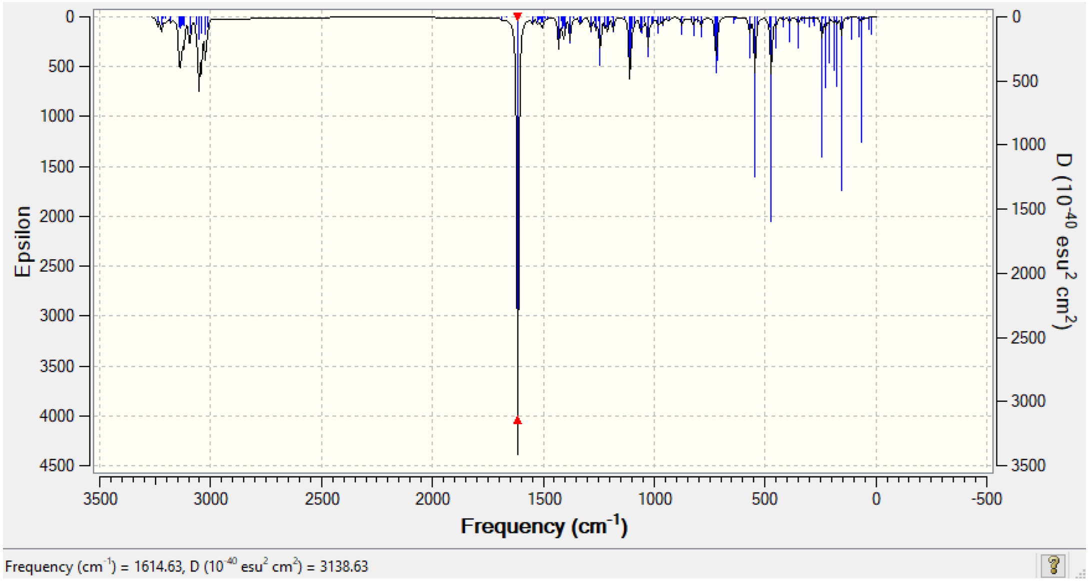

Optimized Structure and Computed Energies for *anti*-(2*R*,1′*R*)-1-Li [values are in Hartree] (6-31+G(d,p), IEF-PCM, solvent: THF)

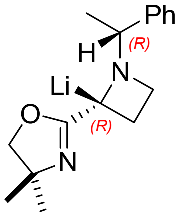

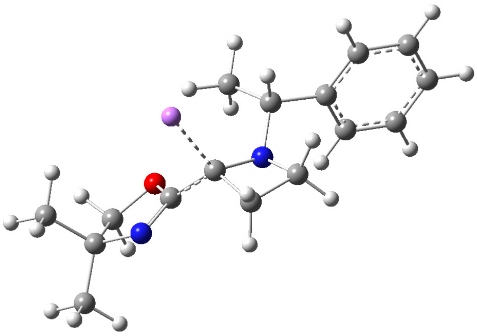

Method	Total Electronic Energy	Sum of Electronic and Zero-Point Energies	Sum of Electronic and Thermal Enthalpies	Gibbs Free Energy
B97XD	-814.4108866	-814.064770	-814.044588	-814.112307
B3LYP	-814.6501248	-814.309155	-814.288430	-814.358111
CAM-B3LYP	-814.1981645	-813.852470	-813.832819	-813.900051
B3PW91	-814.3378992	-813.996186	-813.975342	-814.045698
MPW1PW91	-814.461315	-814.116563	-814.095976	-814.166236
BVP86	-814.6703071	-814.339652	-814.319057	-814.388232
M0-62X	-814.2876614	-813.943452	-813.922801	-813.993033

PBEPBE	-813.5977908	-813.264956	-813.244121	-813.313490

Optimized Structure and Computed Energies for *syn*-(2*S*,1′*R*)-1-Li [values are in Hartree] (6-31+G(d,p), IEF-PCM, solvent: THF)

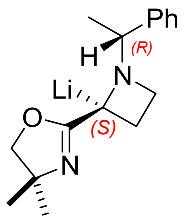

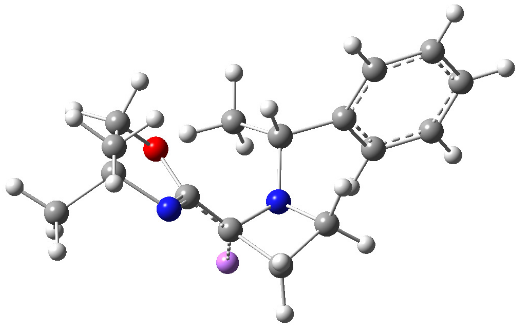

Method	Total Electronic Energy	Sum of Electronic and Zero-Point Energies	Sum of Electronic and Thermal Enthalpies	Gibbs Free Energy
B97XD	-814.4039745	-814.057351	-814.037301	-814.105239
B3LYP	-814.6493487	-814.307060	-814.286843	-814.354751
CAM-B3LYP	-814.1905763	-813.843782	-813.823838	-813.891433
B3PW91	-814.3364009	-813.993307	-813.973013	-814.041131
MPW1PW91	-814.4532567	-814.107713	-814.087576	-814.155394
BVP86	-814.6689045	-814.337120	-814.316117	-814.386070
M0-62X	-814.2875027	-813.941345	-813.921471	-813.988220
PBEPBE	-813.5905754	-813.257911	-813.237067	-813.306272

Conformational analysis (syn/anti) between oxazolinylazetidine ring and *N*-substituent inlithiated oxazolinylazetidine

Optimized Structure, Wavelength and Computed Energies [values are in Hartree]

(B97XD/6-31+G(d,p), solvent: THF)

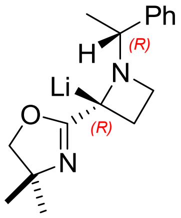

(*Syn*)

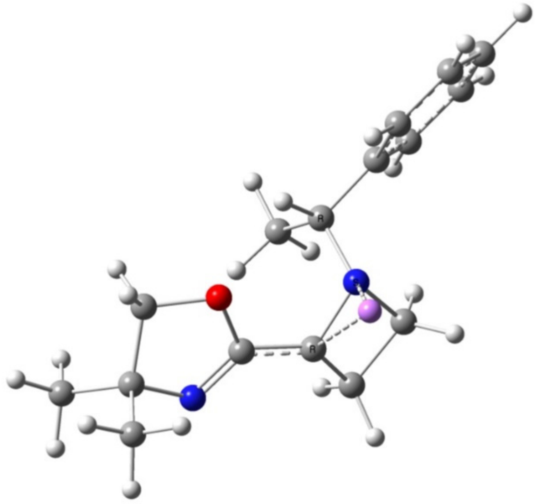

Solvation Model	Wavelength (cm^{-1}) for C=N bond	Total Electronic Energy	Sum of Electronic and Zero-Point Energies	Sum of Electronic and Thermal Enthalpies	Gibbs Free Energy
IEF-PCM	1657	-814.4068524	-814.059822	-814.040013	-814.106464
CPCM	1700	-814.412975	-814.066957	-814.046827	-814.114937

Cartesian Coordinates (B97XD/6-31+G(d,p), IEF-PCM, solvent: THF)

C	-0.47625000	2.54884900	-0.42821100
C	0.98854200	2.12006900	-0.66523100
C	-0.82110200	1.11033300	-0.73498700
H	-0.67101900	2.85793800	0.61094900
H	-0.85982100	3.32552300	-1.09765900
H	1.70620100	2.36579300	0.12088600
H	1.40317700	2.45165800	-1.62271600
N	0.61366700	0.66895400	-0.71646200
C	-1.89414400	0.33711000	-0.29630800
C	-3.91363100	-0.47557700	0.20582300
C	-2.89256100	-1.62942200	0.16051000
N	-3.04957400	0.70983900	0.19330400
O	-1.74528800	-1.04488400	-0.46235700
Li	-0.03353100	0.09496400	-2.51604300
H	-2.61826500	-1.96558300	1.16948000
H	-3.21137000	-2.48813600	-0.43622900

C	-4.81296900	-0.49434600	-1.04115400
C	-4.76855500	-0.51994600	1.47045900
H	-4.20497800	-0.51086300	-1.95275400
H	-5.47009900	-1.37260200	-1.04764700
H	-5.43519600	0.40564800	-1.06444900
H	-4.13546300	-0.47093300	2.36205900
H	-5.45465800	0.33270800	1.49129400
H	-5.36409400	-1.43941400	1.51358500
C	1.07456700	-0.14921300	0.43180600
H	0.58292200	-1.11837900	0.29745400
C	0.65155000	0.37628100	1.80844500
C	2.56957600	-0.37683300	0.28008500
C	3.01986700	-1.31419500	-0.65752900
C	3.52466900	0.33514000	1.00962100
C	4.37856900	-1.53157400	-0.86692000
H	2.28994300	-1.88512600	-1.22677500
C	4.88905100	0.12438500	0.80363900
H	3.21158300	1.06135700	1.75316300
C	5.32174000	-0.80771100	-0.13589900
H	4.70295600	-2.26853900	-1.59553800
H	5.61245200	0.69063700	1.38261100
H	6.38267100	-0.97462700	-0.29415100
H	1.04423800	1.37528400	2.02001000
H	-0.43825200	0.42899100	1.86833000
H	1.00780200	-0.29881400	2.59230600

Cartesian Coordinates (B97XD/6-31+G(d,p), CPCM, solvent: THF)

C	-0.55272200	2.44900300	-0.82625000
C	0.77206200	1.74824700	-1.21050900
C	-0.83908200	1.20242400	-0.02302800
H	-0.40771700	3.37975000	-0.26031700
H	-1.23164500	2.65793000	-1.66222100
H	1.69283600	2.29225300	-0.98472500
H	0.81591300	1.41276400	-2.25136200
N	0.48784900	0.59733500	-0.29154900
C	-1.97416600	0.43892300	0.10515000
C	-3.99800600	-0.52038800	0.01003100
C	-2.98031800	-1.55791200	0.52167800
N	-3.26024800	0.73868700	0.13116600
O	-1.71693200	-0.95827000	0.22887900
Li	-0.18558800	-1.15778400	-1.10110800
H	-3.06323900	-1.70419500	1.60645100
H	-3.03088000	-2.52538600	0.01562300

C	-4.35358400	-0.80762000	-1.46093100
C	-5.26983000	-0.51135200	0.85639700
H	-3.44364100	-0.86011100	-2.07004700
H	-4.89642500	-1.75501400	-1.56803300
H	-4.97964300	-0.00173600	-1.85679500
H	-5.03387300	-0.30507800	1.90517400
H	-5.95224600	0.26721000	0.50017400
H	-5.79232000	-1.47338300	0.79808100
C	1.38577400	0.46247600	0.88565900
H	0.87991500	-0.27396000	1.52114700
C	1.55220100	1.73440700	1.72157700
C	2.69373400	-0.16315300	0.42966800
C	2.78934700	-1.55716500	0.34121300
C	3.80269800	0.59790000	0.04765600
C	3.94789400	-2.17517500	-0.12338100
H	1.94259400	-2.16721200	0.64983100
C	4.96592900	-0.01395900	-0.42011500
H	3.77176900	1.68065600	0.11824100
C	5.04293700	-1.40227800	-0.50962800
H	3.99916000	-3.25847700	-0.17885800
H	5.81448600	0.59774200	-0.71158700
H	5.94912700	-1.87824100	-0.87107100
H	2.04997800	2.54340800	1.17939900
H	0.56477600	2.09161300	2.02630500
H	2.14088500	1.52055700	2.61844100

Optimized Structure, Wavelength and Computed Energies [values are in Hartree]

(B97XD/6-31+G(d,p), solvent: THF)

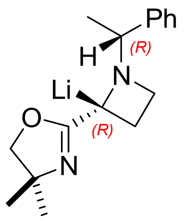

(*Anti*)

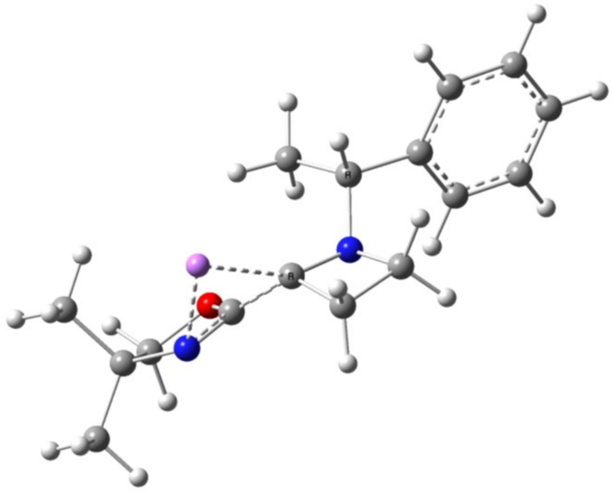

Solvation Model	Wavelength (cm^{-1}) for C=N bond	Total Electronic Energy	Sum of Electronic and Zero-Point Energies	Sum of Electronic and Thermal Enthalpies	Gibbs Free Energy
IEF-PCM	1613	-814.4108866	-814.064770	-814.044588	-814.112307
CPCM	1620	-814.414737	-814.068982	-814.048600	-814.117196

Cartesian Coordinates (B97XD/6-31+G(d,p), IEF-PCM, solvent: THF)

C	0.49854600	2.42263700	-0.15954100
C	-0.96963600	1.99215500	-0.36308500
C	0.74688800	0.95309700	0.18135300
H	0.99768500	2.76112600	-1.07676000
H	0.64744800	3.18899400	0.60947300
H	-1.43608200	2.25075300	-1.31853200
H	-1.62720000	2.32855000	0.45087500
N	-0.60506500	0.55782600	-0.24143000
C	1.92089100	0.27227000	-0.12770300
C	4.09159500	-0.33548700	-0.11043700
C	3.23439100	-1.41692800	-0.79602600
N	3.14706600	0.78119100	0.01234300
O	1.88665800	-1.05440200	-0.47629300
C	-1.42045400	-0.16971400	0.72730300
H	-1.39441400	0.36011900	1.70228100
C	-2.86807200	-0.22008200	0.27056600
C	-3.90442200	-0.02719500	1.18710200
C	-3.19534800	-0.49840200	-1.06058300
C	-5.23891800	-0.11927800	0.79159900
H	-3.66526100	0.19701000	2.22411700
C	-4.52670900	-0.58588100	-1.46201300
H	-2.39468400	-0.63626800	-1.78090400
C	-5.55432800	-0.39923000	-0.53663700
H	-6.02984500	0.03291700	1.52019000
H	-4.76273200	-0.79891300	-2.50056600
H	-6.59179000	-0.46919900	-0.84941800
C	-0.87965900	-1.58062000	0.95372000
H	-1.50115900	-2.10868500	1.68382400
H	-0.88096700	-2.14363900	0.01541100
H	0.14747300	-1.53900200	1.32479700
Li	2.45170000	1.53168000	1.77023100
H	3.35442400	-1.39857700	-1.88644700
H	3.41520000	-2.42959400	-0.42699100
C	4.53814300	-0.80486800	1.28445900
C	5.30843100	0.04719500	-0.95052100

H	3.67132900	-1.12461400	1.87531300
H	5.23901800	-1.64547900	1.22463500
H	5.03482800	0.01602600	1.81351600
H	4.99527600	0.40324700	-1.93669100
H	5.86753700	0.85036000	-0.45945700
H	5.98351700	-0.80608100	-1.08274200

Cartesian Coordinates (B97XD/6-31+G(d,p), CPCM, solvent: THF)

C	0.48611100	2.40455200	-0.13313000
C	-0.97944800	1.97224500	-0.34954000
C	0.74256400	0.92909900	0.16376500
H	0.98295100	2.77500200	-1.03965200
H	0.63098900	3.14883900	0.65846700
H	-1.44332200	2.24979800	-1.30088700
H	-1.64090600	2.28832900	0.46877100
N	-0.61020400	0.53501000	-0.25858500
C	1.92359900	0.26400800	-0.13656000
C	4.10346600	-0.31619600	-0.10605400
C	3.26328700	-1.41770300	-0.78085600
N	3.14567000	0.79128400	-0.00734800
O	1.90911400	-1.06845300	-0.47264800
C	-1.42001900	-0.20800600	0.70578100
H	-1.37599800	0.30057600	1.69052700
C	-2.87368400	-0.23360100	0.26605500
C	-3.89667000	-0.04091300	1.19776900
C	-3.22055500	-0.48375400	-1.06593100
C	-5.23690300	-0.10409000	0.81545700
H	-3.64270300	0.16212400	2.23552100
C	-4.55757100	-0.54199800	-1.45423800
H	-2.43116700	-0.62225600	-1.79850400
C	-5.57166400	-0.35468100	-0.51410300
H	-6.01722100	0.04819700	1.55531300
H	-4.80827400	-0.73290600	-2.49361700
H	-6.61356400	-0.40091200	-0.81624900
C	-0.88975600	-1.62820500	0.89451700
H	-1.50151000	-2.16351600	1.62753100
H	-0.91893600	-2.17406500	-0.05376200
H	0.14547000	-1.60509200	1.24372200
Li	2.47483600	1.53847400	1.76598600
H	3.38952300	-1.41296600	-1.87085100
H	3.45475300	-2.42303400	-0.39724500
C	4.55120500	-0.75830200	1.29800000
C	5.32140600	0.06693000	-0.94494700

H	3.68876800	-1.08848200	1.88959700
H	5.26976600	-1.58482100	1.25246600
H	5.02643800	0.08025600	1.81905500
H	5.01236000	0.39621700	-1.94182600
H	5.86403300	0.88890100	-0.46642600
H	6.01065200	-0.77835200	-1.05309700

Optimized Structure, Wavelength and Computed Energies [values are in Hartree]

(B97XD/6-31+G(d,p), solvent: THF)

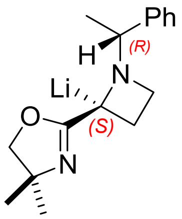

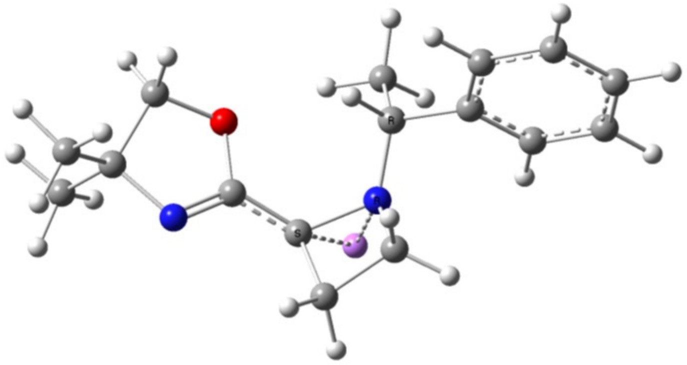

(*Syn*)

Solvation Model	Wavelength (cm⁻¹) for C=N bond	Total Electronic Energy	Sum of Electronic and Zero-Point Energies	Sum of Electronic and Thermal Enthalpies	Gibbs Free Energy
IEF-PCM	1615	-814.4039745	-814.057351	-814.037301	-814.105239
CPCM	1606	-814.4078133	-814.061536	-814.041366	-814.109370

Cartesian Coordinates (B97XD/6-31+G(d,p), IEF-PCM, solvent: THF)

C	0.51311600	-2.26553700	-1.12774900
C	-0.87194800	-1.60309000	-1.08791900
C	0.86124300	-1.41124100	0.09268900
H	0.48660200	-3.34848600	-0.95309000
H	1.10004500	-2.07161900	-2.02950000
H	-1.76468800	-2.23130400	-1.18318000
H	-0.93901200	-0.77671500	-1.81028200
N	-0.62230700	-1.09915700	0.28319100
C	-1.10862600	0.25217200	0.57942800
H	-0.51975600	0.99041500	0.01212800
C	-2.55965400	0.38708600	0.15118300
C	-2.96492200	1.45942300	-0.64544400
C	-3.52323300	-0.53843100	0.56723500
C	-4.30225300	1.61466500	-1.01232000
H	-2.22642300	2.18206000	-0.98311500
C	-4.85773500	-0.39278500	0.19849700

H	-3.21987300	-1.38405000	1.17818900
C	-5.25304700	0.68787500	-0.59158700
H	-4.59802300	2.45664400	-1.63102400
H	-5.59179000	-1.12259700	0.52681600
H	-6.29412500	0.80448100	-0.87657700
C	-0.97080000	0.55703400	2.07398400
H	-1.29137400	1.58180300	2.28404300
H	-1.60323100	-0.12186600	2.65709900
H	0.06898100	0.45498700	2.39015500
C	1.88932000	-0.44518400	0.00283700
C	2.96933300	1.44477400	0.56353800
C	3.88420400	0.52593600	-0.26673800
H	3.42569200	1.82664600	1.48041000
H	2.58896800	2.28490000	-0.03335600
N	2.93900200	-0.47526300	-0.77458400
O	1.87637500	0.59704600	0.92122800
C	4.56432900	1.27372600	-1.41051600
C	4.93875100	-0.14695400	0.62657100
H	3.81791900	1.72882500	-2.06894500
H	5.22345800	2.06260300	-1.02987300
H	5.16913100	0.58279700	-2.00637500
H	4.46042600	-0.64444100	1.47760000
H	5.48706300	-0.90191900	0.05444000
H	5.65709300	0.58564700	1.01401200
Li	0.02886500	-2.36368100	1.77273500

Cartesian Coordinates (B97XD/6-31+G(d,p), IEF-PCM, solvent: THF)

C	0.52621700	-2.28187400	-1.09408900
C	-0.84545000	-1.59068100	-1.10219300
C	0.86079700	-1.41258800	0.12169600
H	0.46868900	-3.36003800	-0.89896500
H	1.14161400	-2.11815800	-1.98276400
H	-1.74791500	-2.20107800	-1.22031200
H	-0.87266300	-0.76652700	-1.83064600
N	-0.62326200	-1.08597100	0.27116500
C	-1.10743800	0.26412300	0.56529500
H	-0.52277100	1.00332200	-0.00569300
C	-2.56083400	0.39215600	0.14364500
C	-2.97874200	1.46678700	-0.64317600
C	-3.51399200	-0.54554100	0.55724000
C	-4.31941400	1.61260200	-1.00269700
H	-2.24818000	2.19844800	-0.97872500
C	-4.85152400	-0.40907000	0.19586100

H	-3.19843300	-1.39304700	1.15954700
C	-5.25999800	0.67420200	-0.58432800
H	-4.62577300	2.45649800	-1.61355900
H	-5.57762300	-1.14764600	0.52222200
H	-6.30342100	0.78381800	-0.86342800
C	-0.96105600	0.56813900	2.05951500
H	-1.25240600	1.60195200	2.26796200
H	-1.60946700	-0.09259800	2.64606800
H	0.07588100	0.43615600	2.37467400
C	1.89121100	-0.44941300	0.01785800
C	2.96440500	1.45860700	0.53098900
C	3.88814100	0.51889400	-0.26504200
H	3.41234100	1.86774400	1.44007300
H	2.58704500	2.28017000	-0.09300400
N	2.94941900	-0.50046700	-0.74877200
O	1.87005500	0.61900700	0.90391300
C	4.57167100	1.23623600	-1.42641400
C	4.93961200	-0.12240600	0.65451100
H	3.82672800	1.66532900	-2.10398600
H	5.22127500	2.04170200	-1.06493500
H	5.18721300	0.53302900	-1.99660700
H	4.45797300	-0.59680200	1.51678300
H	5.49542600	-0.89131700	0.10846800
H	5.65216300	0.62431600	1.02475000
Li	-0.09095800	-2.35110000	1.77733400

Optimized Structure, Wavelength and Computed Energies [values are in Hartree]

(B97XD/6-31+G(d,p), solvent: THF)

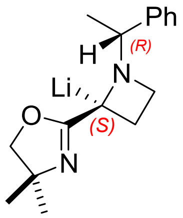

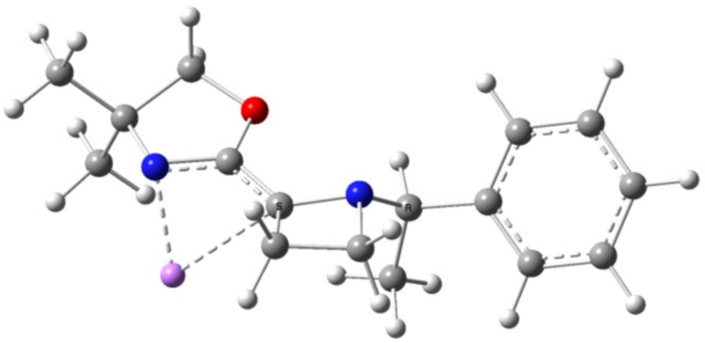

(*Anti*)

Solvation Model	Wavelength (cm^{-1}) for C=N bond	Total Electronic Energy	Sum of Electronic and Zero-Point Energies	Sum of Electronic and Thermal Enthalpies	Gibbs Free Energy

IEF-PCM	1686	-814.4060799	-814.059671	-814.039412	-814.108448
CPCM	1722	-814.4108456	-814.064493	-814.044255	-814.113504

Cartesian Coordinates (B97XD/6-31+G(d,p), IEF-PCM, solvent: THF)

C	0.43370500	2.11724200	1.26564300
C	-1.04672200	1.73962400	1.02317100
C	0.74545300	0.89998100	0.41246000
H	0.72158000	3.11465300	0.91480000
H	0.74648100	2.01923900	2.31387300
H	-1.51365900	2.38915900	0.27042400
H	-1.70514000	1.68314600	1.89514300
N	-0.63189000	0.41893300	0.49477200
C	1.89216100	0.15373300	0.30548700
C	3.12044400	-1.69825100	-0.02000100
C	4.04810000	-0.46453100	-0.00333500
H	3.28780000	-2.36961200	-0.86637800
H	3.19561400	-2.26782100	0.91519400
N	3.15247300	0.60582600	0.44444100
O	1.80084200	-1.15725500	-0.12564000
C	5.21667100	-0.65676700	0.96430500
C	4.58548900	-0.16482800	-1.41401200
H	4.84525000	-0.84476000	1.97610700
H	5.85188300	-1.49747100	0.66045900
H	5.83670100	0.24547700	0.99044800
H	3.75456200	-0.05860000	-2.12168200
H	5.15540800	0.77062700	-1.40521600
H	5.24391900	-0.96153000	-1.77963500
Li	2.55353800	2.09015800	-0.78119200
C	-1.30704000	-0.08879900	-0.71015900
C	-2.76786300	-0.32979000	-0.36171200
C	-3.11559000	-1.48191700	0.35313700
C	-3.77946200	0.57915600	-0.68153500
C	-4.43275400	-1.72330600	0.73401500
H	-2.33837700	-2.19501500	0.61485200
C	-5.10158800	0.34528100	-0.30007300
H	-3.54484100	1.48346000	-1.23457600
C	-5.43355200	-0.80681100	0.40846800
H	-4.67957500	-2.62735900	1.28281100
H	-5.87101100	1.06648300	-0.55938000
H	-6.46260400	-0.99162700	0.70124700
H	-0.85377600	-1.06849800	-0.90252600
C	-1.10126700	0.75352600	-1.97533500
H	-1.64084100	0.31235700	-2.81886600

H	-1.44177500	1.78733200	-1.85757200
H	-0.03613500	0.78335100	-2.21846900

Cartesian Coordinates (B97XD/6-31+G(d,p), CPCM, solvent: THF)

C	-0.42119500	2.24205100	-1.12124100
C	1.05690600	1.83444100	-0.90589400
C	-0.74256200	0.91093700	-0.47435100
H	-0.71500100	3.17483100	-0.62590700
H	-0.70868900	2.31500100	-2.17891300
H	1.51482000	2.37724000	-0.06917300
H	1.72623900	1.89045300	-1.76946300
N	0.63909900	0.45271700	-0.56009000
C	-1.88565700	0.17572600	-0.37077900
C	-3.10469300	-1.69796300	-0.14063200
C	-4.01920000	-0.46947100	0.01702400
H	-3.23543000	-2.45747200	0.63489600
H	-3.22574500	-2.16221700	-1.12875100
N	-3.16182600	0.62201700	-0.45626900
O	-1.78198400	-1.16676300	-0.03181800
C	-5.28746600	-0.58800500	-0.82646200
C	-4.39697800	-0.27322500	1.49849900
H	-5.03340300	-0.72239500	-1.88236600
H	-5.90536500	-1.43431800	-0.50384100
H	-5.88606800	0.32426000	-0.73289600
H	-3.49655200	-0.25282600	2.12474300
H	-4.93065200	0.67542800	1.62734800
H	-5.04442900	-1.07906100	1.86443900
Li	-2.66106000	2.11114200	0.78445600
C	1.26442800	-0.18283700	0.61315300
H	0.81814000	-1.18245500	0.67044900
C	0.97755300	0.50761400	1.95235500
C	2.74459900	-0.36607000	0.31734000
C	3.14797300	-1.42324000	-0.50672800
C	3.72306900	0.50729200	0.79889600
C	4.48763400	-1.60725900	-0.83865200
H	2.39662900	-2.10866600	-0.89070000
C	5.06758600	0.33057100	0.46819600
H	3.44389600	1.33532800	1.44302700
C	5.45489200	-0.72722200	-0.35091700
H	4.77835300	-2.43727900	-1.47575600
H	5.81136700	1.02208200	0.85296100
H	6.50123200	-0.86887000	-0.60366000
H	1.48820100	-0.01521600	2.76696500

H	1.29758800	1.55446000	1.96861200
H	-0.09832000	0.48918900	2.14484700
